# Prediction of Cardiovascular Disease Risk Accounting for Future Initiation of Statin Treatment

**DOI:** 10.1093/aje/kwab031

**Published:** 2021-02-17

**Authors:** Zhe Xu, Matthew Arnold, David Stevens, Stephen Kaptoge, Lisa Pennells, Michael J Sweeting, Jessica Barrett, Emanuele Di Angelantonio, Angela M Wood

**Keywords:** cardiovascular disease, electronic health records, future statin initiation, longitudinal data, risk prediction, treatment drop-in

## Abstract

Cardiovascular disease (CVD) risk-prediction models are used to identify high-risk individuals and guide statin initiation. However, these models are usually derived from individuals who might initiate statins during follow-up. We present a simple approach to address statin initiation to predict “statin-naive” CVD risk. We analyzed primary care data (2004–2017) from the UK Clinical Practice Research Datalink for 1,678,727 individuals (aged 40–85 years) without CVD or statin treatment history at study entry. We derived age- and sex-specific prediction models including conventional risk factors and a time-dependent effect of statin initiation constrained to 25% risk reduction (from trial results). We compared predictive performance and measures of public-health impact (e.g., number needed to screen to prevent 1 event) against models ignoring statin initiation. During a median follow-up of 8.9 years, 103,163 individuals developed CVD. In models accounting for (versus ignoring) statin initiation, 10-year CVD risk predictions were slightly higher; predictive performance was moderately improved. However, few individuals were reclassified to a high-risk threshold, resulting in negligible improvements in number needed to screen to prevent 1 event. In conclusion, incorporating statin effects from trial results into risk-prediction models enables statin-naive CVD risk estimation and provides moderate gains in predictive ability but had a limited impact on treatment decision-making under current guidelines in this population.

## Abbreviations

CI: confidence interval
CPRD: Clinical Practice Research Datalink
CVD: cardiovascular disease
HDL: high-density lipoprotein
NRI: net reclassification improvement
SBP: systolic blood pressure

***Editor’s note:** An invited commentary on this article appears on page 2015.*

Cardiovascular disease (CVD) remains the leading cause of morbidity and mortality worldwide (1). Identifying individuals who are at high CVD risk is important for effectively implementing prevention strategies with limited health-care resources (2). For this purpose, many prediction models have been developed and subsequently recommended by primary prevention guidelines to help identify individuals at high risk of CVD who should benefit the most from preventive interventions, such as lifestyle advice and statin treatment (3–17). As such, CVD risk-prediction models are typically intended for treatment-naive populations (i.e., for the assessment of CVD risk in the absence of future treatment initiation) (10); however, they are rarely developed and validated in populations that remain treatment-naive throughout follow-up (18–20). Indeed, most contemporary models have been developed using data that excluded statins users at baseline without taking into account statin initiation during follow-up (so-called “treatment drop-ins”) (19, 21), leading to a possible underestimation of risk and hence undertreatment of high-risk individuals (22). The problem of treatment drop-ins in risk-prediction modeling is underappreciated (23).

Given the absence of an ideal treatment-naive population in which to develop risk-prediction models, it is important to explore statistical methods that address treatment drop-in effects (23). Previous studies have investigated the use of inverse probability weighting (18) or marginal structural models (20) to enable the estimation of treatment-naive risks. However, these models require estimating an unbiased treatment effect within the study population, relying on randomized study designs or cohorts with no unmeasured confounders.

Here, we propose incorporating causal evidence from clinical trials to provide a novel and simple approach to address time-dependent treatment drop-in for the estimation of treatment-naive risks (interpretable as risk estimates in the absence of future treatment initiation) (23). We illustrated our simple and practical approach through the derivation and validation of a CVD risk model to estimate 10-year statin-naive CVD risk predictions, using longitudinal electronic health records from a large and representative UK population.

## METHODS

### Study population

#### Data source.

We used medical records from English National Health Service general practices that contributed anonymized primary-care electronic health records to the Clinical Practice Research Datalink (CPRD), covering approximately 6.9% of the UK population (24). Patients in CPRD are broadly representative of the UK general population with respect to age, sex, and ethnicity (24). CPRD was linked to secondary care admissions from Hospital Episode Statistics and national mortality records from the Office for National Statistics.

The data used in this study was obtained under license from the UK Medicines and Healthcare Products Regulatory Agency (protocol 162RMn2).

#### Study outcomes.

CVD was defined as a combination of new diagnoses of nonfatal or fatal events of coronary heart disease (including myocardial infarction and angina), stroke, and transient ischemic attack, matching the definition used by QRISK algorithm (8, 15), which is recommended by UK CVD risk assessment guidelines for 40- to 84-year-olds (25). Read codes (used to identify outcomes in CPRD) and *International Classification of Diseases*, *Tenth Revision*, codes (used to identify outcomes in primary or secondary diagnosis fields from Hospital Episode Statistics and in underlying or subordinate cause of death fields from the Office for National Statistics) are provided in the Web Appendix 1, Web Tables 1 and 2 (available at https://doi.org/10.1093/aje/kwab031). We defined incident CVD as the first occurrence of CVD in any of the 3 databases (CPRD, Hospital Episode Statistics, and Office for National Statistics).

#### Risk factors.

Conventional CVD risk factors (10, 26) were selected, and included systolic blood pressure (SBP), total cholesterol, high-density lipoprotein (HDL) cholesterol (for which details of measurements have been previously described (24)), hypertension treatment (yes/no ascertained from CPRD prescription information), smoking status (current smoker or not ascertained from CPRD Read codes), and previous diagnoses of diabetes (yes/no ascertained from CPRD Read codes (27)). Individuals were assumed to have hypertension treatment or diabetes for the rest of follow-up after their first prescription or diagnosis. In addition, we defined statin initiation as the date of first CPRD prescription (code list for CPRD prescription provided in Web Appendix 2, Web Table 3). The following biologically implausible risk-factor values were set to missing: SBP >250 mm Hg or <60 mm Hg; total cholesterol >20 mmol/L or <1.75 mmol/L; HDL cholesterol >3.1 mmol/L or <0.3 mmol/L (28, 29). Values of SBP, total cholesterol, and HDL cholesterol were standardized using sex-specific means and standard deviations.

#### Study entry and exit.

Individuals entered our study on the latest of 4 dates: the date of 6 months after registration at the general practice; the date the individual turned 40 years of age (note, prior information from age 30 years onward were extracted for these individuals); the date that the data for the practice were up to standard (30); or April 1, 2004, the date of introduction of the Quality and Outcomes Framework (31). Individuals were censored at the earliest date of the following: the individual’s death or the first incident CVD event; the date that the individual turned 85 years of age (note, follow-up data up to age 95 years were extracted for these individuals); the date of deregistration at the practice; the last contact date for the practice with CPRD; or November 30, 2017, the end of data availability.

**Table 1:** Characteristics of Participants Included in the Current Study^a^ From Clinical Practice Research Datalink, Hospital Episode Statistics, and the Office for National Statistics, England, United Kingdom, 2004–2017

**Characteristic**	**Men**			**Women**		
	**No. of Individuals With at Least 1 Measurement**	**Mean (SD)**	**%**	**No. of Individuals With at Least 1 Measurement**	**Mean (SD)**	**%**
Derivation data set	518,367			622,731		
Age at study entry, years	518,367	50.3 (12.6)		622,731	51.3 (13.5)	
Systolic blood pressure, mm Hg^b^	484,714	136.9 (18.5)		603,496	131.7 (20.4)	
Total cholesterol, mmol/L^b^	374,492	5.4 (1.0)		437,519	5.6 (1.1)	
HDL cholesterol, mmol/L^b^	348,176	1.3 (0.4)		405,960	1.6 (0.4)	
Current/ever smoker^c^	306,719		48.6	295,502		47.3
History of diabetes^c^	518,367		7.1	622,731		4.9
Prescription for antihypertensive medication^c^	518,367		35.1	622,731		38.8
Initiated statins after study entry	518,367		16.2	622,731		12.6
Experienced incident CVD event	518,367		7.4	622,731		5.2
Validation data set	244,239			293,390		
Age at study entry, years	244,239	50.5 (12.6)		293,390	51.5 (13.5)	
Systolic blood pressure, mm Hg	229,861	136.2 (18.4)		285,603	131.2 (20.3)	
Total cholesterol, mmol/L	174,843	5.4 (1.1)		203,461	5.6 (1.1)	
HDL cholesterol, mmol/L	159,466	1.3 (0.4)		184,901	1.6 (0.4)	
Current/ever smoker	144,130		47.9	139,896		45.9
History of diabetes	244,239		6.9	293,390		4.8
Prescription for antihypertensive medication	244,239		34.9	293,390		38.8
Initiated statins after study entry	244,239		16.0	293,390		12.5
Experienced incident CVD event	244,239		7.2	293,390		5.1

Abbreviations: CVD, cardiovascular disease; HDL, high-density lipoprotein; SD, standard deviation.

^a^ Included 1,678,727 individuals aged 40–85 years, without prevalent CVD or statin initiation at study entry, and with at least 1 measurement value of systolic blood pressure, total cholesterol, HDL cholesterol, or smoking status between their study entry and study exit dates.

^b^ Calculated using the first measurement values taken after study entry.

^c^ Recorded as “yes” if any of the measurement values showed “yes” throughout the follow-up time.

#### Study eligibility criteria.

Of the 2,589,074 individuals with linked data, those with CVD or statin treatment identified before study entry were excluded. We also excluded individuals who had no measurements of any of SBP, total cholesterol, HDL cholesterol, or smoking status between study entry and exit dates. A total of 1,678,727 individuals (762,606 men and 916,121 women) were included in the study (flowchart in Web Figure 1).

We randomly allocated 2/3 of practices (263 practices with 1,141,098 individuals) to the derivation data set and 1/3 of practices (135 practices with 537,629 individuals) to the validation data set.

### Statistical modeling

To utilize all available electronic health records data, we used a 2-stage landmark approach for the construction of 10-year CVD risk-prediction models (32). We briefly describe the methods here and provide more detail in Web Appendix 3, Web Figures 2–6. In the derivation data set, we developed 92 age- and sex-specific predictions models (i.e., for men and women and at ages 40, 41, 42, …, 85, denoted as “landmark ages”). Participants meeting the study eligibility constraints contributed to a model if they had no CVD diagnoses and no statin prescription before the landmark age. Ten-year crude CVD incidence rates and statin-initiation rates were calculated for each landmark age and sex.

In the first stage, to better utilize repeat risk factors and allow for incomplete data, error-free risk-factor values for SBP, total cholesterol, HDL cholesterol, and smoking status were estimated as best linear unbiased predictors (BLUPS) from landmark age- and sex-specific multivariate mixed-effects linear regression models (Web Appendix 3). In the second stage, 10-year statin-naive CVD risk was modeled using landmark age- and sex-specific Weibull models, with time since landmark age as the time scale and with the following risk factors: the most recently observed diabetes status and hypertension treatment status; estimated error-free risk-factor values for SBP, total cholesterol, HDL cholesterol, and smoking status; and a time-dependent effect of statin initiation constrained to a 25% risk reduction as reported from published meta-analyses of trials (33, 34). For example, in Stata (StataCorp LLC, College Station, Texas) this can be implemented by splitting the follow-up data at the time of statin initiation and using the offset option in the survival model (see example code in Web Appendix 3). Incorporating the effect of statins in this way ignores the potential error in the effect and assumes homogeneity in treatment effect (i.e., a 25% risk reduction for everyone) regardless of the time on statins and other characteristics. The Weibull distribution and proportional hazards assumptions were checked and verified (see Web Appendix 3, Web Figures 5 and 6). We also derived a standard model ignoring the effect of statin initiation.

In the validation data set, we predicted 10-year statin-naive and standard CVD risks, using risk-factor values estimated from the multivariate mixed-effects models.

### Assessment of model predictive performance

Performance measures for the standard CVD models ignoring statin initiation were calculated from comparisons between the predicted standard CVD risks and observed survival times and risks in the validation data set. To appropriately assess model performance, we compared statin-naive CVD risks against observed risks using counterfactual statin-naive survival times. Under the Weibull model, counterfactual survival times were estimated as: $t^\ast =\!{\big[{t}_s^v+\exp (0.75)\times \big({t}^v-{t}_s^v\big)\big]}^{1/v}$, where $t$ is the observed follow-up time; ${t}_s$ is the time of statin initiation (which equals *t* if not observed); $\exp (0.75)$ represents the effect of statins from trial results of 25% risk reduction; and $\nu$ is the shape parameter of the Weibull model estimated in the derivation data set. Further details are provided in Web Appendix 4. Several measures were used to assess the model and compare the performance in the validation data set (full definitions and the use of counterfactual statin-naive survival times in performance assessment are provided in Web Table 4). Calibration was assessed visually (35, 36) and with the calibration slope (35–38); predictive accuracy and explained variation were assessed using the Brier score and *R*^2^ respectively (36, 39, 40), and discrimination was assessed by the *D* statistic (39) and Harrell’s *C* index (35) with bootstrap standard errors. Reclassification measures, including the net reclassification improvement (NRI), with both continuous NRI (41) and categorical NRI (42) using the predicted 10-year risk cutoff at <10% and ≥10% (i.e., the threshold of recommended statin treatment in the current UK guidelines (25)), together with the integrated discrimination index (42), were used to compare the statin-naive and the standard CVD risks at ages 40, 50, 60, and 70 years. Potential public health impact, including the number needed to screen and number needed to treat to prevent 1 CVD event (38, 43, 44), were estimated under the assumption that statin treatment is allocated to individuals with 10-year CVD risk greater than 10% and reduces CVD risk by 25%. In addition, to quantify the impact of models accounting for statin initiation on treatment decision-making, we compared the proportion of individuals with 10-year predicted risk exceeding a range of treatment thresholds from 5% to 30% by using the statin-naive versus the standard CVD risk for each landmark age. Weighted proportions across all ages were calculated using the most recent available data for an age- and sex-standard English population (2015) (45) aged 40–85 years. To directly demonstrate the predictive ability of statin-naive CVD risk, measures of model performance were also assessed on the subset of individuals with no statin initiation during follow-up.

**Figure 1:**
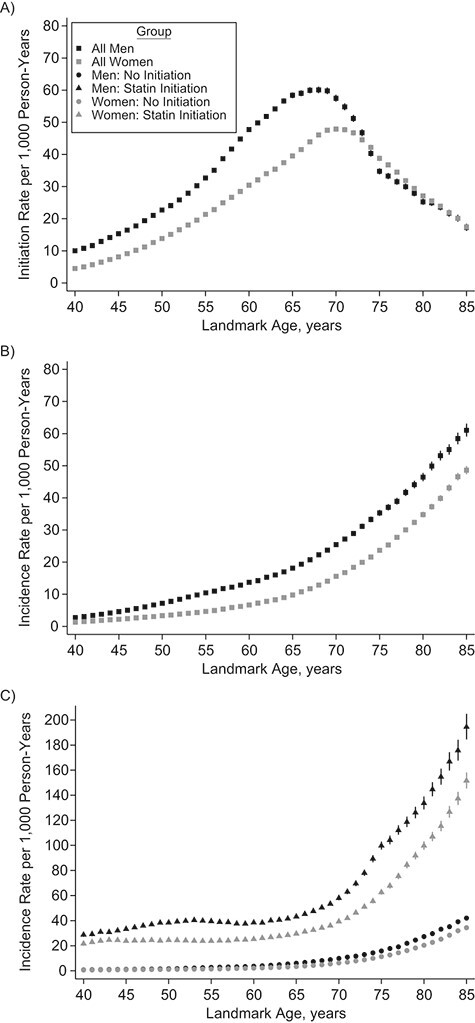
Sex-specific statin-initiation rates in the next 10 years (A), 10-year cardiovascular disease incidence rates (B), and 10-year cardiovascular disease incidence rates according to statin-initiation status in the next 10 years (C) by landmark age, with 95% confidence intervals (shown as vertical lines), Clinical Practice Research Datalink, Hospital Episode Statistics, and the Office for National Statistics, England, United Kingdom, 2004–2017.

**Figure 2:**
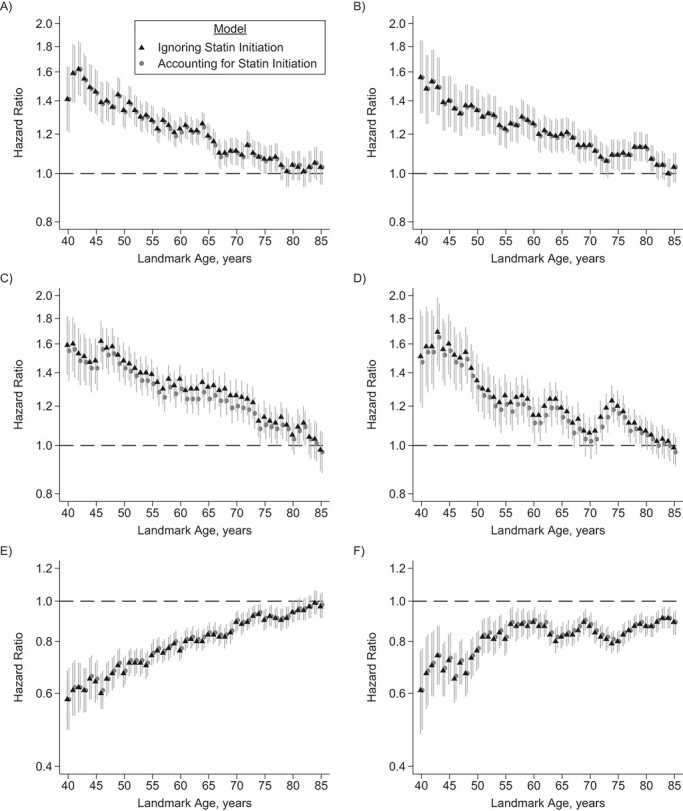
Hazard ratios and 95% confidence intervals (shown as vertical lines) for association of systolic blood pressure (SBP) with cardiovascular disease risk for men (A) and women (B), total cholesterol with cardiovascular disease risk for men (C) and women (D), and high-density lipoprotein (HDL) cholesterol with cardiovascular disease risk for men (E) and women (F) in the derivation data set, from models ignoring statin initiation versus models accounting for statin initiation for the prediction of 10-year cardiovascular disease risk by landmark age, Clinical Practice Research Datalink, Hospital Episode Statistics, and the Office for National Statistics, England, United Kingdom, 2004–2017. Hazard ratios are given per standard-deviation increase for SBP, total cholesterol, and HDL cholesterol. Hazard ratios and 95% confidence intervals are shown on the natural log scale.

All statistical analyses were conducted using Stata, version 15.1 (StataCorp LLC), and R, version 3.6.1 (R Foundation for Statistical Computing, Vienna, Austria). The 2-sided *P* value threshold was <0.05, and we calculated 95% confidence intervals.

## RESULTS

### Characteristics of participants

At study entry, the mean age was 50.9 (standard deviation, 13.1) years, and 45% of the participants were men. Characteristics of participants in the derivation and validation data sets were similar (Table 1). The median follow-up was 8.9 years (interquartile range, 5.3–11.4), during which 237,806 individuals initiated statins and there were 103,163 incident CVD events (Web Figure 7).

### Statin initiation and CVD incidence rates

Ten-year statin-initiation rates were higher in men, increased with age until approximately 70 years, and then declined (Figure 1). The overall 10-year CVD incidence rate was 7.39 (95% confidence interval (CI): 7.34, 7.43) per 1,000 person-years. The 10-year CVD incidence rates increased rapidly after age 65, and were higher in men and those who initiated statins during follow-up (Figure 1 and Web Table 5). Rates were broadly similar in the derivation and validation data sets (Web Tables 6 and 7).

**Figure 3:**
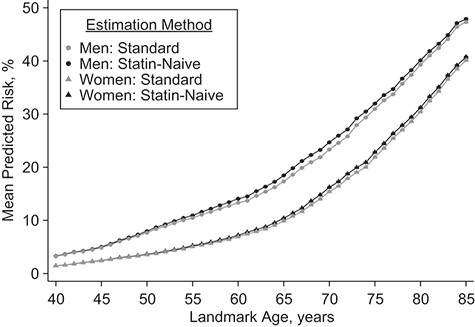
Comparison of sex-specific means of the statin-naive 10-year cardiovascular disease risk predictions and the standard 10-year cardiovascular disease risk predictions by landmark age in the validation data set, Clinical Practice Research Datalink, Hospital Episode Statistics, and the Office for National Statistics, England, United Kingdom, 2004–2017.

**Table 2:** Overall Brier Score^a^^,^^b^ and *C* Index^a^ in the Validation Data Set, Clinical Practice Research Datalink, Hospital Episode Statistics, and the Office for National Statistics, England, United Kingdom, 2004–2017

**Subgroup**	**Brier Score**	**95% CI**	***C* Index**	**95% CI**
Men				
Model ignoring statin initiation^c^	0.3671	0.3652, 0.3689	0.7388	0.7369, 0.7407
Model accounting for statin initiation^d^	0.3599	0.3581, 0.3618	0.7411	0.7392, 0.7430
Difference	−0.0071	−0.0073, −0.0070	0.0023	0.0021, 0.0024
Women				
Model ignoring statin initiation	0.2783	0.2767, 0.2800	0.7872	0.7853, 0.7891
Model accounting for statin initiation	0.2747	0.2730, 0.2763	0.7890	0.7871, 0.7909
Difference	−0.0037	−0.0038, −0.0036	0.0018	0.0017, 0.0020

Abbreviation: CI, confidence interval.

^a^ Overall Brier score and *C* index were calculated by “stacking” the data at each landmark age into a single data set (the stacked data set).

^b^ Brier scores were calculated only with information from individuals with at least 10 years follow-up or who had an event within 10 years from each landmark age.

^c^ Model ignoring statin initiation: ignoring statin treatment drop-in effect on CVD risk prediction.

^d^ Model accounting for statin initiation: accounting for statin treatment drop-in effect on CVD risk prediction.

### Risk factors associations with incident CVD

Hazard ratios for CVD attenuated at older landmark ages for all risk factors (Figure 2). Hazard ratios for total cholesterol and diabetes were somewhat higher in models accounting for statin initiation during follow-up (particularly for ages 60–70) compared with models ignoring statin initiation, but were similar for other CVD risk factors (Figure 2, Web Tables 8 and 9).

### Predicted 10-year CVD risk accounting for future statin initiation

In the validation data set, the means of 10-year statin-naive CVD risks were slightly higher than standard CVD risks (Figure 3, Web Table 10), especially among 60- to 70-year-olds. For example, in 65-year-old men the mean predicted standard and statin-naive 10-year CVD risks were 17.3% (95% CI: 17.3%, 17.4%) and 18.5% (95% CI: 18.4%, 18.5%), respectively. Similarly, in 65-year-old women, the corresponding mean risks were 9.91% (95% CI: 9.87%, 9.94%) and 10.4% (95% CI: 10.4%, 10.5%). The medians and interquartile ranges of predicted standard and statin-naive risks are shown in Web Table 11 and Web Figure 8.

### Model calibration, performance, and discrimination

**Figure 4:**
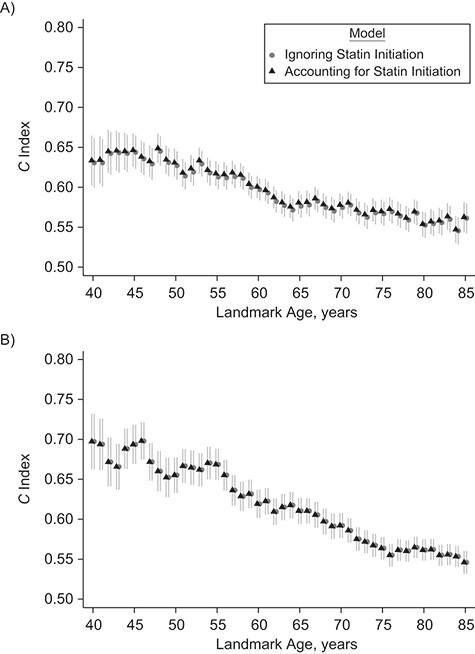
*C* indexes and 95% confidence intervals (shown as vertical lines) from models ignoring statin initiation versus models accounting for statin initiation for the prediction of 10-year cardiovascular disease risk by landmark age for men (A) and women (B) in the validation data set, Clinical Practice Research Datalink, Hospital Episode Statistics, and the Office for National Statistics, England, United Kingdom, 2004–2017.

The models appeared generally well calibrated, especially at younger ages (Web Figures 9–11). Compared against the models ignoring statin initiation, models accounting for statin initiation generally exhibited better model performance and discrimination, quantified by lower values for overall Brier score (Table 2), higher explained variation (Web Figure 12), and higher overall *C* index (Table 2) and *D* measure (Web Figure 13). The age-specific *C* indexes were higher in women, decreased with age, and were slightly higher in models accounting for statin initiation, especially for ages 60–70 (Figure 4). 

### Public health modeling

#### Reclassification.

There were moderate improvements in risk classification using 10-year statin-naive versus standard CVD predictions. Generally, individuals with future CVD events within 10 years were more likely to be reclassified from <10% to ≥10% risk categories (quantified by the categorical NRI) and have higher predicted risks (quantified by the category-free integrated discrimination index and continuous NRI) than were individuals who remained CVD event-free for 10 years (Tables 3–6). Above the ages of 69 for men and 76 for women, all predicted 10-year statin-naive and standard CVD risks were greater than 10%.

#### Potential public health impact.

**Figure 5:**
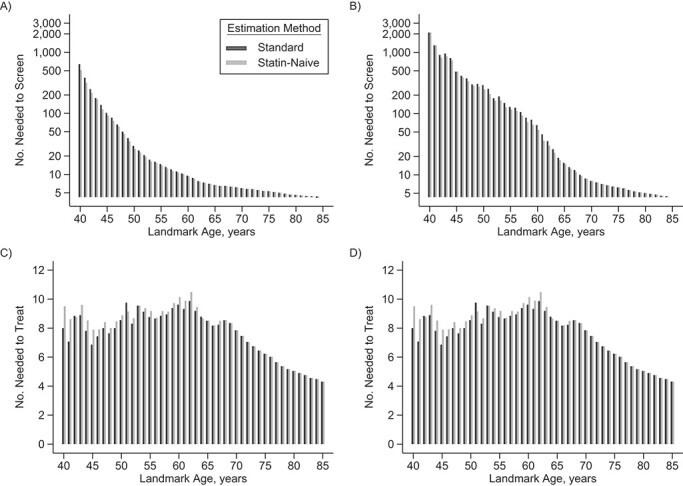
Number needed to screen to prevent 1 cardiovascular disease event for men (A) and women (B) and number needed to treat to prevent 1 cardiovascular disease event for men (C) and women (D), among people in the risk set at each landmark age model, using standard 10-year cardiovascular risk predictions versus statin-naive 10-year cardiovascular risk predictions, in the validation data set, Clinical Practice Research Datalink, Hospital Episode Statistics, and the Office for National Statistics, England, United Kingdom, 2004–2017. Numbers needed to screen to prevent 1 event are shown on the natural log scale.

**Figure 6:**
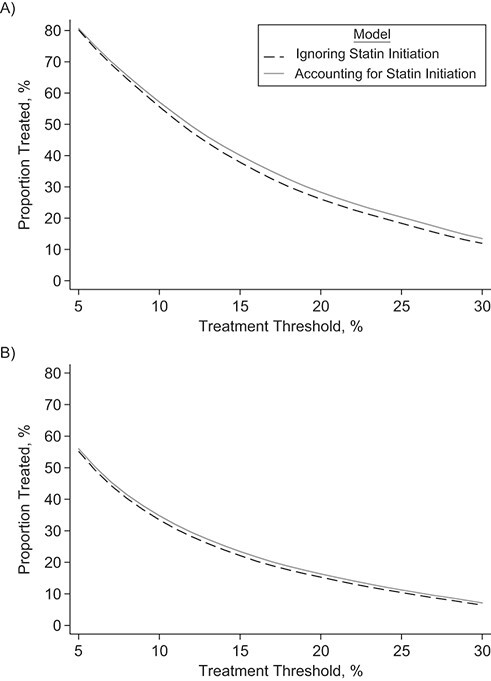
Proportion of individuals with 10-year predicted risk exceeding a range of treatment thresholds from 5% to 30% using the statin-naive versus the standard cardiovascular disease risk, for men (A) and women (B) in the validation data set, Clinical Practice Research Datalink, Hospital Episode Statistics, and the Office for National Statistics, England, United Kingdom, 2004–2017. Weighted proportions across all ages were calculated using the most recent available data for an age- and sex-standard England population aged 40–85 years.

Fewer younger people needed to be screened to prevent 1 CVD event using statin-naive compared with standard 10-year CVD risk predictions (Figure 5, Web Table 12). Above age 60, the number needed to be screened to prevent 1 event was generally similar between the 2 risk predictions, as well as for the number needed to treat to prevent 1 event (Figure 5). The weighted proportions across all ages of individuals with 10-year predicted risk exceeding treatment threshold were slightly higher after accounting for statin initiation (Figure 6). For example, at the threshold of ≥10%, the proportions were 55.6% in men and 33.5% in women using models ignoring statin initiation, and they were 57.1% in men and 34.8% in women using models accounting for statin initiation correspondingly.

**Table 3:** Ten-Year Cardiovascular Disease Risk Classification Comparing Statin-Naive Risk Predictions Versus Standard Risk Predictions for Men in the Validation Data Set (in Landmark Ages at 40, 50, 60, and 70 Years^a^), Clinical Practice Research Datalink, Hospital Episode Statistics, and the Office for National Statistics, England, United Kingdom, 2004–2017

**Standard 10-Year CVD Risk Prediction**	**Statin-Naive 10-Year CVD Risk Predictions**		
	**<10%**	**≥10%**	**Total**
*Landmark Age 40 Years*			
Events within 10 years^b^			
<10%	413	4	417
≥10%	0	16	16
Subtotal	413	20	433
Event-free at 10 years^b^			
<10%	1,965	3	1,968
≥10%	0	13	13
Subtotal	1,865	16	1,981
*Landmark Age 50 Years*			
Events within 10 years			
<10%	764	55	819
≥10%	0	480	480
Subtotal	764	535	1,299
Event-free at 10 years			
<10%	1,368	59	1,427
≥10%	0	390	390
Subtotal	1,368	449	1,817
*Landmark Age 60 Years*			
Events within 10 years			
<10%	187	55	242
≥10%	0	1,743	1,743
Subtotal	187	1,798	1,985
Event-free at 10 years			
<10%	147	62	209
≥10%	0	1,271	1,271
Subtotal	147	1,333	1,480
*Landmark Age 70 Years*			
Events within 10 years			
<10%	0	0	0
≥10%	0	1,901	1,901
Subtotal	0	1,901	1,901
Event-free at 10 years			
<10%	0	0	0
≥10%	0	613	613
Subtotal	0	613	613

Abbreviation: CVD, cardiovascular disease.

^a^ The results are presented in 10-year increments in landmark age at 40, 50, 60, and 70. Above landmark age 69 for men, the predicted 10-year CVD risk for all individuals in the risk set was greater than 10% for both standard risk predictions and statin-naive risk predictions; therefore, there was no movement between the 2 categories for those older landmark age groups.

^b^ Events within 10 years and event-free at 10 year for the reclassification table were defined using the counterfactual follow-up time, assuming statin had not been initiated.

**Table 4:** Ten-Year Cardiovascular Disease Risk Classification Comparing Statin-Naive Risk Predictions Versus Standard Risk Predictions for Women in the Validation Data Set (in Landmark Ages at 40, 50, 60, and 70 Years^a^), Clinical Practice Research Datalink, Hospital Episode Statistics, and the Office for National Statistics, England, United Kingdom, 2004–2017

**Standard 10-Year CVD Risk Predictions**	**Statin-Naive 10-Year CVD Risk Predictions**		
	**<10%**	**≥10%**	**Total**
*Landmark Age 40 Years*			
Events within 10 years^b^			
<10%	329	0	329
≥10%	0	8	8
Subtotal	329	8	337
Event-free at 10 years			
<10%	3,759	3	3,762
≥10%	0	6	6
Subtotal	3,759	9	3,768
*Landmark Age 50 Years*			
Events within 10 years			
<10%	723	10	733
≥10%	0	59	59
Subtotal	723	69	792
Event-free at 10 years			
<10%	3,073	10	3,083
≥10%	0	43	43
Subtotal	3,073	53	3,126
*Landmark Age 60 Years*			
Events within 10 years			
<10%	948	54	1,002
≥10%	0	277	277
Subtotal	948	331	1,279
Event-free at 10 years			
<10%	2,180	82	2,262
≥10%	0	226	226
Subtotal	2,180	308	2,488
*Landmark Age 70 Years*			
Events within 10 years			
<10%	19	8	27
≥10%	0	1,634	1,634
Subtotal	19	1,642	1,661
Event-free at 10 years			
<10%	7	2	9
≥10%	0	1,084	1,084
Subtotal	7	1,086	1,093

Abbreviation: CVD, cardiovascular disease.

^a^ The results are presented in 10-year increments in landmark age at 40, 50, 60, and 70. Above landmark age 76 for women, the predicted 10-year CVD risk for all individuals in the risk set was greater than 10% for both standard risk predictions and statin-naive risk predictions; therefore, there was no movement between the 2 categories for those older landmark age groups.

^b^ Events within 10 years and event-free at 10 years for the reclassification table were defined using the counterfactual follow-up time, assuming statin had not been initiated.

**Table 5:** Reclassification Measures for 10-Year Cardiovascular Disease Risk Prediction Comparing Statin-Naive Risk Predictions Versus Standard Risk Predictions for Men in the Validation Data Set (in Landmark Ages at 40, 50, 60, and 70 Years^a^), Clinical Practice Research Datalink, Hospital Episode Statistics, and the Office for National Statistics, England, United Kingdom, 2004–2017

**Outcome**	**Categorical NRI** ^b^ ^**,**^ ^c^	**95% CI**	**IDI** ^b^	**95% CI**	**Continuous NRI** ^d^	**95% CI**
*Landmark Age 40 Years*						
Event^b^^,^^e^	0.0092	0.0002, 0.0183	0.0013	0.0011, 0.0016	0.8939	0.8394, 0.9484
Nonevent^b^^,^^e^	−0.0015	−0.0032, 0.0002	−0.0006	−0.0007, −0.0006	−0.8665	−0.8716, −0.8614
Overall	0.0077	−0.0015, 0.0169	0.0007	0.0005, 0.0009	0.0274	−0.0294, 0.0843
*Landmark Age 50 Years*						
Event	0.0423	0.0312, 0.0535	0.0043	0.0040, 0.0046	0.9168	0.8853, 0.9482
Nonevent	−0.0325	−0.0408, −0.0242	−0.0027	−0.0028, −0.0026	−0.8734	−0.8786, −0.8683
Overall	0.0099	−0.0041, 0.0238	0.0016	0.0012, 0.0019	0.0433	0.0095, 0.0772
*Landmark Age 60 Years*						
Event	0.0277	0.0204, 0.0350	0.0095	0.0092, 0.0098	0.9736	0.9623, 0.9848
Nonevent	−0.0419	−0.0523, −0.0315	−0.0075	−0.0077, −0.0072	−0.9638	−0.9672, −0.9604
Overall	−0.0142	−0.0269, −0.0014	0.0020	0.0016, 0.0024	0.0098	−0.0036, 0.0231
*Landmark Age 70 Years*						
Event	0.0000	0.0000, 0.0000	0.0157	0.0153, 0.0160	0.9824	0.9690, 0.9958
Nonevent	0.0000	0.0000, 0.0000	−0.0142	−0.0147, −0.0137	−0.9849	−0.9900, −0.9798
Overall	0.0000	0.0000, 0.0000	0.0015	0.0008, 0.0021	−0.0025	−0.0200, 0.0149

Abbreviations: CI, confidence interval; CVD, cardiovascular disease; IDI, integrated discrimination improvement; NRI, net reclassification improvement.

^a^ The results are presented in 10-year increments in landmark age at 40, 50, 60, 70. Above landmark age 69 for men, the predicted 10-year CVD risk for all individuals in the risk set was greater than 10% for both standard risk predictions and statin-naive risk predictions; therefore, there was no movement between the 2 categories and the categorical NRIs were 0 for those older landmark age groups.

^b^ Categorical NRI and IDI were calculated using information from individuals who were not censored at 10 years (either with CVD events within 10 years or event-free at 10-years). Events within 10 years and event-free at 10 years, for the calculation of categorical NRI and IDI, were defined using the counterfactual follow-up time assuming statin had not been initiated.

^c^ Categorical NRI was calculated based on the 4 categories of predicted risk of <10% and ≥10%.

^d^ Continuous NRI (the prospective form NRI) was calculated based on continuous predicted risk and used information from all individuals, including the censored subjects.

^e^ Events and nonevents for continuous NRI (the prospective form of NRI) were the expected results estimated using the Kaplan-Meier approach with counterfactual follow-up time assuming statin had not been initiated, so this prospective form of NRI uses the whole sample and does not require the restriction to the noncensored subjects.

**Table 6:** Reclassification Measures for 10-Year Cardiovascular Disease Risk Prediction Comparing Statin-Naive Risk Predictions Versus Standard Risk Predictions for Women in the Validation Data Set (in Landmark Ages at 40, 50, 60, 70 Years^a^), Clinical Practice Research Datalink, Hospital Episode Statistics, and the Office for National Statistics, England, United Kingdom, 2004–2017

**Outcome**	**Categorical NRI** ^b^ ^**,**^ ^c^	**95% CI**	**IDI** ^b^	**95% CI**	**Continuous NRI** ^d^	**95% CI**
*Landmark Age 40 Years*						
Event^b^^,^^e^	0.0000	0.0000, 0.0000	0.0007	0.0005, 0.0010	0.8620	0.8156, 0.9084
Nonevent^b^^,^^e^	−0.0008	−0.0017, 0.0001	−0.0002	−0.0002, −0.0002	−0.7530	−0.7587, −0.7474
Overall	−0.0008	−0.0017, 0.0001	0.0006	0.0003, 0.0008	0.1090	0.0619, 0.1562
*Landmark Age 50 Years*						
Event	0.0126	0.0048, 0.0205	0.0021	0.0018, 0.0023	0.8459	0.7783, 0.9135
Nonevent	−0.0032	−0.0052, −0.0012	−0.0009	−0.0009, −0.0008	−0.8095	−0.8150, −0.8041
Overall	0.0094	0.0014, 0.0175	0.0012	0.0009, 0.0015	0.0364	−0.0339, 0.1067
*Landmark Age 60 Years*						
Event	0.0422	0.0310, 0.0535	0.0043	0.0040, 0.0045	0.9455	0.9299, 0.9611
Nonevent	−0.0330	−0.0401, −0.0258	−0.0029	−0.0030, −0.0028	−0.9081	−0.9123, −0.9038
Overall	0.0093	−0.0041, 0.0226	0.0014	0.0011, 0.0017	0.0374	0.0204, 0.0544
*Landmark Age 70 Years*						
Event	0.0048	−0.0012, 0.0072	0.0098	0.0095, 0.0101	0.9765	0.9640, 0.9890
Nonevent	−0.0018	−0.0044, 0.0007	−0.0083	−0.0085, −0.0080	−0.9675	−0.9716, −0.9633
Overall	0.0030	−0.0012, 0.0072	0.0015	0.0011, 0.0019	0.0090	−0.0061, 0.0241

Abbreviations: CI, confidence interval; CVD, cardiovascular disease; IDI, integrated discrimination improvement; NRI, net reclassification improvement.

^a^ The results are presented in 10-year increments in landmark age at 40, 50, 60, and 70. Above landmark age 76 for women, the predicted 10-year CVD risk for all individuals in the risk set was greater than 10% for both standard risk predictions and statin-naive risk predictions; therefore, there was no movement between the 2 categories and the categorical NRIs were 0 for those older landmark age groups.

^b^ Categorical NRI and IDI were calculated using information from individuals who were not censored at 10 years (either with CVD events within 10 years or event-free at 10 years). Events within 10 years and event-free at 10 years, for the calculation of categorical NRI and IDI, were defined using the counterfactual follow-up time assuming statin had not been initiated.

^c^ Categorical NRI was calculated based on the 4 categories of predicted risk of <10% and ≥10%.

^d^ Continuous NRI (the prospective form NRI) was calculated based on continuous predicted risk and used information from all individuals, including the censored subjects.

^e^ Events and nonevents for continuous NRI (the prospective form of NRI) were the expected results estimated using the Kaplan-Meier approach with counterfactual follow-up time assuming statin had not been initiated, so this prospective form of NRI uses the whole sample and does not require the restriction to the noncensored subjects.

Results were similar when analyses were performed using a validation subset including 463,017 individuals who did not initiate statins during the follow-up (Web Tables 13–16; Web Figures 14–21).

## DISCUSSION

In this study, we described a novel and simple approach to account for statin initiation for the prediction of 10-year statin-naive CVD risk, illustrated using primary-care data collected in a general UK population, and it is applicable to other study designs with similar information. Our analyses showed that, after adding a time-dependent effect of statin initiation constrained to a 25% CVD risk reduction, 10-year CVD predicted risks were higher, especially among 60- to 70-year-olds. These differences reflect the somewhat stronger associations between total cholesterol and CVD outcome after accounting for statin initiation and are in line with what is expected in a statin-naive population. Models that accounted for statin initiation also showed moderate improvements in calibration and discrimination but translated into limited public health and clinical relevance in our study population.

Currently recommended CVD risk-prediction models do not consider the effect of statin treatment drop-in during follow-up (19) and produce standard 10-year CVD risk estimates that are often interpreted in clinical practice, by practitioners and patients, as statin-naive CVD risk predictions (18–21). In this study, we found stronger hazard ratios for total cholesterol in models accounting for statin initiation, a phenomenon previously described as an “intervention effect” in clinical prediction models (46). Despite our study showing that statin-naive CVD risk predictions are generally higher than standard CVD risk predictions, we found little benefit in their use for clinical decision-making in this population of 40- to 85-year-olds. Accounting for statin initiation made the largest difference to risk estimates for individuals aged 60–70 (i.e., those more likely to start statins); however, a large proportion of these individuals were already categorized as being in a high-risk group (≥10%) on the basis of their age. Greater public health impact might be found 1) in other populations with higher statin-initiation rates or with higher CVD risk (e.g., diabetic patients), 2) with models using more conservative CVD endpoint definitions in risk model derivation (10), and/or 3) with use of age-specific risk thresholds (although these are not currently recommended by clinical guidelines).

Previous studies have attempted to account for statin drop-in by modeling the probability of statin initiation during follow-up (based on baseline risk factors), either through inverse probability weighting (18) or in marginal structural models (20). If the propensity model is incorrectly specified, then it might not fully account for the treatment drop-in. By contrast, our simpler approach incorporated causal evidence of 25% risk reduction with statin initiation from trial results. A similar approach using a time-fixed constrained treatment effect has been applied to estimate medication efficacy in long-term clinical trials (47) and in breast cancer prognostic models (48), as well as to adjust population-level incidence rates for CVD (33). However, to our knowledge, incorporating time-dependent statin treatment effects (which results in adjustment of risk-factor coefficients) for the prediction of the statin-naive 10-year CVD risk has not been fully explored and is aligned with the “hypothetical strategy” described previously (23). Our study assumed the same risk-reduction effect for all individuals regardless of treatment duration and discontinuations. It is possible to extend our model to allow for individuals’ risk reduction in response to statin initiation to vary by dose, treatment duration, and other demographic and socioeconomic factors (49, 50) which in combination might result in individuals having larger or smaller changes in risk, although on average likely to be smaller than we have modeled.

In our study, we found a reduction in the prediction ability of CVD risk-prediction models at older ages, due to attenuating hazard ratios of conventional risk factors (irrespective of whether statin treatment drop-in was accounted for). Previous assessments of the Framingham Risk Score also noted poorer performance in older individuals (51, 52). This was mainly attributed to older individuals still in the risk set being a homogeneous group in whom conventional CVD risk factors have little impact (36). This highlights the need to assess new CVD biomarkers across different age groups.

Our study has several strengths. This study proposed a simple approach to account for statin treatment drop-in and assessed it using CVD risk factors and events recorded in a large and representative UK population data set combining primary and secondary care health records. The landmark framework allowed us to optimally use repeated measurements of risk factors recorded in electronic health-records data and to assess the changes in hazard ratios and discrimination with age when accounting for statin initiation. Multivariate mixed-effects models allowed estimation of error-free risk-factor values at each landmark age, even when some risk factors were not observed, avoiding nonrepresentative “complete-case” analyses. In addition, a parametric Weibull model allowed a closed-form estimation of counterfactual statin-naive survival times, further allowing for model performance assessment in the “statin- naive” setting, with consistent results using the subset of individuals who remained statin-naive during follow-up. Our landmark models are easy to derive in standard software and to use in practice. We focused on the effect of statin drop-in, but the approach is generalizable to other causal relationships occurring in follow-up, such as short-term medications (e.g., corticosteroids), long-term medications (e.g., hypertension treatment), and lifestyle modification changes (e.g., smoking and smoking cessation), as well as other diseases.

Our study also has limitations that should be noted. Our data contains records only of statin prescriptions, with no information about treatment adherence, and so statin users might be incorrectly classified or indeed be treatment “drop-outs” as the proportion of people with poor adherence for statins might not be negligible (53). We also ignored any impact of informative observations, whereby more risk-factor measurements are made in sicker individuals who visit their general practitioners more frequently, or in the “worried-well” (54, 55); however, our previous work found that adjusting for the rate of general practitioner visits had negligible impact (32). We further ignored uncertainty in the constrained effect of statins, which might lead to slight overprecision in other estimated parameters. Additionally, the use of the Weibull model relies on strong parametric assumptions and is less flexible than the commonly used Cox model. It is possible to estimate counterfactual survival times in a Cox model with additional efforts (outlined in the Web Appendix 4). However, these limitations are unlikely to affect the between-model comparisons in prediction performance.

In conclusion, information from trials of the statins effect on CVD risk reduction can be simply incorporated into the derivation of risk models using electronic health records and yields statin-naive risk estimates interpretable as risk in the absence of future statin initiation. In our study population, accounting for statin initiation moderately improved measures of calibration and discrimination but had limited benefits for clinical decision-making under current UK guidelines of recommended statin-initiation threshold.

## Supplementary Material

Web_Material_kwab031
