# Supplementary material for: Prediction of Cardiovascular Disease Risk Accounting for Future Initiation of Statin Treatment
Source: Am J Epidemiol. 2021 Feb 17;190(10):2000–14. doi: 10.1093/aje/kwab031 (PMC8485151; doi:10.1093/aje/kwab031)
Supplement: Web_Material_kwab031 [file web_material_kwab031.pdf]

Web Material

Prediction of Cardiovascular Disease Risk Accounting for Future Initiation of Statin Treatment

Zhe Xu, Matthew Arnold, David Stevens, Stephen Kaptoge, Lisa Pennells, Michael J. Sweeting, Jessica Barrett, Emanuele Di Angelantonio, and Angela M. Wood

Contents

Web Appendix 1: Code list of cardiovascular disease 1

Web Table 1. Code list of cardiovascular disease in Clinical Practice Research Datalink (CPRD) 1

Web Table 2. Code list of cardiovascular disease in data from Hospital Episode Statistics the Office for National Statistics. 5

Web Appendix 2: Code list of statin prescription 6

Web Table 3. Code list of statin prescription in Clinical Practice Research Datalink (CPRD) 6

Web Appendix 3: Two-stage dynamic landmark age model for risk prediction 9

Web Appendix 4: The closed form calculation of counterfactual survival times in the absence of statin initiation 14

Web Table 4. Summary of metrics used for model predictive performance assessment 17

Web Tables 5-12. Results from the main analyses ....

Web Table 5. Sex-specific incidence rates of 10-year cardiovascular disease by landmark age 22

Web Table 6. Incidence rates of 10-year cardiovascular disease in men 23

Web Table 7. Incidence rates of 10-year cardiovascular disease in women 24

Web Table 8. Hazard ratios of risk predictors for cardiovascular disease in men 25

Web Table 9. Hazard ratios of risk predictors for cardiovascular disease in women 26

Web Table 10. Sex-specific means of the standard 10-year cardiovascular risk predictions versus the statin-naïve 10-year cardiovascular risk prediction 27

Web Table 11. Sex-specific medians and interquartile ranges of the standard 10-year cardiovascular risk predictions versus the statin-naïve 10-year cardiovascular risk predictions 28

Web Table 12. Number needed to screen (NNS) and number needed to treat (NNT) per event prevented 29

Web Tables 13-16. Results from the sensitivity analyses in the validation subset of individuals who remained statin-naïve during follow-up ....

Web Table 13. Overall Brier score and C-index in the subset of individuals who remained statin-naïve during follow-up 30

Web Table 14. Ten-year cardiovascular disease risk classification for men in the subset of individuals who remained statin-naïve during follow-up........................ 31
Web Table 15. Ten-year cardiovascular disease risk classification for women in the subset of individuals who remained statin-naïve during follow-up........................ 33
Web Table 16. Number needed to screen (NNS) and number needed to treat (NNT) per event prevented in the subset of individuals who remained statin-naïve during follow-up.. 35
Web Figures 1-13. Results from the main analyses..
Web Figure 1. Flow chart of study population selection....................................... 36
Web Figure 2: Schematic of landmark age approach 37
Web Figure 3: Sex-specific distribution of the number of measurements of systolic blood pressure, total cholesterol, HDL cholesterol, and smoking status in each landmark age model.. 38
Web Figure 4. Sex-specific survival probablities estimated from Weibull model with no covariates versus Kaplan-Meier survival function in the derivation dataset 39
Web Figure 5. Sex-specific distributions of shape and scale parameters from parametric Weibull models ignoring statin initiation 40
Web Figure 6. Sex-specific distributions of shape and scale parameters from parametric Weibull models accounting for statin initiation 41
Web Figure 7. Venn diagram of the incident cardiovascular events 42
Web Figure 8. Sex-specific medians and interquartile ranges (IQRs) of the statin-naïve 10-year CVD risk predictions versus the standard 10-year CVD risk predictions.. 43
Web Figure 9. Calibration plots for men... 44
Web Figure 10. Calibration plots for women 45
Web Figure 11. Comparison of calibration slopes using models ignoring statin initiation and models accounting for statin initiation 46
Web Figure 12. Comparison of R squared values for models ignoring statin initiation versus models accounting for statin initiation.. 47
Web Figure 13. Comparison of D measures for models ignoring statin initiation versus models accounting for statin initiation.. 48
Web Figures 14-21. Results from the sensitivity analyses in the validation subset of individuals who remained statin-naïve during follow-up ....
Web Figure 14. Calibration plots for men in the subset of individuals who remained statin-naïve during follow-up. 49
Web Figure 15. Calibration plots for women in the subset of individuals who remained statin-naïve during follow-up 50
Web Figure 16. Comparison of calibration slopes in the subset of individuals who remained statin-naïve during follow-up, using models ignoring statin initiation and models accounting for statin initiation 51

Web Figure 17. Comparision of C-indices in the subset of individuals who remained statin-naïve during follow-up, using models ignoring statin initiation versus models accounting for statin initiation 52
Web Figure 18. Comparison of R squared values in the subset of individuals who remained statin-naïve during follow-up, using models ignoring statin initiation versus models accounting for statin initiation 53
Web Figure 19. Comparison of D measures in the subset of individuals who remained statin-naïve during follow-up, using models ignoring statin initiation versus models accounting for statin initiation 54
Web Figure 20. Number needed to screen (NNS) to prevent one cardiovascular disease event and number needed to treat (NNT) to prevent one cardiovascualr disease event in the subset of individuals who remained statin-naïve during follow-up 55
Web Figure 21. Proportion of individuals with 10-year predicted risk exceeding a range of treatment thresholds from 5% to 30% using the statin-naïve versus the standard CVD risk in the subset of individuals who remained statin-naïve during follow-up 56
References 57

Web Appendix 1: Code list of cardiovascular disease

Cardiovascular disease was defined as a combination of newly diagnoses of nonfatal or fatal events of coronary heart disease (CHD) (including myocardial infarction and angina), stroke, and transient ischemic attack (TIA), in line with the definition used in the QRISK3 CVD risk score (1). In Clinical Practice Research Datalink (CPRD), diagnoses are coded using the hierarchical Read code system (1) and in the linked Hospital Episode Statistics and the Office for National Statistics data, the International Classification of Disease 10th revision (ICD-10) codes were used (2).

Web Table 1. Code list of cardiovascular disease in Clinical Practice Research Datalink (CPRD)

Read code	Description
G3...00	Ischaemic heart disease
G31..00	Arteriosclerotic heart disease
G32..00	Atherosclerotic heart disease
G33..00	IHD - Ischaemic heart disease
G30..00	Acute myocardial infarction
G301.00	Attack - heart
G302.00	Coronary thrombosis
G303.00	Cardiac rupture following myocardial infarction (MI)
G304.00	Heart attack
G305.00	MI - acute myocardial infarction
G306.00	Thrombosis - coronary
G307.00	Silent myocardial infarction
G309800	Coronary thrombosis
G309900	Myocardial Infarction
G300.00	Acute anterolateral infarction
G301.00	Other specified anterior myocardial infarction
G301000	Acute anteroapical infarction
G301100	Acute anteroseptal infarction
G301z00	Anterior myocardial infarction NOS
G302.00	Acute inferolateral infarction
G303.00	Acute inferoposterior infarction
G304.00	Posterior myocardial infarction NOS
G305.00	Lateral myocardial infarction NOS
G306.00	True posterior myocardial infarction
G307.00	Acute subendocardial infarction
G307000	Acute non-Q wave infarction
G307100	Acute non-ST segment elevation myocardial infarction
G308.00	Inferior myocardial infarction NOS
G309.00	Acute Q-wave infarct
G30A.00	Mural thrombosis
G30B.00	Acute posterolateral myocardial infarction
G30X.00	Acute transmural myocardial infarction of unspecif site
G30X000	Acute ST segment elevation myocardial infarction
G30y.00	Other acute myocardial infarction
G30y000	Acute atrial infarction
G30y100	Acute papillary muscle infarction

G30y200	Acute septal infarction
G30yz00	Other acute myocardial infarction NOS
G30z.00	Acute myocardial infarction NOS
G31..00	Other acute and subacute ischaemic heart disease
G319900	Acute/subacute IHD NOS
G310.00	Postmyocardial infarction syndrome
G310100	Dressler's syndrome
G311.00	Preinfarction syndrome
G311100	Crescendo angina
G311200	Impending infarction
G311300	Unstable angina
G311400	Angina at rest
G311000	Myocardial infarction aborted
G311010	MI - myocardial infarction aborted
G311100	Unstable angina
G311200	Angina at rest
G311300	Refractory angina
G311400	Worsening angina
G311500	Acute coronary syndrome
G311z00	Preinfarction syndrome NOS
G312.00	Coronary thrombosis not resulting in myocardial infarction
G31y.00	Other acute and subacute ischaemic heart disease
G31y000	Acute coronary insufficiency
G31y099	Acute coronary syndrome
G31y100	Microinfarction of heart
G31y200	Subendocardial ischaemia
G31y300	Transient myocardial ischaemia
G31yz00	Other acute and subacute ischaemic heart disease NOS
G32..00	Old myocardial infarction
G321.00	Healed myocardial infarction
G322.00	Personal history of myocardial infarction
G33..00	Angina pectoris
G330.00	Angina decubitus
G330000	Nocturnal angina
G330z00	Angina decubitus NOS
G331.00	Prinzmetal's angina
G331100	Variant angina pectoris
G332.00	Coronary artery spasm
G33z.00	Angina pectoris NOS
G33z000	Status anginosus
G33z100	Stenocardia
G33z200	Syncope anginosa
G33z300	Angina on effort
G33z400	Ischaemic chest pain
G33z500	Post infarct angina
G33z600	New onset angina
G33z700	Stable angina
G33zz00	Angina pectoris NOS
G34..00	Other chronic ischaemic heart disease
G349900	Chr. ischaemic heart dis. NOS
G340.00	Coronary atherosclerosis
G340100	Triple vessel disease of the heart
G340200	Coronary artery disease

G340000	Single coronary vessel disease
G340100	Double coronary vessel disease
G342.00	Atherosclerotic cardiovascular disease
G343.00	Ischaemic cardiomyopathy
G344.00	Silent myocardial ischaemia
G34y.00	Other specified chronic ischaemic heart disease
G34y000	Chronic coronary insufficiency
G34y100	Chronic myocardial ischaemia
G34yz00	Other specified chronic ischaemic heart disease NOS
G34z.00	Other chronic ischaemic heart disease NOS
G34z000	Asymptomatic coronary heart disease
G35..00	Subsequent myocardial infarction
G350.00	Subsequent myocardial infarction of anterior wall
G351.00	Subsequent myocardial infarction of inferior wall
G353.00	Subsequent myocardial infarction of other sites
G35X.00	Subsequent myocardial infarction of unspecified site
G36..00	Certain current complication follow acute myocardial infarct
G360.00	Haemopericardium/current comp follow acute myocardial infarct
G361.00	Atrial septal defect/curr comp follow acute myocardial infarct
G362.00	Ventricular septal defect/curr comp follow acute myocardial infarction
G363.00	Ruptur cardiac wall w'out haemopericard/cur comp follow ac MI
G364.00	Ruptur chordae tendinae/curr comp follow acute myocardial infarct
G365.00	Rupture papillary muscle/curr comp follow acute myocardial infarct
G366.00	Thrombosis atrium, auric append&vent/curr comp follow acute MI
G38..00	Postoperative myocardial infarction
G380.00	Postoperative transmural myocardial infarction anterior wall
G381.00	Postoperative transmural myocardial infarction inferior wall
G382.00	Postoperative transmural myocardial infarction other sites
G383.00	Postoperative transmural myocardial infarction unspec site
G384.00	Postoperative subendocardial myocardial infarction
G38z.00	Postoperative myocardial infarction, unspecified
G3y..00	Other specified ischaemic heart disease
G3z..00	Ischaemic heart disease NOS
G501.00	Post infarction pericarditis
Gyu3400	[X]Acute transmural myocardial infarction of unspecif site
F423600	Amaurosis fugax
Fyu5500	[X]Other transnt cerebral ischaemic attacks+related syndromes
G63y000	Cerebral infarct due to thrombosis of precerebral arteries
G63y100	Cerebral infarction due to embolism of precerebral arteries
G64..00	Cerebral arterial occlusion
G641.00	CVA - cerebral artery occlusion
G642.00	Infarction - cerebral
G643.00	Stroke due to cerebral arterial occlusion
G640.00	Cerebral thrombosis
G640000	Cerebral infarction due to thrombosis of cerebral arteries
G641.00	Cerebral embolism
G641100	Cerebral embolus
G641000	Cerebral infarction due to embolism of cerebral arteries
G64z.00	Cerebral infarction NOS
G64z100	Brainstem infarction NOS
G64z200	Cerebellar infarction
G64z990	Cerebral A. occlusion NOS
G64z000	Brainstem infarction

G64z100	Wallenberg syndrome
G64z110	Lateral medullary syndrome
G64z200	Left sided cerebral infarction
G64z300	Right sided cerebral infarction
G64z400	Infarction of basal ganglia
G65..00	Transient cerebral ischaemia
G651.00	Drop attack
G652.00	Transient ischaemic attack
G653.00	Vertebro-basilar insufficiency
G659900	Transient Ischaemic Attacks
G650.00	Basilar artery syndrome
G650100	Insufficiency - basilar artery
G652.00	Subclavian steal syndrome
G653.00	Carotid artery syndrome hemispheric
G654.00	Multiple and bilateral precerebral artery syndromes
G656.00	Vertebrobasilar insufficiency
G65y.00	Other transient cerebral ischaemia
G65z.00	Transient cerebral ischaemia NOS
G65z990	Transient Ischaemic Attacks
G65z000	Impending cerebral ischaemia
G65z100	Intermittent cerebral ischaemia
G65zz00	Transient cerebral ischaemia NOS
G66..00	Stroke and cerebrovascular accident unspecified
G661.00	CVA unspecified
G662.00	Stroke unspecified
G663.00	CVA - Cerebrovascular accident unspecified
G669800	Stroke/CVA - undefined
G669900	Stroke
G667.00	Left sided CVA
G668.00	Right sided CVA
G676000	Cereb infarct due cerebral venous thrombosis, nonpyogenic
G6W..00	Cereb infarct due unspcf occlus/stenos precerebr arteries
G6X..00	Cerebrl infarctn due/unspcf occlusn or sten/cerebrl artrs
Gyu6300	[X]Cerebrl infarctn due/unspcf occlusn or sten/cerebrl artrs
Gyu6400	[X]Other cerebral infarction
Gyu6500	[X]Occlusion and stenosis of other precerebral arteries
Gyu6600	[X]Occlusion and stenosis of other cerebral arteries
ZV12D00	[V]Personal history of transient ischaemic attack

Web Table 2. Code list of cardiovascular disease in data from Hospital Episode Statistics the Office for National Statistics.

ICD10-code	description
G45	transient ischaemic attack and related syndromes
G45.0	transient ischaemic attack and related syndromes
G45.1	transient ischaemic attack and related syndromes
G45.2	transient ischaemic attack and related syndromes
G45.3	transient ischaemic attack and related syndromes
G45.4	transient ischaemic attack and related syndromes
G45.8	transient ischaemic attack and related syndromes
G45.9	transient ischaemic attack and related syndromes
I20	angina pectoris
I20.0	angina pectoris
I20.1	angina pectoris
I20.8	angina pectoris
I20.9	angina pectoris
I21	acute myocardial infarction
I21.0	acute myocardial infarction
I21.1	acute myocardial infarction
I21.2	acute myocardial infarction
I21.3	acute myocardial infarction
I21.4	acute myocardial infarction
I21.9	acute myocardial infarction
I22	subsequent myocardial infarction
I22.0	subsequent myocardial infarction
I22.1	subsequent myocardial infarction
I22.8	subsequent myocardial infarction
I22.9	subsequent myocardial infarction
I23	complications after myocardial infarction
I23.0	complications after myocardial infarction
I23.1	complications after myocardial infarction
I23.2	complications after myocardial infarction
I23.3	complications after myocardial infarction
I23.4	complications after myocardial infarction
I23.5	complications after myocardial infarction
I23.6	complications after myocardial infarction
I23.8	complications after myocardial infarction
I24	other acute ischaemic heart disease
I24.0	other acute ischaemic heart disease
I24.1	other acute ischaemic heart disease
I24.8	other acute ischaemic heart disease
I24.9	other acute ischaemic heart disease
I25	chronic ischaemic heart disease
I25.0	chronic ischaemic heart disease

I25.1	chronic ischaemic heart disease
I25.2	chronic ischaemic heart disease
I25.3	chronic ischaemic heart disease
I25.4	chronic ischaemic heart disease
I25.5	chronic ischaemic heart disease
I25.6	chronic ischaemic heart disease
I25.8	chronic ischaemic heart disease
I25.9	chronic ischaemic heart disease
I63	cerebral infarction
I63.0	cerebral infarction
I63.1	cerebral infarction
I63.2	cerebral infarction
I63.3	cerebral infarction
I63.4	cerebral infarction
I63.5	cerebral infarction
I63.6	cerebral infarction
I63.8	cerebral infarction
I63.9	cerebral infarction
I64	stroke not specified as haemorrhage or infarction

Web Appendix 2: Code list of statin prescription in Clinical Practice Research Datalink (CPRD)

In CPRD, prescriptions issued by the GP are automatically recorded with a product name and British National Formulary code, with the dosage instructions and quantity (3). We defined statin initiation as the date of first CPRD prescription. Information about statin prescription was extracted using the following CPRD product code list in Web Table 3.

Web Table 3. Code list of statin prescription in Clinical Practice Research Datalink (CPRD)

CPRD_product_code	description
10172	ezetimibe with simvastatin tablets 10mg + 40mg
10183	simvastatin with ezetimibe tablets 40mg + 10mg
10206	simvastatin with ezetimibe tablets 80mg + 10mg
11627	fluvastatin 24 hour modified release tablets 80mg
11815	simvastatin with ezetimibe tablets 20mg + 10mg
1219	pravastatin tablets 40mg
1221	LIPOSTAT tablets 10mg [SQUIBB]
1223	LIPOSTAT tablets 40mg [SQUIBB]
13041	SIMVADOR tablets 10mg [DISCOVERY]

14219	ezetimibe with simvastatin tablets 10mg + 80mg
15252	CRESTOR tablets 20mg [ASTRAZENEC]
16186	INEGY tablets 10mg + 80mg [M S D]
17059	INEGY tablets 10mg + 40mg [M S D]
17683	LIPITOR tablets 80mg [PFIZER]
17688	CRESTOR tablets 5mg [ASTRAZENEC]
18442	LIPOBAY tablets 400micrograms [BAYER]
21020	INEGY tablets 10mg + 20mg [M S D]
2137	fluvastatin capsules 40mg
22579	ZOCOR tablets 80mg [M S D]
25	simvastatin tablets 20mg
2718	ZOCOR tablets 10mg [M S D]
28	atorvastatin tablets 10mg
2955	LIPITOR tablets 40mg [PFIZER]
31658	cerivastatin tablets 800micrograms
31930	ZOCOR HEART-PRO tablets 10mg [MCNEIL]
32909	SIMVASTATIN tablets 80mg [HILLCROSS]
32921	PRAVASTATIN tablets 10mg [DR REDDY`S]
33082	SIMVASTATIN tablets 20mg [HILLCROSS]
3411	LIPITOR tablets 10mg [PFIZER]
34312	SIMVASTATIN tablets 20mg [GEN (UK)]
34316	SIMVASTATIN tablets 20mg [TEVA]
34353	SIMVASTATIN tablets 40mg [GEN (UK)]
34366	SIMVASTATIN tablets 20mg [IVAX]
34376	SIMVASTATIN tablets 40mg [TEVA]
34381	SIMVASTATIN tablets 40mg [IVAX]
34476	SIMVASTATIN tablets 20mg [RATIOPHARM]
34481	SIMVASTATIN tablets 10mg [IVAX]
34502	SIMVASTATIN tablets 40mg [HILLCROSS]
34535	SIMVASTATIN tablets 10mg [GEN (UK)]
34545	SIMVASTATIN tablets 40mg [RATIOPHARM]
34560	SIMVASTATIN tablets 10mg [RATIOPHARM]
34746	SIMVASTATIN tablets 20mg [NICHE]
34814	SIMVASTATIN tablets 20mg [WOCKHARDT]
34820	PRAVASTATIN tablets 40mg [HILLCROSS]
34879	SIMVASTATIN tablets 40mg [NICHE]
34891	SIMVASTATIN tablets 20mg [KENT]
34907	SIMVASTATIN tablets 40mg [WOCKHARDT]
34955	SIMVASTATIN tablets 10mg [HILLCROSS]
34969	SIMVASTATIN tablets 40mg [ACTAVIS]
36377	PRAVASTATIN tablets 20mg [TEVA]
3690	LIPOSTAT tablets 20mg [SQUIBB]
37434	SIMVASTATIN tablets 40mg [SANDOZ]
379	fluvastatin capsules 20mg
39060	SIMVASTATIN tablets 20mg [DEXCEL]
39652	simvastatin sugar free oral suspension 40mg/5ml

39675	SIMVASTATIN oral suspension 20mg/5ml [MARTINDALE]
39870	SIMVADOR tablets 80mg [DISCOVERY]
40340	SIMVASTATIN tablets 10mg [TEVA]
40382	PRAVASTATIN tablets 20mg [HILLCROSS]
40601	SIMVASTATIN tablets 20mg [RANBAXY]
41657	SIMVASTATIN tablets 80mg [TEVA]
42	simvastatin tablets 10mg
420	cerivastatin tablets 100micrograms
43218	PRAVASTATIN tablets 10mg [TEVA]
44528	SIMVASTATIN sugar free oral suspension 20mg/5ml [ROSEMONT]
44650	SIMVASTATIN tablets 40mg [DEXCEL]
44878	RANZOLONT tablets 10mg [RANBAXY]
45219	SIMVASTATIN tablets 40mg [KENT]
45235	SIMVASTATIN tablets 20mg [SANDOZ]
45245	SIMVASTATIN tablets 20mg [ACTAVIS]
45346	SIMVASTATIN tablets 40mg [ARROW]
46878	SIMVASTATIN tablets 40mg [ALMUS]
46956	SIMVASTATIN tablets 80mg [ARROW]
47065	atorvastatin chewable tablet 20mg
47090	atorvastatin chewable tablet 10mg
490	pravastatin tablets 10mg
4961	LIPOBAY tablets 300micrograms [BAYER]
5009	cerivastatin tablets 200micrograms
51	simvastatin tablets 40mg
5148	simvastatin tablets 80mg
5251	cerivastatin tablets 300micrograms
5278	cerivastatin tablets 400micrograms
5775	atorvastatin tablets 80mg
5985	LESCOL XL tablets 80mg [NOVARTIS]
6168	ZOCOR tablets 40mg [M S D]
6213	rosuvastatin tablets 20mg
713	rosuvastatin tablets 10mg
7196	ZOCOR tablets 20mg [M S D]
730	pravastatin tablets 20mg
7347	CRESTOR tablets 10mg [ASTRAZENEC]
7374	LIPITOR tablets 20mg [PFIZER]
745	atorvastatin tablets 40mg
75	atorvastatin tablets 20mg
7552	ezetimibe with simvastatin tablets 10mg + 20mg
7554	rosuvastatin tablets 5mg
802	SIMVADOR tablets 40mg [DISCOVERY]
818	simvastatin sugar free oral suspension 20mg/5ml
8380	LESCOL capsules 20mg [NOV/SANDOZ]
9153	LESCOL capsules 40mg [NOV/SANDOZ]
9315	LIPOBAY tablets 100micrograms [BAYER]

9316	LIPOBAY tablets 200micrograms [BAYER]
9897	rosuvastatin tablets 40mg
9920	SIMVADOR tablets 20mg [DISCOVERY]
9930	CRESTOR tablets 40mg [ASTRAZENEC]

Web Appendix 3: Two-stage dynamic landmark age model for risk prediction

Dynamic landmark age modelling

To optimize the use of repeated measurements of risk factors in electronic health records to predict future CVD risk, we used sliding landmark approach as described in our previous study (4) to construct 10-year CVD risk prediction models. The schematic of landmark age approach is presented in Web Figure 2. A landmark age is a reference point at which we use risk factor values collected prior to that age and from which to predict future risk (4). In the derivation dataset, we derived a series of ninety two age- and sex-specific predictions models (i.e., for men and women and at ages 40, 41, 42, …,85, denoted as “landmark ages”). Participants contributed to the models if they have 1) registered with a general practice at the landmark age, 2) no CVD diagnoses prior to the landmark age, and 3) no statin prescription prior to the landmark age.

We selected the key cardiovascular risk factors as those used in the validated 2013 ACC/AHA Pooled Cohort Equations (5): age, sex, total cholesterol, high-density lipoprotein (HDL) cholesterol, systolic blood pressure (SBP), use of antihypertensive therapy, diabetes mellitus status, and smoking status. Values of systolic blood pressure, total cholesterol and HDL cholesterol were standardized by centering on sex- specific means and dividing by the standard deviation (using means and standard deviations calculated from the first measurement from each individual). Age and sex were known for all participants. Values for diabetes mellitus status, anti-hypertensive therapy usage and statin therapy usage were set to zero until the first available health record indicated otherwise (i.e. for diabetes mellitus: at least one diabetes diagnostic code [Read code or diabetes test] plus either an additional diagnostic code or diabetes drug prescription (6); first prescription of a blood-pressure or cholesterol medication) from which time the values were set to one. Repeat measurements of smoking status, systolic blood pressure, total cholesterol and HDL cholesterol were first summarised

using age- and sex-specific multivariate mixed models (7) and entered the prediction model as single summary measures as described below.

The landmark age approach comprises of two stages:

Stage 1: *Summarising repeated measures of risk factors using multivariate mixed-effects linear regression models*

Let $Smoking\_status_{ij}$, SBP_{ij}, $Total\_cholesterol_{ij}$, $HDL\_cholesterol_{ij}$, BP_med_{ij} and $Statin_{ij}$ denote all the repeat measurements of smoking status, systolic blood pressure, total cholesterol, HDL cholesterol, indication of blood pressure-lowering medication, and indication of statin initiation for individual i recorded at measurement j. For males and females separately, for each landmark age La = 40, 41, 42, ...,85, we fit a multivariate mixed-effect model with a correlated covariance structure:

$$SBP_{ij} = \alpha_1 + \beta_1 * t_{ij} + \gamma * BP\_med_{ij} + u_{1i} + \varepsilon_{1ij}$$

$$Total\_cholesterol_{ij} = \alpha_2 + \beta_2 * t_{ij} + \delta * Statin_{ij} + u_{2i} + \varepsilon_{2ij}$$

$$HDL\_cholesterol_{ij} = \alpha_3 + \beta_3 * t_{ij} + u_{3i} + \varepsilon_{3ij}$$

$$Smoking\_status_{ij} = \alpha_4 + \beta_4 * t_{ij} + u_{4i} + \varepsilon_{4ij}$$

where $\begin{bmatrix} u_{1i} \\ u_{2i} \\ u_{3i} \\ u_{4i} \end{bmatrix} \sim multivariate\ normal \left(\begin{bmatrix} 0 \\ 0 \\ 0 \\ 0 \end{bmatrix}, \begin{bmatrix} \sigma_1^2 & \sigma_{12} & \sigma_{13} & \sigma_{14} \\ \sigma_{12} & \sigma_2^2 & \sigma_{23} & \sigma_{24} \\ \sigma_{13} & \sigma_{23} & \sigma_3^2 & \sigma_{34} \\ \sigma_{14} & \sigma_{24} & \sigma_{34} & \sigma_4^2 \end{bmatrix} \right)$,

and $\begin{bmatrix} \varepsilon_{1i} \\ \varepsilon_{2i} \\ \varepsilon_{3i} \\ \varepsilon_{4i} \end{bmatrix} \sim multivariate\ normal \left(\begin{bmatrix} 0 \\ 0 \\ 0 \\ 0 \end{bmatrix}, \begin{bmatrix} \sigma_{e1}^2 & 0 & 0 & 0 \\ 0 & \sigma_{e2}^2 & 0 & 0 \\ 0 & 0 & \sigma_{e3}^2 & 0 \\ 0 & 0 & 0 & \sigma_{e4}^2 \end{bmatrix} \right)$

Here α_1, α_2, α_3 and α_4 represent fixed intercepts for each risk factor, β_1, β_2, β_3 and β_4 represent fixed slopes for each risk factor, γ represents an adjustment factor in systolic blood pressure levels for those with an indication of blood pressure-lowering medication and δ represents an adjustment factor in total cholesterol for those with an indication of statin medication.

Terms u_{1i}, u_{2i}, u_{3i} and u_{4i} represent random intercepts for each risk factor and are correlated between risk factors. These random intercepts are interpreted as the difference in the average level of the predictor for this individual compared to the population average level.

Finally, ε_{1ij}, ε_{2ij}, ε_{3ij} and ε_{4ij} represent uncorrelated residual errors for each risk factor.

This model allows incomplete records of the risk factors and includes all individuals with at least one measurement from at least one risk factor (see Web Figure 3).The correlation structure between the risk factors is estimated from individuals with observed data on more than one risk factor. Thus, the model assumes that, for each landmark age, risk factor values from individuals with incomplete data are from the same multivariate normal distribution for risk factor values for individuals with observed data (that is, assuming "missing at random").

Our model assumes that all risk factors jointly follow a multivariate normal distribution, which is plausible for SBP, total cholesterol, HDL cholesterol but less plausible for smoking status which is defined as a binary variable (yes for current/ever smoker; no for never smoker). However, inference based from the multivariate normal distribution may often be reasonable even if the multivariate normality does not hold, especially in the context of imputation of missing data (8) and regression calibration (9,10).

In our previous work (4), we restricted the model derivation to repeat measurements recorded **before** the landmark age, i.e. $j \leq La$. However, we found slight improvements in sensitivity analyses when we used all available repeated measurements recorded **before** and **after** the landmark age, due to extra precision on parameter estimates. We accept a limitation is that it ignores informative censoring of individuals due to death or CVD events, however, our previous work shows informative censoring has little effect on the long-term usual levels of the included risk factors.

Best linear unbiased predictors (BLUPS) (11) are estimated for each risk factor for the random intercepts $\hat{u}_{1i}$, $\hat{u}_{2i}$, $\hat{u}_{3i}$ and $\hat{u}_{4i}$ using observed data for $j \leq La$ (12). Note the restriction to only repeat measurements before the landmark age is important here, as

the prediction model is intended for use in clinical practice where only past data will be available. The BLUPs are estimated as the mean of the empirical Bayes posterior distribution of the random intercepts conditional on observed risk factor measurements. Using the properties of multivariate normal distributions, this is also a multivariate normal distribution, and an exact formula for the mean can be calculated (13).

Specifically, for individual i

$$\begin{bmatrix} \hat{u}_{1i} \\ \hat{u}_{2i} \\ \hat{u}_{3i} \\ \hat{u}_{4i} \end{bmatrix} = GZ^T(ZGZ^T + \sum)^{-1}(Y - X\beta)$$

Here Y is the vector of risk factor observations, G is the covariance matrix of the random effects = $\begin{bmatrix} \sigma_1^2 & \sigma_{12} & \sigma_{13} & \sigma_{14} \\ \sigma_{12} & \sigma_2^2 & \sigma_{23} & \sigma_{24} \\ \sigma_{13} & \sigma_{23} & \sigma_3^2 & \sigma_{34} \\ \sigma_{14} & \sigma_{24} & \sigma_{34} & \sigma_4^2 \end{bmatrix}$, Z is the design matrix which selects the corresponding random effect for each risk factor, Z^T is the matrix transpose of Z and $\sum$ is a diagonal matrix containing the corresponding residual variance for each risk factor. Importantly, due to the correlations structure between the random intercepts, BLUPS can be estimated for all individuals with at least one repeat measurement for at least one risk factor.

Stage 2: *Estimating 10-year CVD risk using landmark age- and sex-specific Weibull proportional hazards models, accounting for future statin initiation effect.*

In the second stage, ten-year CVD risk was modelled using landmark age- and sex-specific Weibull models, with time since landmark age as the time scale. Landmark age datasets were constructed comprising of participants with no CVD diagnoses and/or statin prescription prior to that landmark age and included the following variables: (i) landmark-age-dependent outcome time-to-CVD-event and censoring indicator; (ii) sex; (iii) landmark- age-dependent estimated error-free risk factor values for SBP, total cholesterol, HDL cholesterol, smoking status and the most recent observed records for diabetes status and history of blood pressure-lowering medication prescriptions, denoted together as $X(La)$ and (iv) landmark-age-

dependent time-to-statin-initiation during follow-up (set to the “time-to-CVD-event” if not observed) and statin-initiation indicator. We then split the time-to-CVD-event records at the time-to-statin-initiation, so that individuals who had an indication of statin initiation during follow-up had two records, one covering the landmark age before statin initiation, and the second from statin initiation to the CVD event or censoring. To each landmark age data set, we fit the following sex-stratified Weibull model:

$$h_s(t|X(La), La) = h_{0s}(t)\exp[{\beta_x}^T X(La) + B \times Statin(t)]$$

where $h_{0s}(t) = \lambda \nu t^{\nu-1}$, with scale and shape parameters λ and ν, and the scale parameter λ is parameterized as $\exp(\beta_0)$; $Statin(t)$ is the time dependent indicator which equals 0 before an indication of statin-initiation, and equals 1 at and after the first indication of statin-initiation; and B represents the effect of statin-initiation on the risk of CVD, which is constrained to $B = \ln(0.75)$ to represent a 25% risk reduction as reported from published meta-analyses of trials. (14,15) This is done by using *offset* option in the Weibull survival model in Stata. The code sample for each landmark age by gender for estimating 10-year CVD risk accounting for statin-initiation is as follows:

```
stset ft, failure (cvd_ind ==1) id(patid)
stsplit new_statin_ind, after(time=statin_time) at(0)
replace new_statin_ind=new_statin_ind+1
*convert -1, 0 to 0,1 for on statins
replace new_statin_ind=0 if statins_prscd==. |
(statins_prscd!=. & statins_prscd>=exit_date)
*for never statin before exit_date
gen beta_x=new_statin_ind*ln(0.75)
streg sbp bp_medication tchol hdl smoke diabetes if
derivation==1, offset(beta_x) dist(weibull)
```

where `ft` is follow-up time; `cvd_ind` is the incident CVD indicator; `statin_time` is the time-to-statin-initiation during follow-up (set to the “time-to-CVD-event” if not observed); `new_statin_ind` is the time-varying indicator for statin initiation (1 for on statins, 0 for no statins); `ln(0.75)` is the 25% risk reduction as reported from published meta-analyses of trials; *sbp, bp_medication, tchol, hdl, smoke, diabetes* are the risk factor values estimated from Stage 1.

Predicted 10-year CVD risk is estimated for participants at each landmark age from the equation:

$$1 - P\big(T > La + 10 \big| T > La, X(La)\big) = 1 - S_{0s}(La + 10|La)^{\exp\left[\beta_x{}^T X(La) + B \times Statin(t)\right]}$$

where $S_{0s}(La + 10|La) = \exp\,(-\lambda t^{\nu})$ represents the sex-stratified 10-year baseline survival from landmark age La.

Other survival models, including the non-parametric Cox model, and more flexible parametric forms, could be used in place of the Weibull model. We selected the Weibull model due to a reasonable fit, and to enable a closed form solution to the calculation of counterfactual survival times in the absence of statin initiation (see Appendix 3). In our analysis, the fitted survival probability curves from the Weibull models were consistent with the Kaplan—Meier curves, indicating a reasonable fit (Web Figure 4). The fitted Weibull model shape parameters ranged between 1.07 to 1.34 across landmark ages (Web Figures 5 and 6) the Weibull model was more sufficient than exponential model of which the shape parameter is defined as 1.

Proportional hazard assumption in Weibull model:

The Weibull hazard function is $h_0(t) = \lambda\nu t^{\nu-1}$ where the scale parameter λ is parametrized as $\exp(\beta_0)$. The hazard ratio for statin treatment is obtained as $HR = \frac{\exp(\beta_0 + \beta_s)\nu t^{\nu-1}}{\exp(\beta_0)\nu t^{\nu-1}} = \exp(\beta_s)$, which is the statin treatment effect. This result depends on the shape parameter ν having the same value for treatment vs. non-treatment to be cancelled out to get HR, and so that the proportional hazard assumption is satisfied. In our analysis, the proportional hazard assumption was satisfied since the shape parameters are generally same for models ignoring vs. accounting for statin effect (Web Figures 5 and 6).

Web Appendix 4: The closed form calculation of counterfactual survival times in the absence of statin initiation

The assessment of model calibration and discrimination using information only known at the landmark age (i.e., no knowledge of future statin-initiation) requires "counterfactual observed" survival times in the absence of statin initiation, otherwise

our models would over-estimate the observed risk of CVD in individuals who went on to initiate statins. Counterfactual survival times were estimated as follows.

Denote t as the observed time-to-CVD-event (or censoring time), let t_s be the time-to-statin-initiation (or equal to t if not observed) and let t^* be the counterfactual statin-naïve time-to-CVD-event.

The cumulative hazard function for t, using the Weibull model can be written as:

$$H(t) = \int_0^t h_{0s}(u)\exp[{\beta_x}^T X(La) \ + B \times Statin(u)\ ]\, du$$

$$= \int_0^{t_s} h_{0s}(u)\exp\left({\beta_x}^T X(La)\right)du + \int_{t_s}^{t} h_{0s}(u)\exp[{\beta_x}^T X(La) + B]du$$

$$= \ \exp\left({\beta_x}^T X(La)\right) \times \left[\int_0^{t_s} h_{0s}(u)du + \exp(B)\int_{t_s}^{t} h_{0s}(u)\ du\right]$$

$$= \exp\left({\beta_x}^T X(La)\right) \times \{H_0(t_s) + \exp(B) \times \ [H_0(t) - H_0(t_s)]\}.$$

The cumulative hazard function for t*, using the Weibull model can be written as:

$$H(t^*) = \int_0^{t^*} h_{0s}(u)\exp\,({\beta_x}^T X(La))\, du = \ \exp\,({\beta_x}^T X(La)) \times H_0(t^*)$$

Under the proportional-hazards assumption for the effect of statins, we assume equality of the cumulative hazard function for t and t^*, because the model with observed population and observed follow-up time with additionally adjusted for time-dependent statin use should be equal to the estimation from the counterfactual population with no one had statins using the counterfactual time Consequently,

$$\exp\,({\beta_x}^T X(La)) \times H_0(t^*) = \exp\left({\beta_x}^T X(La)\right) \times \{H_0(t_s) + \exp(B) \times \ [H_0(t) - H_0(t_s)]\}$$

$$H_0(t^*) = \ \{H_0(t_s) + \exp(B) \times \ [H_0(t) - H_0(t_s)]\}$$

$$\lambda t^{*\nu} = \lambda {t_s}^{\nu} + \exp(B) \times (\lambda t^{\nu} - \lambda {t_s}^{\nu})$$

$$t^* = [{t_s}^{\nu} + \ \exp(B) \times (t^{\nu} - {t_s}^{\nu})]^{1/\nu}.$$

When $\nu = 1$, (i.e., the exponential model) and assuming a 25% reduction in risk by statins, then intuitively, $t^* = t_s + \ 0.75 * (t - t_s)$.

It is not possible to formulate a closed form calculation for $t^*$for the Cox model, however, the cumulative hazard $H_0(t^*)$ can be estimated, from which an estimate of

t^* could be approximated from the observed times t, either assuming step functions between observed event times (fine in large datasets with many unique observed event times) or after applying some smoothing function.

Web Table 4. Summary of metrics used for model predictive performance assessment

Aspect	Metric	Description	Data	Use of follow-up times for model accounting for and ignoring statin initiation[a]
Overall performance	Brier score	Squared differences between observed 10-year CVD outcomes and predicted risk (16).<br><br>Lower values indicate better accuracy.	Only information from individuals with at least 10 years of follow-up or with a CVD event within 10-years.	Use t* to define the counterfactual 10-year CVD status to assess models accounting for statin initiation<br><br>Use t to define 10-year CVD status to assess models ignoring statin initiation
Overall performance	R^2 values based on Royston & Sauerbrei's D statistic	Percentage of variation in time-to-CVD-event outcomes explained by the prediction model (16–18).<br><br>Higher values indicate better performance.	All data	Use t* to assess the models accounting for statin initiation.<br><br>Use t to assess the models ignoring statin initiation.
Discrimination	Harrell's C-index	A rank-order measure to quantify the discriminative ability to rank individuals according to their predicted and observed risk of CVD (19,20).<br><br>A C-index value of 1 means perfect discrimination, and value of 0.5 means by chance alone.	All data	Use t* to assess the models accounting for statin initiation.<br><br>Use t to assess the models ignoring statin initiation.
Discrimination	D statistic	Log hazard ratio comparing the two equal-sized prognostic groups (18).	All data	Use t* to assess the models accounting for statin initiation.

		Higher values indicate better discrimination.		Use t to assess the models ignoring statin initiation.
Calibration	Calibration plot	Displays the agreement between observed outcomes and predicted risk (21).<br>Plots compare the mean predicted 10-year risk vs. the observed 10-year CVD outcomes by deciles of predicted risk.<br><br>The observed outcomes were obtained from the Kaplan-Meier estimates close to 10 years (16,20).	All data	Use t* to calculate mean observed risks to assess calibration for the statin-naïve 10-year CVD risk predictions.<br><br>Use t to calculate mean observed risks to assess calibration for the standard 10-year CVD risk predictions.
Calibration	Calibration slope	The regression slope coefficient from the linear regression $\ln(-\ln(1-Y_{obs})) = \beta_0 + \beta_1 * \ln(-\ln(1-Y_{pred}))$, of the mean observed risk (Y_{obs}) on mean predicted risk (Y_{pred}), where the mean risks are calculated by deciles of predicted risk (16,20,22,23).<br><br>Values close to 1 indicate better calibration.	All data	Use t* to calculate mean observed risks to assess calibration for the statin-naïve 10-year CVD risk predictions.<br><br>Use t to calculate mean observed risks to assess calibration for the standard 10-year CVD risk predictions.
Calibration	Goodness-of-fit assessment	Goodness-of-fit statistic for the observed 10-year CVD outcomes and predicted 10-year risk by decile of predicted risk, using a χ^2 statistic (24).<br><br>Low p-values indicate poor model fit.	All data	Use t* to calculate mean observed risks to assess calibration for the statin-naïve 10-year CVD risk predictions.<br><br>Use t to calculate mean observed risks to assess

				calibration for the standard 10-year CVD risk predictions.
Reclassification	Retrospective form of the net reclassification improvement (NRI)	Quantifies the movement of individuals between predefined risk categories of <10% and ≥10%, which is the threshold of recommended statin treatment in the current UK guidelines (25)). Calculates the difference in the following two quantities: (i) the difference between the proportions of individuals moving up and the proportion of individuals moving down for those who develop CVD events and (ii) the corresponding difference in proportions for those who not develop CVD (26).	Only information from individuals with at least 10 years of follow-up or with a CVD event within 10-years.	Use t* to define the counterfactual 10-year CVD status to assess models.
	Prospective form of NRI (continuous NRI)	The category-free version of NRI which can include individuals with censored events. It is a measure of the event rate increase among those who are reclassified upwards and the event rate decrease among those who are reclassified downwards (27).	All data	The expected proportions of reclassified individuals are estimated using the Kaplan-Meier approach.<br><br>Use t* to calculate the counterfactual Kaplan-Meier estimates.
	Integrated discrimination improvement (IDI)	Integrates the NRI over all possible cut-offs of predicted risk and mathematically equivalent to difference in the means of predicted probability of the outcome calculated from different models (20,28).	Only information from individuals with at least 10 years of follow-up or with a CVD event within 10-years.	Use t* to define the counterfactual 10-year CVD status to assess models.

Clinical performance	Number needed to screen (NNS) to prevent one event (23,29,30)	NNS = number of screened people / number of extra expected events prevented by statins<br><br>Assuming that statin treatment is associated with a 25% relative risk reduction of CVD and that people who exceed a high risk threshold (e.g. 10% 10-year CVD risk) would start statin therapy, then CVD risk assessment would be expected to prevent (25% * number of individuals who had CVD over the next 10 years (i.e., the cases) and exceeded statin treatment threshold) events.<br><br>Thus, NNS = [n(cases) + n(non-cases)]/[n(high risk people among cases)*0.25], where lower numbers suggest better efficiency of the model.	Only information from individuals with at least 10 years of follow-up or with a CVD event within 10-years.	Use t* to define the counterfactual 10-year CVD status to assess models.
	Number needed to treat (NNT) to prevent one event (23,29,30)	NNT = number of expected treated people / number of extra expected events prevented by statins<br>= [n(high risk people among case)+ n(high risk people among non-case)]/[n(high risk people among cases)*0.25], where lower numbers suggest better efficiency of the model.	Only information from individuals with at least 10 years of follow-up or with a CVD event within 10-years.	Use t* to define the counterfactual 10-year CVD status to assess models.
	The proportion of individuals with 10-year predicted risk	the proportion of individuals with 10-year predicted risk exceeding a range of treatment thresholds from 5% to 30%	All data	Follow-up time was not used for the proportion calculation

	exceeding treatment threshold	using the statin-naïve versus the standard CVD risk for each landmark age. Weighted proportion across all ages were calculated using the most recent available data for an age-sex standard England population in 2015 (31) between 40-85 years.		

[a] **t** represents the observed follow-up time; **t*** represents the counterfactual follow-up time in the absence of statin initiation

Web Table 5. Sex-specific incidence rates and 95% confidence intervals of 10-year cardiovascular disease by landmark age, Clinical Practice Research Datalink, Hospital Episode Statistics, and the Office for National Statistics, England, United Kingdom, 2004-2017 (in 5-year increments in landmark age for presentation)

Men					
**Landmark age**	**No. of persons in risk set**	**No. of events in risk set**	**Total PY**	**Incident rate per 1000 PY**	**95% CI**
40	126,059	1,444	525,964	2.75	2.61, 2.89
45	146,613	2,810	612,048	4.59	4.42, 4.76
50	144,731	4,266	592,992	7.19	6.98, 7.41
55	121,546	5,276	504,798	10.45	10.17, 10.74
60	106,610	6,347	463,107	13.71	13.37, 14.05
65	82,258	6,173	340,368	18.14	17.69, 18.59
70	56,123	5,907	232,107	25.45	24.81, 26.11
75	38,393	5,586	158,207	35.31	34.39, 36.25
80	29,395	5,080	109,332	46.46	45.20, 47.76
85	18,603	3,500	57,331	61.05	59.06, 63.11
**Women**					
**Landmark age**	**No. of persons in risk set**	**No. of events in risk set**	**Total PY**	**Incident rate per 1000 PY**	**95% CI**
40	178,659	1,063	796,927	1.33	1.26, 1.42
45	186,696	1,811	818,297	2.21	2.11, 2.32
50	177,336	2,534	758,369	3.34	3.21, 3.47
55	150,541	3,046	654,043	4.66	4.49, 4.83
60	134,540	4,064	607,075	6.69	6.49, 6.90
65	108,884	4,577	468,776	9.76	9.48, 10.05
70	79,352	5,281	339,013	15.58	15.16, 16.00
75	58,948	6,038	255,145	23.67	23.08, 24.27
80	47,632	6,569	188,764	34.80	33.97, 35.65
85	35,781	5,839	120,109	48.61	47.38, 49.88

Abbreviations: CI, confidence interval; PY, person-years

Web Table 6. Incidence rates and 95% confidence intervals of 10-year cardiovascular disease by landmark age in men in the derivation and validation datasets, Clinical Practice Research Datalink, Hospital Episode Statistics, and the Office for National Statistics, England, United Kingdom, 2004-2017 (in 5-year increments in landmark age for presentation)

Landmark age	No. of persons in risk set	No. of events in risk set	Total PY	Incident rate per 1000 PY	95% CI
**Derivation dataset**					
40	85,693	1,012	360,822	2.80	2.64, 2.98
45	100,402	1,888	421,249	4.48	4.28, 4.69
50	99,196	2,974	408,723	7.28	7.02, 7.54
55	83,122	3,658	345,983	10.57	10.24, 10.92
60	72,783	4,384	316,650	13.84	13.44, 14.26
65	56,391	4,242	234,638	18.08	17.54, 18.63
70	38,353	4,025	159,352	25.26	24.49, 26.05
75	26,369	3,844	109,084	35.24	34.14, 36.37
80	20,245	3,518	75,438	46.63	45.12, 48.20
85	12,717	2,396	39,287	60.99	58.59, 63.48
**Validation dataset**					
**Landmark age**	**No. of persons in risk set**	**No. of events in risk set**	**Total PY**	**Incident rate per 1000 PY**	**95% CI**
40	40,366	432	165,142	2.62	2.38, 2.87
45	46,211	922	190,799	4.83	4.53, 5.15
50	45,535	1,292	184,269	7.01	6.64, 7.40
55	38,424	1,618	158,816	10.19	9.70, 10.70
60	33,827	1,963	146,458	13.40	12.82, 14.01
65	25,867	1,931	105,730	18.26	17.47, 19.10
70	17,770	1,882	72,754	25.87	24.73, 27.06
75	12,024	1,742	49,123	35.46	33.84, 37.17
80	9,150	1,562	33,894	46.09	43.86, 48.43
85	5,886	1,104	18,045	61.18	57.68, 64.90

Abbreviations: CI, confidence interval; PY, person-years

Web Table 7. Incidence rates and 95% confidence intervals of 10-year cardiovascular disease by landmark age in women in the derivation and validation dataset, Clinical Practice Research Datalink, Hospital Episode Statistics, and the Office for National Statistics, England, United Kingdom, 2004-2017 (in 5-year increments in landmark age for presentation)

Landmark age	No. of persons in risk set	No. of events in risk set	Total PY	Incident rate per 1000 PY	95% CI
**Derivation dataset**					
40	122,275	729	548,481	1.33	1.24, 1.43
45	128,314	1,230	564,880	2.18	2.06, 2.30
50	121,647	1,747	521,539	3.35	3.20, 3.51
55	102,911	2,160	447,279	4.83	4.63, 5.04
60	91,363	2,797	413,475	6.76	6.52, 7.02
65	74,393	3,142	322,513	9.74	9.41, 10.09
70	54,439	3,641	233,435	15.60	15.10, 16.11
75	40,284	4,084	174,299	23.43	22.72, 24.16
80	32,387	4,449	128,502	34.62	33.62, 35.65
85	24,220	3,939	81,855	48.12	46.64, 49.65
**Validation dataset**					
**Landmark age**	**No. of persons in risk set**	**No. of events in risk set**	**Total PY**	**Incident rate per 1000 PY**	**95% CI**
40	56,384	334	248,447	1.34	1.21, 1.50
45	58,382	581	253,417	2.29	2.11, 2.49
50	55,689	787	236,830	3.32	3.10, 3.56
55	47,630	886	206,764	4.29	4.01, 4.58
60	43,177	1,267	193,600	6.54	6.19, 6.91
65	34,491	1,435	146,263	9.81	9.32, 10.33
70	24,913	1,640	105,579	15.53	14.80, 16.30
75	18,664	1,954	80,846	24.17	23.12, 25.27
80	15,245	2,120	60,262	35.18	33.71, 36.71
85	11,561	1,900	38,254	49.67	47.48, 51.95

Abbreviations: CI, confidence interval; PY, person-years

Web Table 8. Hazard ratios[a] and 95% confidence intervals of risk predictors for cardiovascular disease by landmark age in men in the derivation dataset, Clinical Practice Research Datalink, Hospital Episode Statistics, and the Office for National Statistics, England, United Kingdom, 2004-2017 (in 5-year increments in landmark age for presentation)

Landmark age	Model ignoring statin initiation[b]											
	SBP	95% CI	BP Rx	95% CI	Total cholesterol	95% CI	HDL	95% CI	Current/Ever smoker	95% CI	Diabetes	95% CI
40	1.41	1.21, 1.64	1.76	1.49, 2.08	1.55	1.35, 1.78	0.58	0.49, 0.69	3.38	2.55, 4.48	2.68	1.99, 3.61
45	1.45	1.31, 1.61	1.92	1.72, 2.14	1.43	1.29, 1.59	0.65	0.58, 0.72	3.68	3.03, 4.47	1.49	1.14, 1.93
50	1.33	1.23, 1.43	1.70	1.56, 1.85	1.43	1.32, 1.55	0.68	0.63, 0.74	3.57	3.08, 4.12	1.67	1.37, 2.03
55	1.27	1.19, 1.36	1.52	1.42, 1.64	1.33	1.24, 1.43	0.75	0.71, 0.81	2.51	2.22, 2.83	1.54	1.30, 1.82
60	1.21	1.15, 1.29	1.43	1.34, 1.52	1.30	1.22, 1.39	0.77	0.73, 0.82	2.19	1.97, 2.43	1.50	1.30, 1.74
65	1.18	1.11, 1.25	1.44	1.36, 1.53	1.24	1.16, 1.33	0.84	0.80, 0.89	1.82	1.63, 2.02	1.46	1.27, 1.68
70	1.10	1.04, 1.17	1.42	1.33, 1.51	1.20	1.12, 1.29	0.90	0.85, 0.95	1.64	1.46, 1.84	1.35	1.18, 1.54
75	1.06	1.00, 1.13	1.33	1.24, 1.41	1.10	1.03, 1.19	0.91	0.86, 0.96	1.40	1.23, 1.59	1.32	1.16, 1.49
80	1.04	0.97, 1.11	1.44	1.34, 1.54	1.03	0.96, 1.11	0.94	0.89, 1.00	1.48	1.29, 1.71	1.37	1.21, 1.55
85	1.03	0.95, 1.11	1.35	1.23, 1.48	0.97	0.88, 1.06	0.98	0.91, 1.05	1.11	0.92, 1.36	1.33	1.15, 1.55
Landmark age	Model accounting for statin initiation[c]											
	SBP		BP Rx	95% CI	Total cholesterol	95% CI	HDL	95% CI	Current/Ever smoker	95% CI	Diabetes	95% CI
40	1.41	1.22, 1.64	1.78	1.51, 2.11	1.59	1.39, 1.82	0.58	0.49, 0.68	3.38	2.55, 4.49	2.94	2.18, 3.96
45	1.46	1.32, 1.62	1.95	1.75, 2.18	1.48	1.33, 1.64	0.64	0.57, 0.71	3.72	3.06, 4.51	1.63	1.25, 2.12
50	1.34	1.24, 1.44	1.74	1.60, 1.89	1.48	1.37, 1.61	0.67	0.62, 0.73	3.62	3.13, 4.19	1.83	1.50, 2.22
55	1.28	1.20, 1.37	1.56	1.45, 1.68	1.39	1.29, 1.49	0.74	0.70, 0.79	2.55	2.26, 2.88	1.67	1.42, 1.98
60	1.23	1.16, 1.30	1.47	1.38, 1.56	1.36	1.27, 1.45	0.76	0.72, 0.80	2.22	2.00, 2.46	1.61	1.39, 1.86
65	1.19	1.12, 1.26	1.48	1.39, 1.58	1.31	1.22, 1.40	0.83	0.79, 0.87	1.84	1.65, 2.05	1.56	1.36, 1.79
70	1.11	1.04, 1.18	1.45	1.36, 1.55	1.26	1.17, 1.36	0.89	0.84, 0.94	1.65	1.47, 1.86	1.43	1.25, 1.63
75	1.07	1.00, 1.14	1.34	1.26, 1.43	1.14	1.06, 1.23	0.90	0.85, 0.95	1.40	1.24, 1.60	1.40	1.24, 1.59
80	1.04	0.97, 1.11	1.45	1.35, 1.55	1.05	0.98, 1.14	0.94	0.89, 1.00	1.49	1.30, 1.71	1.43	1.26, 1.62
85	1.03	0.95, 1.11	1.36	1.24, 1.48	0.98	0.89, 1.07	0.97	0.91, 1.04	1.12	0.92, 1.36	1.36	1.17, 1.58

Abbreviations: BP Rx, blood pressure lowering medication use; CI, confidence interval; HDL, high-density lipoprotein; SBP, systolic blood pressure

[a] Hazard ratios are given per standard deviation increase for systolic blood pressure, total cholesterol, and HDL choleseterol.

[b] Model ignoring statin initiation: Ignoring statin treatment drop-in effect on CVD risk prediction

[c] Model accounting for statin initiation: Accounting for statin treatment drop-in effect on CVD risk prediction

Web Table 9. Hazard ratios[a] and 95% confidence interval of risk predictors for cardiovascular disease by landmark age in women in the derivation dataset, Clinical Practice Research Datalink, Hospital Episode Statistics, and the Office for National Statistics, England, United Kingdom, 2004-2017 (in 5-year increments in landmark age for presentation)

Landmark age	Model ignoring statin initiation[b]											
	SBP	95% CI	BP Rx	95% CI	Total cholesterol	95% CI	HDL	95% CI	Current/Ever smoker	95% CI	Diabetes	95% CI
40	1.56	1.32, 1.85	2.01	1.69, 2.38	1.47	1.19, 1.82	0.61	0.49, 0.77	2.61	1.85, 3.66	2.30	1.59, 3.33
45	1.40	1.24, 1.57	2.00	1.77, 2.26	1.55	1.36, 1.78	0.73	0.64, 0.84	2.85	2.22, 3.65	2.38	1.78, 3.19
50	1.34	1.22, 1.47	1.95	1.76, 2.15	1.31	1.17, 1.46	0.77	0.69, 0.85	2.95	2.41, 3.60	2.30	1.80, 2.96
55	1.22	1.12, 1.33	1.67	1.53, 1.82	1.21	1.10, 1.34	0.82	0.75, 0.90	3.26	2.75, 3.87	2.06	1.62, 2.62
60	1.25	1.16, 1.35	1.59	1.48, 1.72	1.11	1.01, 1.20	0.90	0.84, 0.97	2.46	2.12, 2.85	1.96	1.59, 2.41
65	1.19	1.10, 1.29	1.56	1.45, 1.67	1.14	1.05, 1.23	0.83	0.78, 0.89	2.10	1.82, 2.41	1.76	1.45, 2.14
70	1.14	1.06, 1.22	1.45	1.36, 1.55	1.02	0.94, 1.09	0.88	0.83, 0.94	1.97	1.72, 2.26	1.60	1.35, 1.88
75	1.09	1.01, 1.16	1.53	1.43, 1.63	1.16	1.09, 1.24	0.80	0.76, 0.85	1.59	1.38, 1.84	1.49	1.28, 1.73
80	1.12	1.05, 1.20	1.46	1.37, 1.57	1.05	0.98, 1.12	0.87	0.83, 0.92	1.67	1.45, 1.92	1.41	1.23, 1.62
85	1.03	0.96, 1.10	1.56	1.44, 1.69	0.97	0.91, 1.04	0.89	0.83, 0.95	1.44	1.21, 1.70	1.29	1.12, 1.50

Landmark age	Model accounting for statin initiation[c]											
	SBP		BP Rx	95% CI	Total cholesterol	95% CI	HDL	95% CI	Current/Ever smoker	95% CI	Diabetes	95% CI
40	1.56	1.32, 1.85	2.02	1.70, 2.39	1.51	1.22, 1.87	0.61	0.48, 0.76	2.60	1.85, 3.66	2.54	1.76, 3.67
45	1.40	1.24, 1.57	2.02	1.78, 2.28	1.60	1.39, 1.83	0.72	0.63, 0.83	2.85	2.22, 3.66	2.62	1.96, 3.51
50	1.34	1.22, 1.48	1.97	1.78, 2.17	1.35	1.21, 1.52	0.76	0.68, 0.84	2.96	2.42, 3.62	2.56	2.00, 3.29
55	1.23	1.13, 1.34	1.69	1.55, 1.84	1.26	1.14, 1.39	0.81	0.74, 0.88	3.29	2.77, 3.90	2.28	1.79, 2.90
60	1.26	1.17, 1.36	1.62	1.51, 1.75	1.15	1.06, 1.25	0.89	0.83, 0.95	2.50	2.16, 2.89	2.16	1.76, 2.67
65	1.20	1.11, 1.29	1.59	1.48, 1.71	1.19	1.10, 1.29	0.82	0.77, 0.87	2.14	1.86, 2.46	1.91	1.57, 2.32
70	1.14	1.06, 1.23	1.48	1.38, 1.58	1.06	0.99, 1.14	0.87	0.82, 0.92	1.99	1.73, 2.28	1.73	1.47, 2.04
75	1.09	1.02, 1.17	1.55	1.46, 1.66	1.20	1.12, 1.29	0.80	0.75, 0.84	1.60	1.39, 1.85	1.59	1.36, 1.84
80	1.13	1.06, 1.20	1.47	1.38, 1.58	1.07	1.00, 1.14	0.87	0.82, 0.92	1.68	1.46, 1.93	1.50	1.31, 1.72
85	1.03	0.96, 1.10	1.57	1.45, 1.70	0.99	0.92, 1.06	0.89	0.83, 0.94	1.45	1.22, 1.71	1.34	1.15, 1.55

Abbreviations: BP Rx, blood pressure lowering medication use; CI, confidence interval; HDL, high-density lipoprotein; SBP, systolic blood pressure

[a] Hazard ratios are given per standard deviation increase for systolic blood pressure, total cholesterol, and HDL choleseterol.

[b] Model ignoring statin initiation: Ignoring statin treatment drop-in effect on CVD risk prediction

[c] Model accounting for statin initiation: Accounting for statin treatment drop-in effect on CVD risk prediction

Web Table 10. Sex-specific means and 95% confidence intervals of the standard 10-year cardiovascular risk predictions versus the statin-naïve 10-year cardiovascular risk predictions by landmark age in the validation dataset, Clinical Practice Research Datalink, Hospital Episode Statistics, and the Office for National Statistics, England, United Kingdom, 2004-2017 (in 5-year increments in landmark age for presentation)

Landmark age	Men, mean of 10-year predicted risk (%)				Women, mean of 10-year predicted risk (%)			
	standard predicted risk	95% CI	statin-naïve predicted risk	95% CI	standard predicted risk	95% CI	statin-naïve predicted risk	95% CI
40	3.23	3.21, 3.25	3.30	3.28, 3.32	1.45	1.44, 1.46	1.47	1.46, 1.48
45	4.88	4.85, 4.90	5.01	4.98, 5.04	2.42	2.40, 2.43	2.47	2.45, 2.48
50	7.69	7.65, 7.72	7.97	7.93, 8.01	3.55	3.53, 3.57	3.65	3.63, 3.67
55	10.46	10.42, 10.50	10.92	10.88, 10.96	5.07	5.05, 5.09	5.25	5.22, 5.28
60	13.32	13.28, 13.37	14.08	14.03, 14.13	6.90	6.88, 6.93	7.20	7.17, 7.23
65	17.33	17.27, 17.39	18.46	18.40, 18.53	9.91	9.87, 9.94	10.42	10.38, 10.46
70	23.33	23.26, 23.40	24.70	24.62, 24.78	15.42	15.36, 15.47	16.23	16.17, 16.29
75	30.98	30.89, 31.06	31.98	31.88, 32.07	21.92	21.84, 22.00	22.81	22.72, 22.90
80	39.29	39.15, 39.42	40.11	39.97, 40.25	30.44	30.34, 30.54	31.22	31.12, 31.32
85	47.33	47.18, 47.48	47.90	47.75, 48.05	40.15	40.03, 40.27	40.72	40.60, 40.85

Abbreviations: CI, confidence interval

Web Table 11. Sex-specific medians and interquartile ranges of the standard 10-year cardiovascular risk predictions versus the statin-naïve 10-year cardiovascular risk predictions by landmark age in the validation dataset, Clinical Practice Research Datalink, Hospital Episode Statistics, and the Office for National Statistics, England, United Kingdom, 2004-2017 (in 5-year increments in landmark age for presentation)

Landmark age	Men, median of 10-year predicted risk (%)				Women, median of 10-year predicted risk (%)			
	standard predicted risk	IQR	statin-naïve predicted risk	IQR	standard predicted risk	IQR	statin-naïve predicted risk	IQR
40	2.87	2.13, 3.83	2.91	2.15, 3.88	1.14	0.85, 1.67	1.15	0.85, 1.69
45	4.2	3.15, 5.96	4.28	3.19, 6.09	1.9	1.42, 2.82	1.92	1.42, 2.86
50	6.69	5.25, 9.57	6.87	5.35, 9.92	2.83	2.17, 4.24	2.88	2.19, 4.34
55	9.49	7.83, 12.54	9.83	8.03, 13.12	4.23	3.40, 6.34	4.34	3.45, 6.56
60	12.28	10.36, 15.77	12.9	10.75, 16.73	6.05	5.00, 8.40	6.27	5.13, 8.76
65	16.18	13.96, 20.02	17.16	14.65, 21.34	8.87	7.39, 11.89	9.27	7.64, 12.55
70	22.29	19.42, 26.56	23.56	20.34, 28.21	14.55	12.24, 17.96	15.25	12.73, 18.96
75	30.95	26.80, 34.07	31.71	27.49, 35.23	22.1	17.18, 25.40	22.9	17.72, 26.54
80	41.01	32.54, 43.10	41.79	33.11, 44.03	31.34	24.62, 33.81	32.08	25.16, 34.70
85	49.39	40.46, 50.33	50.03	40.84, 50.93	42.08	35.11, 43.12	42.74	35.61, 43.81

Abbreviations: IQR, interquartile range

Web Table 12. Number needed to screen (NNS) and number needed to treat (NNT) per event prevented estimated using standard 10-year cardiovascular risk predictions versus statin-naïve 10-year cardiovascular risk predictions by landmark age in the validation dataset, Clinical Practice Research Datalink, Hospital Episode Statistics, and the Office for National Statistics, England, United Kingdom, 2004-2017 (in 5-year increments in landmark age for presentation)

Landmark age	Men					Women				
	Number of people in the risk set at 10-year	NNS		NNT		Number of people in the risk set at 10-year	NNS		NNT	
		use standard risk predictions	use statin-naïve risk predictions	use standard risk predictions	use statin-naïve risk predictions		use standard risk predictions	use statin-naïve risk predictions	use standard risk predictions	use statin-naïve risk predictions
40	2,414	604	483	7	7	4,105	2,053	2,053	7	9
45	3,082	92	83	7	7	4,044	462	462	6	7
50	3,116	26	23	7	7	3,918	266	227	7	7
55	3,244	13	12	7	7	3,595	112	102	7	7
60	3,465	8	8	7	7	3,767	54	46	7	8
65	2,797	6	6	6	6	2,924	13	13	7	7
70	2,514	5	5	5	5	2,754	7	7	7	7
75	2,198	5	5	5	5	2,821	6	6	6	6
80	1,762	5	5	5	5	2,580	5	5	5	5
85	1,154	4	4	4	4	2,021	4	4	4	4

Web Table 13. Overall Brier score[a,b] and C-index[a] in the validation subset of individuals who remained statin-naïve during follow-up, Clinical Practice Research Datalink, Hospital Episode Statistics, and the Office for National Statistics, England, United Kingdom, 2004-2017

	Brier Score	95% CI	C-index	95% CI
**Men**				
Model ignoring statin initiation[c]	0.3511	0.3485, 0.3537	0.7588	0.7567, 0.7695
Model accounting for statin initiation[d]	0.3448	0.3422, 0.3493	0.7591	0.7570, 0.7612
Difference	-0.0063	-0.0064, -0.0062	0.0003	0.0002, 0.0004
**Women**				
Model ignoring statin initiation	0.2603	0.2583, 0.2624	0.8032	0.8012, 0.8053
Model accounting for statin initiation	0.2572	0.2551, 0.2592	0.8033	0.8013, 0.8054
Difference	-0.0032	-0.0033, -0.0031	0.0001	0.0001, 0.0001

[a] Overall Brier score and C-index were calculated by "stacking" the data at each landmark age into a single dataset (the stacked dataset).

[b] Brier scores were calculated only using information from individuals with at least 10 years follow-up or had an event within 10 years from each landmark age.

[c] Model ignoring statin initiation: Ignoring statin treatment drop-in effect on CVD risk prediction

[d] Model accounting for statin initiation: Accounting for statin treatment drop-in effect on CVD risk prediction

Web Table 14. Ten-year cardiovascular disease risk classification comparing statin-naïve CVD risk predictions versus standard CVD risk predictions for men in the validation subset of individuals who remained statin-naïve during follow-up, Clinical Practice Research Datalink, Hospital Episode Statistics, and the Office for National Statistics, England, United Kingdom, 2004-2017 (in landmark ages at 40, 50, 60, 70 for presentation[a])

**Landmark age 40**			
	**Statin-naïve 10-year CVD risk predictions**		
**Standard 10-year CVD risk predictions**	<10%	≥10%	Total
Events within 10 years[b]			
<10%	349	3	352
≥10%	0	10	10
Subtotal	359	13	362
Events free at 10-year[b]			
<10%	1,821	1	1,822
≥10%	0	8	8
Subtotal	1,821	9	1,830
	Categorical NRI[c]	95% CI	
event[b]	0.0083	-0.0011, 0.0177	
non-event[b]	-0.0005	-0.0016, 0.0005	
overall	0.0077	-0.0017, 0.0172	
	IDI	95% CI	
event[b]	0.0011	0.0009, 0.0013	
non-event[b]	-0.0005	-0.0006, -0.0005	
overall	0.0005	0.0003, 0.0008	
	Continuous NRI[d]	95% CI	
event[e]	0.8724	0.8161, 0.9288	
non-event[e]	-0.8627	-0.8685, -0.8569	
overall	0.0097	-0.0482, 0.0676	

**Landmark age 50**			
	**Statin-naïve 10-year CVD risk predictions**		
**Standard 10-year CVD risk predictions**	<10%	≥10%	Total
Events within 10 years			
<10%	617	45	662
≥10%	0	345	345
Subtotal	617	390	1,007
Events free at 10-year			
<10%	1,195	52	1,247
≥10%	0	278	278
Subtotal	1,195	330	1,525
	Categorical NRI	95% CI	
event	0.0447	0.0316, 0.0577	
non-event	-0.0341	-0.0434, -0.0248	
overall	0.0106	-0.0054, 0.0266	
	IDI	95% CI	
event	0.0038	0.0036, 0.0041	
non-event	-0.0024	-0.0025, -0.0023	
overall	0.0014	0.0011, 0.0017	
	Continuous NRI	95% CI	
event	0.9012	0.8577, 0.9446	
non-event	-0.8644	-0.8698, -0.8590	
overall	0.0368	-0.0096, 0.0831	

**Landmark age 60**			
	**Statin-naïve 10-year CVD risk predictions**		
**Standard 10-year CVD risk predictions**	<10%	≥10%	Total
Events within 10 years[b]			
<10%	154	41	195
≥10%	0	1,205	1,205
Subtotal	154	1,246	1,400
Events free at 10-year[b]			
<10%	126	46	172
≥10%	0	930	930
Subtotal	126	976	1,102
	Categorical NRI	95% CI	
event	0.0293	0.0203, 0.0382	
non-event	-0.0417	-0.0538, -0.0297	
overall	-0.0125	-0.0275 ,0.0026	
	IDI	95% CI	
event	0.0089	0.0085, 0.0093	
non-event[b]	-0.0070	-0.0073, -0.0067	
overall	0.0019	0.0014, 0.0024	
	Continuous NRI	95% CI	
event	0.9657	0.9486, 0.9827	
non-event	-0.9569	-0.9610, -0.9528	
overall	0.0088	-0.0105, 0.0281	

**Landmark age 70**			
	**Statin-naïve 10-year CVD risk predictions**		
**Standard 10-year CVD risk predictions**	<10%	≥10%	Total
Events within 10 years			
<10%	0	0	0
≥10%	0	1,370	1,370
Subtotal	0	1,370	1,370
Events free at 10-year			
<10%	0	0	0
≥10%	0	503	503
Subtotal	0	503	503
	Categorical NRI	95% CI	
event	0.0000	0.0000, 0.0000	
non-event	0.0000	0.0000, 0.0000	
overall	0.0000	0.0000, 0.0000	
	IDI	95% CI	
event	0.0150	0.0146, 0.0155	
non-event	-0.0135	-0.0140, -0.0130	
overall	0.0015	0.0001, 0.0002	
	Continuous NRI	95% CI	
event	0.9755	0.9566, 0.9945	
non-event	-0.9830	-0.9894, -0.9765	
overall	-0.0075	-0.0317, 0.0168	

Abbreviations: CVD, cardiovascular disease; IDI, integrated discrimination improvement; NRI, net reclassification improvement

[a] The results are presented in 10-year increments in landmark age at 40, 50, 60, 70. Above landmark age 69 for men in the subset of individuals with no future statin initiation after the landmark age, the predicted 10-year CVD risk for all individuals in the risk set were greater than 10% for both standard risk predictions and statin-naïve risk predictions, therefore there was no movement between the 2 categories and the categorical NRIs were 0 for those older landmark age groups.

[b] Categorical NRI and IDI were calculated using information from individuals who were not censored at 10 years (either with CVD events within 10 years or events free at 10-year)

[c] Categorical NRI was calculated based on the four categories of predicted risk of <10% and ≥10%.

[d] Continuous NRI (the prospective form NRI) was calculated based on continuous predicted risk and used information from all individuals, including the censored ones.

[e] Events and non-events for continuous NRI (the prospective form of NRI) were the expected results estimated using the Kaplan-Meier approach, so such prospective form of NRI uses the whole sample and does not require the restriction to the non-censored ones.

Web Table 15. Ten-year cardiovascular disease risk classification comparing statin-naïve CVD risk predictions versus standard CVD risk predictions for women in the validation subset of individuals who remained statin-naïve during follow-up, Clinical Practice Research Datalink, Hospital Episode Statistics, and the Office for National Statistics, England, United Kingdom, 2004-2017 (in landmark ages at 40, 50, 60, 70 for presentation[a])

Landmark age 40

	**Statin-naïve 10-year CVD risk predictions**		
**Standard 10-year CVD risk predictions**	<10%	≥10%	Total
Events within 10 years[b]			
<10%	283	0	283
≥10%	0	5	5
Subtotal	283	5	288
Events free at 10-year[b]			
<10%	3,611	2	3,613
≥10%	0	3	3
Subtotal	3,611	5	3,616
	Categorical NRI[c]	95% CI	
event[b]	0.0000	0.0000, 0.0000	
non-event[b]	-0.0006	-0.0013, 0.0002	
overall	-0.0006	-0.0013, 0.0002	
	IDI	95% CI	
event[b]	0.0006	0.0003, 0.0008	
non-event[b]	-0.0001(-0.0002,	-0.0002, -0.0001	
overall	0.0004	0.0002, 0.0007	
	Continuous NRI[d]	95% CI	
event[e]	0.8415	0.7842 0.8988	
non-event[e]	-0.7492	-0.7549, -0.7434	
overall	0.0923	0.0338, 0.1508	

Landmark age 50

	**Statin-naïve 10-year CVD risk predictions**		
**Standard 10-year CVD risk predictions**	<10%	≥10%	Total
Events within 10 years			
<10%	596	8	604
≥10%	0	35	35
Subtotal	596	43	639
Events free at 10-year			
<10%	2,790	6	2,796
≥10%	0	31	31
Subtotal	2,790	37	2,827
	Categorical NRI	95% CI	
event	0.0125	0.0038, 0.0212	
non-event	-0.0021	-0.0038, -0.0004	
overall	0.0104	0.0016, 0.0192	
	IDI	95% CI	
event	0.0017	0.0015, 0.0020	
non-event	-0.0008	-0.0008, -0.0007	
overall	0.0010	0.0007, 0.0012	
	Continuous NRI	95% CI	
event	0.8152	0.7309, 0.8994	
non-event	-0.8004	-0.8058, -0.7950	
overall	0.0148	-0.0721, 0.1017	

Landmark age 60

	**Statin-naïve 10-year CVD risk predictions**		
**Standard 10-year CVD risk predictions**	<10%	≥10%	Total
Events within 10 years[b]			
<10%	732	34	766
≥10%	0	181	181
Subtotal	732	215	947
Events free at 10-year[b]			
<10%	1,813	56	1,869
≥10%	0	163	163
Subtotal	1,813	219	2,032
	Categorical NRI	95% CI	
event	0.0359	0.0238, 0.0480	
non-event	-0.0276	-0.0348, -0.0203	
overall	0.0083	-0.0057, 0.0024	
	IDI	95% CI	
event	0.0037	0.0035, 0.0040	
non-event[b]	-0.0027	-0.0028, -0.0026	
overall	0.0010	0.0008, 0.0013	
	Continuous NRI	95% CI	
event	0.9410	0.9230, 0.9589	
non-event	-0.8972	-0.9022, -0.8922	
overall	0.0438	0.0234, 0.0641	

Landmark age 70

	**Statin-naïve 10-year CVD risk predictions**		
**Standard 10-year CVD risk predictions**	<10%	≥10%	Total
Events within 10 years			
<10%	17	5	22
≥10%	0	1,204	1,204
Subtotal	17	1,209	1,226
Events free at 10-year			
<10%	6	2	8
≥10%	0	877	877
Subtotal	6	879	885
	Categorical NRI	95% CI	
event	0.0041	0.0005, 0.0077	
non-event	-0.0023	-0.0054, 0.0009	
overall	0.0018	-0.0029, 0.0066	
	IDI	95% CI	
event	0.0091	0.0088, 0.0095	
non-event	-0.0080	-0.0083, -0.0078	
overall	0.0011	0.0007, 0.0015	
	Continuous NRI	95% CI	
event	0.9734	0.9549, 0.9918	
non-event	-0.9621	-0.9674, -0.9569	
overall	0.0113	-0.0106, 0.0332	

Abbreviations: CVD, cardiovascular disease; IDI, integrated discrimination improvement; NRI, net reclassification improvement

[a] The results are presented in 10-year increments in landmark age at 40, 50, 60, 70. Above landmark age 76 for women in the subset of individuals with no future statin initiation after the landmark age, the predicted 10-year CVD risk for all individuals in the risk set were greater than 10% for both standard risk predictions and statin-naïve risk predictions, therefore there was no movement between the 2 categories and the categorical NRIs were 0 for those older landmark age groups.

[b] Categorical NRI and IDI were calculated using information from individuals who were not censored at 10 years (either with CVD events within 10 years or events free at 10-year)

[c] Categorical NRI was calculated based on the four categories of predicted risk of <10% and ≥10%.

[d] Continuous NRI (the prospective form NRI) was calculated based on continuous predicted risk and used information from all individuals, including the censored ones.

[e] Events and non-events for continuous NRI (the prospective form of NRI) were the expected results estimated using the Kaplan-Meier approach, so such prospective form of NRI uses the whole sample and does not require the restriction to the non-censored ones.

Web Table 16. Number needed to screen (NNS) and number needed to treat (NNT) per event prevented estimated using standard 10-year cardiovascular risk predictions versus statin-naïve 10-year cardiovascular risk predictions by landmark age in the validation subset of individuals who remained statin-naïve during follow-up, Clinical Practice Research Datalink, Hospital Episode Statistics, and the Office for National Statistics, England, United Kingdom, 2004-2017 (in 5-year increments in landmark age for presentation)

Landmark age	Men					Women				
	Number of people in the risk set at 10-year	NNS		NNT		Number of people in the risk set at 10-year	NNS		NNT	
		use standard risk predictions	use statin-naïve risk predictions	use standard risk predictions	use statin-naïve risk predictions		use standard risk predictions	use statin-naïve risk predictions	use standard risk predictions	use statin-naïve risk predictions
40	2,192	877	674	7	7	3,904	3,123	3,123	6	8
45	2,657	114	104	7	7	3,721	677	677	6	6
50	2,532	29	26	7	7	3,466	396	322	8	7
55	2,491	14	13	7	7	2,971	140	128	6	7
60	2,502	8	8	7	7	2,979	66	55	8	8
65	2,041	6	6	6	6	2,193	14	13	7	7
70	1,873	5	5	5	5	2,111	7	7	7	7
75	1,813	5	5	5	5	2,294	6	6	6	6
80	1,550	5	5	5	5	2,242	5	5	5	5
85	1,046	4	4	4	4	1,878	4	4	4	4

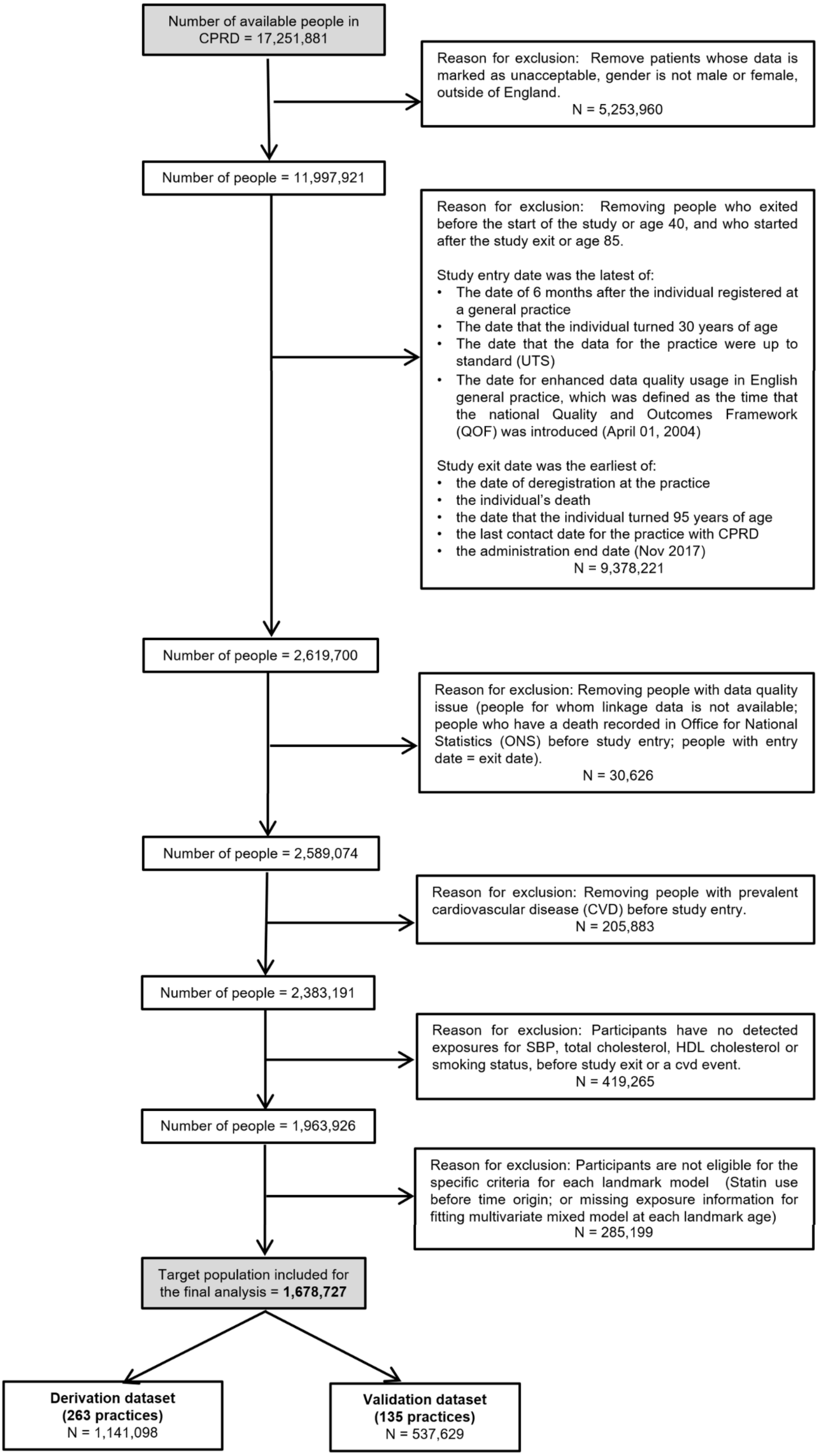

Web Figure 1. Flow chart of study population selection, Clinical Practice Research Datalink, Hospital Episode Statistics, and the Office for National Statistics, England, United Kingdom, 2004-2017

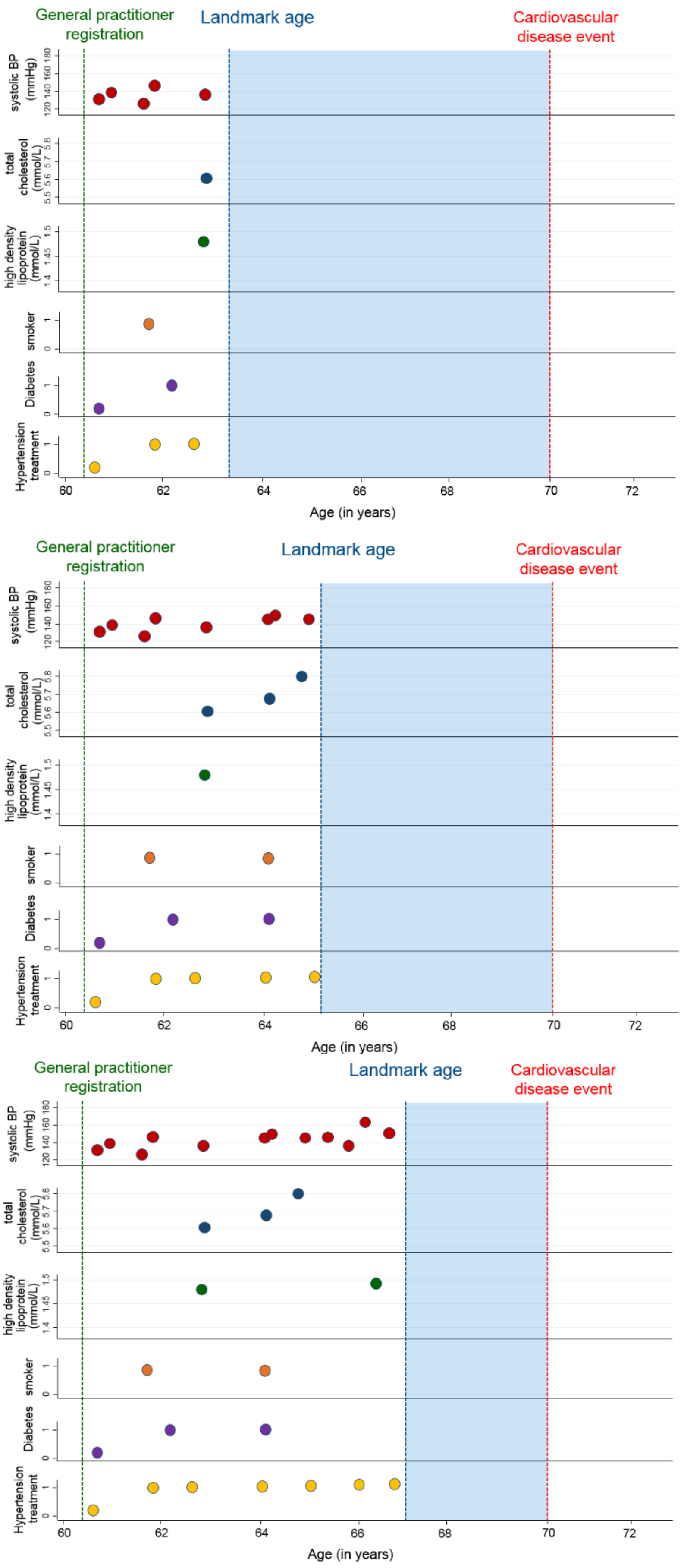

Web Figure 2: Schematic of landmark age approach

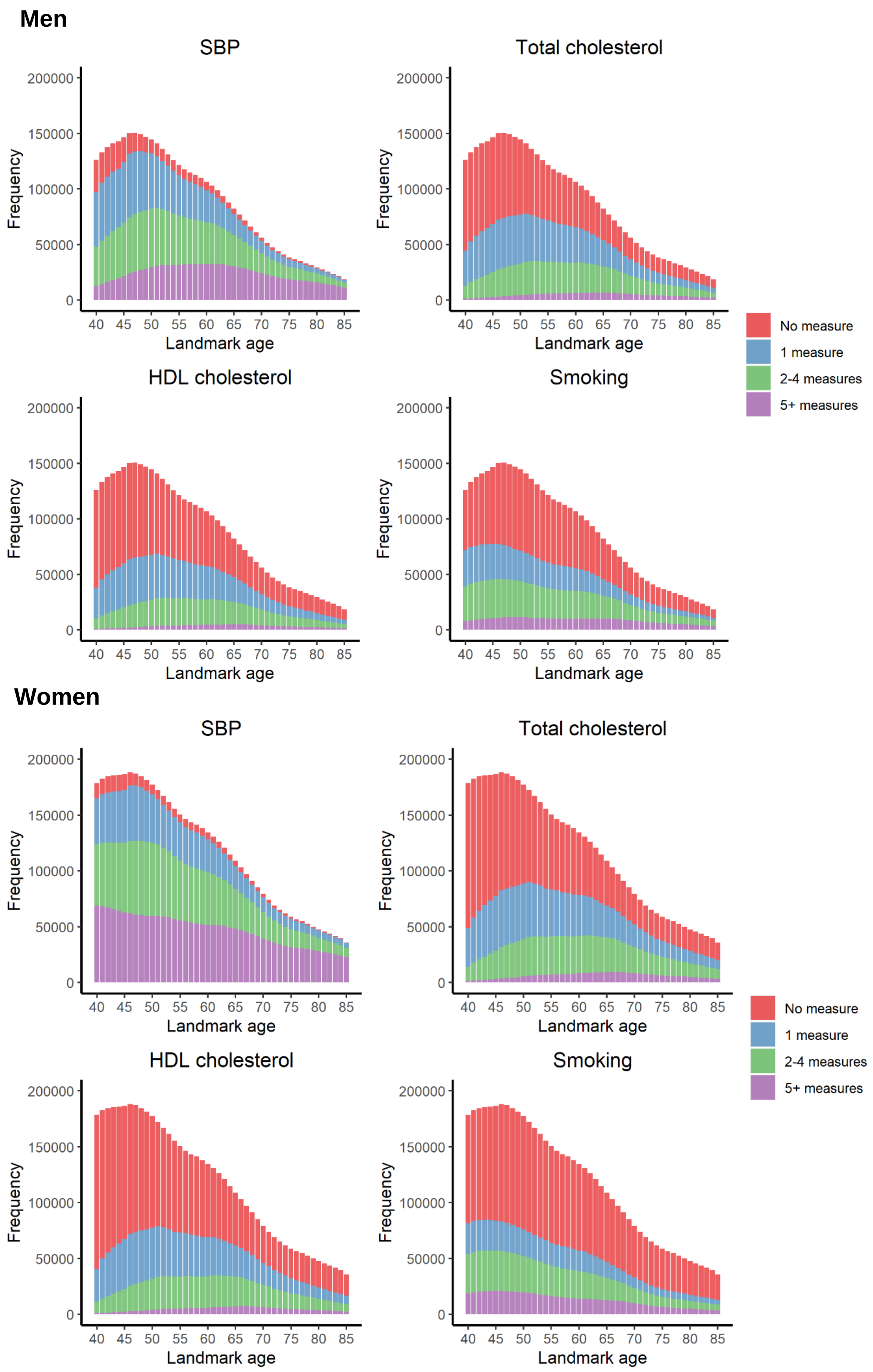

Web Figure 3: Sex-specific distribution of the number of measurements of systolic blood pressure, total cholesterol, HDL cholesterol, and smoking status in each landmark age model risk set, Clinical Practice Research Datalink, Hospital Episode Statistics, and the Office for National Statistics, England, United Kingdom, 2004-2017

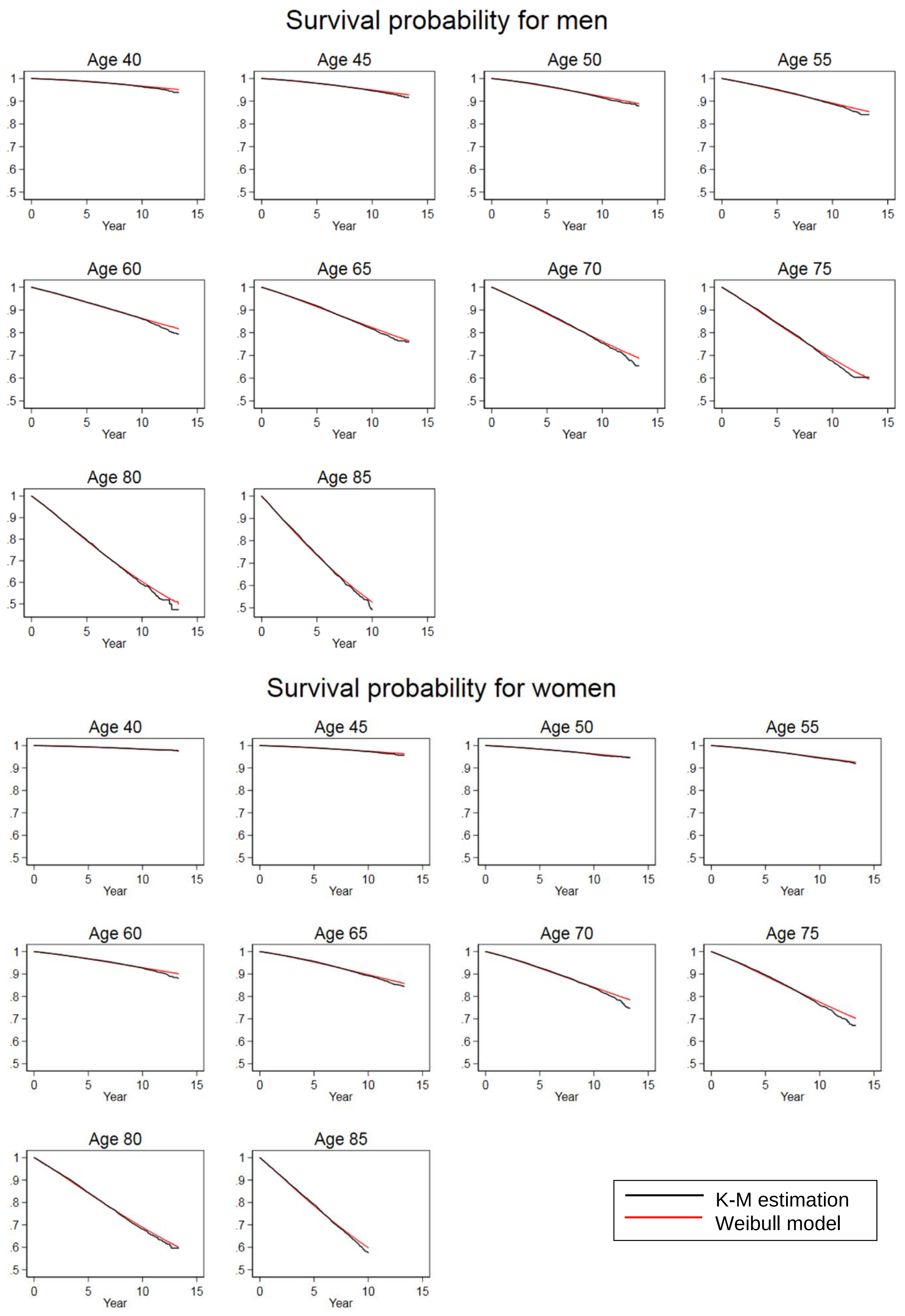

Web Figure 4. Sex-specific survival probablities estimated from Weibull model with no covariates versus Kaplan-Meier survival function by landmark age in the derivation dataset, Clinical Practice Research Datalink, Hospital Episode Statistics, and the Office for National Statistics, England, United Kingdom, 2004-2017

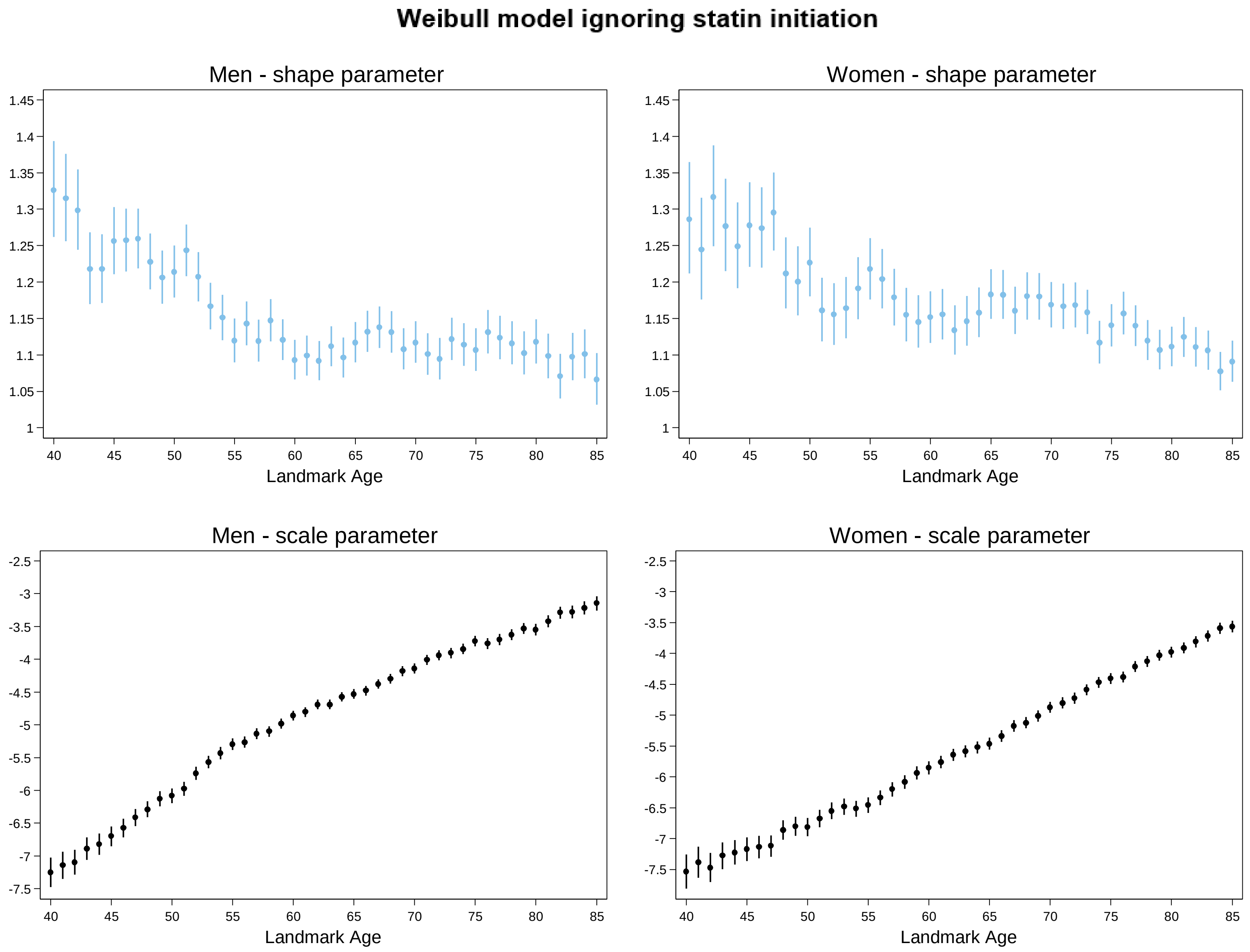

Web Figure 5. Sex-specific distributions of shape and scale parameters from parametric Weibull models ignoring statin initiation by landmark age in the derivation dataset, Clinical Practice Research Datalink, Hospital Episode Statistics, and the Office for National Statistics, England, United Kingdom, 2004-2017

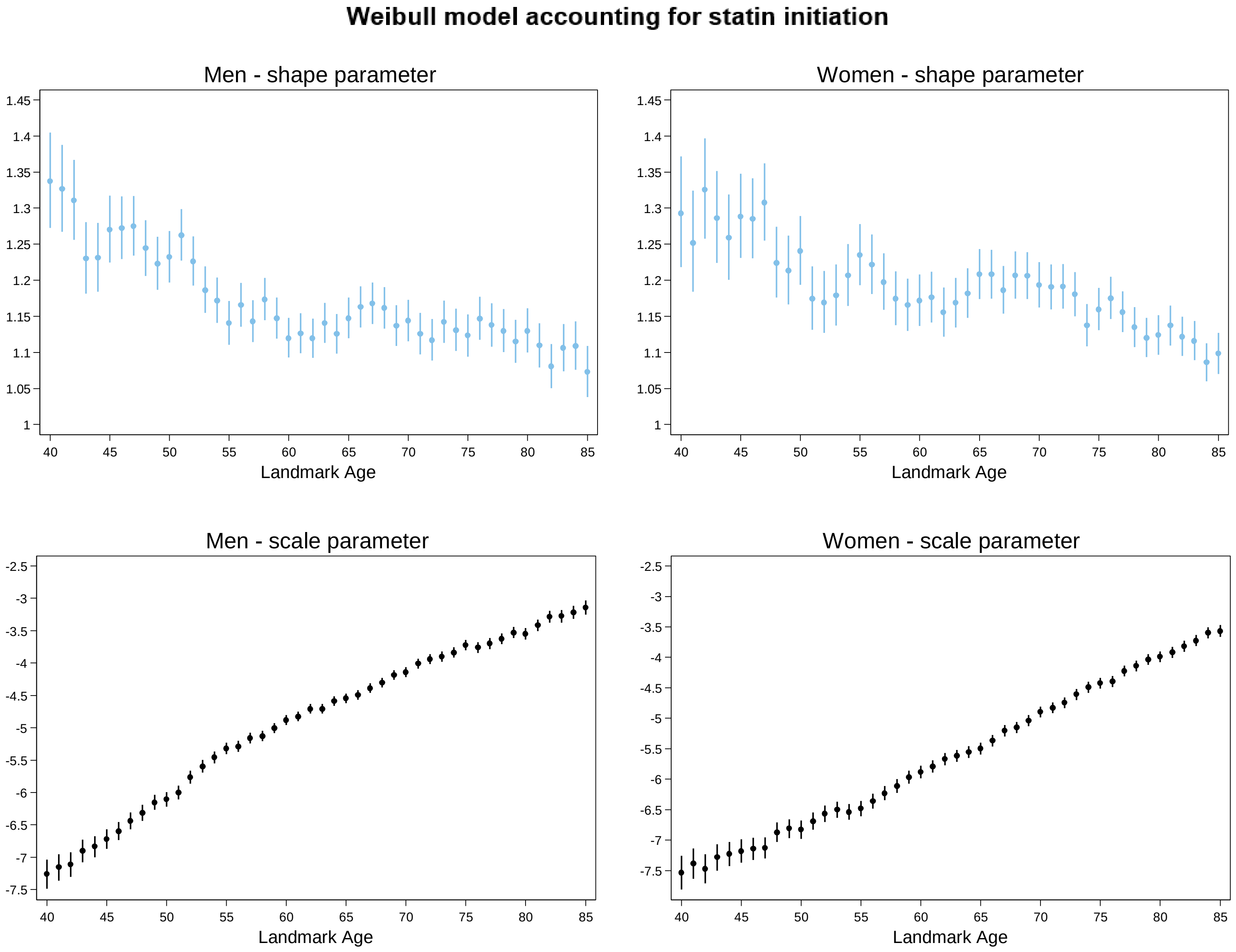

Web Figure 6. Sex-specific distributions of shape and scale parameters from parametric Weibull models accounting for statin initiation by landmark age in the derivation dataset, Clinical Practice Research Datalink, Hospital Episode Statistics, and the Office for National Statistics, England, United Kingdom, 2004-2017

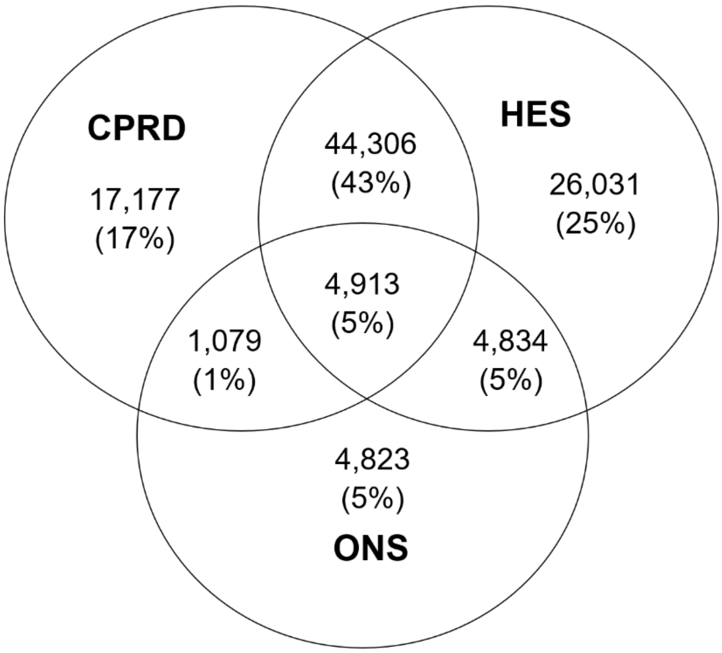

Web Figure 7. Venn diagram of the incident cardiovascular events during follow-up recorded from primary care data in Clinical Practice Research Datalink (CPRD) (n=67,475 first events identified), secondary care data in Hospital Episode Statistics (HES) (n=80,084 first events identified), and mortality records in Office for National Statistics (ONS) (n=15,649 first events identified), England, United Kingdom, 2004-2017

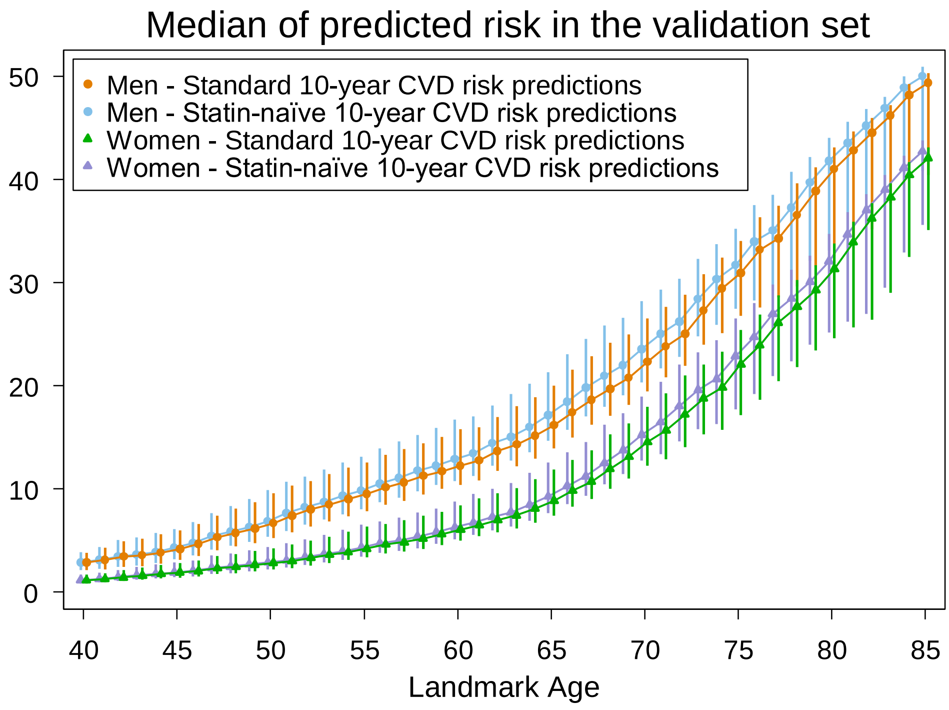

Web Figure 8. Sex-specific medians and interquartile ranges (IQRs) of the statin-naïve 10-year CVD risk predictions versus the standard 10-year CVD risk predictions by landmark age in the validation dataset, Clinical Practice Research Datalink, Hospital Episode Statistics, and the Office for National Statistics, England, United Kingdom, 2004-2017

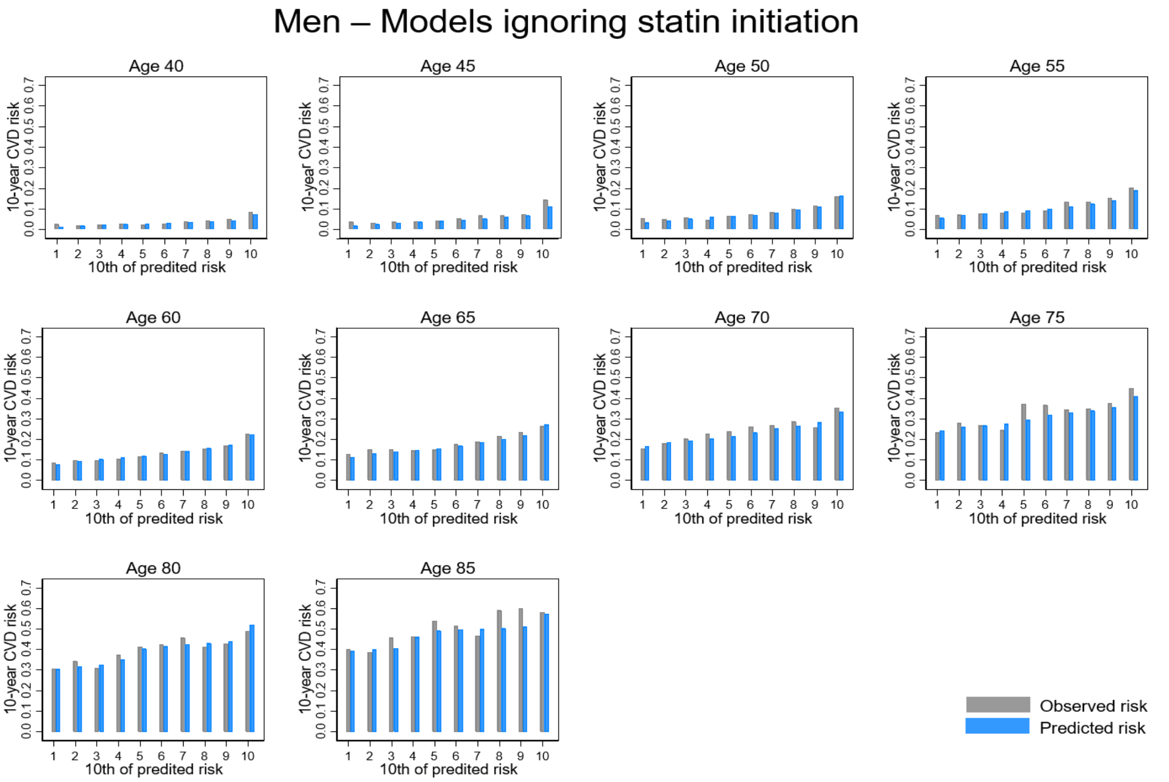

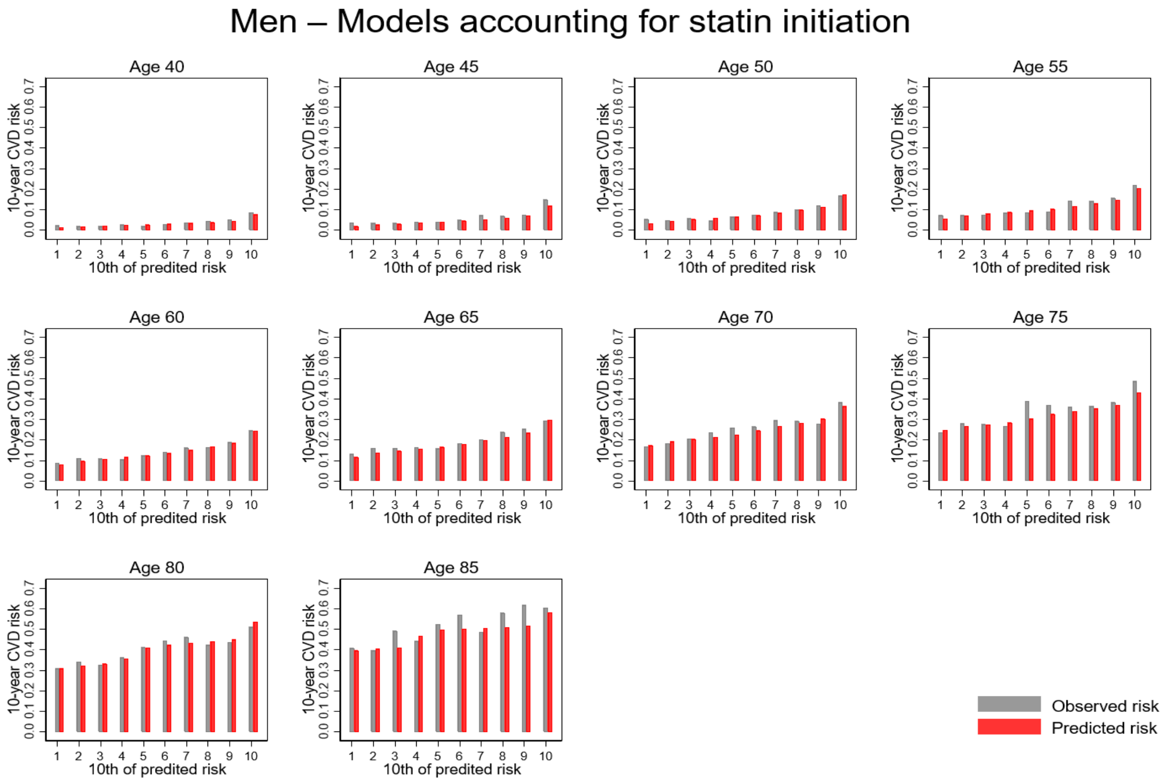

Web Figure 9. Calibration plots for men by landmark age in the validation set using models ignoring statin initiation and models accounting for statin initiation, Clinical Practice Research Datalink, Hospital Episode Statistics, and the Office for National Statistics, England, United Kingdom, 2004-2017. The plots show the mean predicted 10-year risk versus observed 10-year risk of cardiovascular disease, by deciles of predicted risk. Observed follow-up time was used to calculate the mean observed risks to assess calibration for the standard 10-year CVD risk predictions in models ignoring statin initiation; counterfactual follow-up time was used to calculate the mean observed risks to assess calibration for the statin-naïve 10-year CVD risk predictions in models accounting for statin initiation.

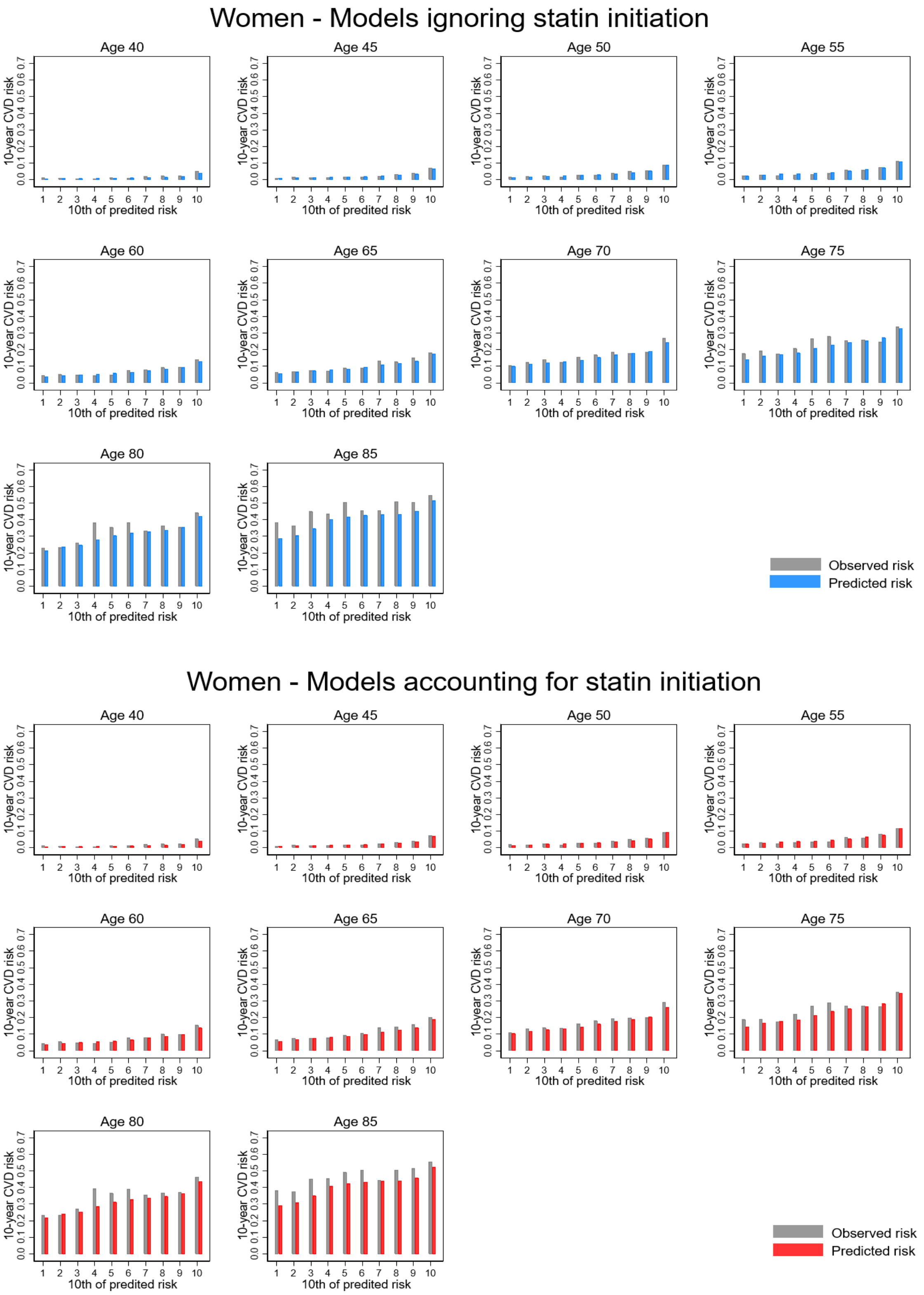

Web Figure 10. Calibration plots for women by landmark age in the validation set using models ignoring statin initiation and models accounting for statin initiation, Clinical Practice Research Datalink, Hospital Episode Statistics, and the Office for National Statistics, England, United Kingdom, 2004-2017. The plots show the mean predicted 10-year risk versus observed 10-year risk of cardiovascular disease, by deciles of predicted risk. Observed follow-up time was used to calculate the mean observed risks to assess calibration for the standard 10-year CVD risk predictions in models ignoring statin initiation; counterfactual follow-up time was used to calculate the mean observed risks to assess calibration for the statin-naïve 10-year CVD risk predictions in models accounting for statin initiation.

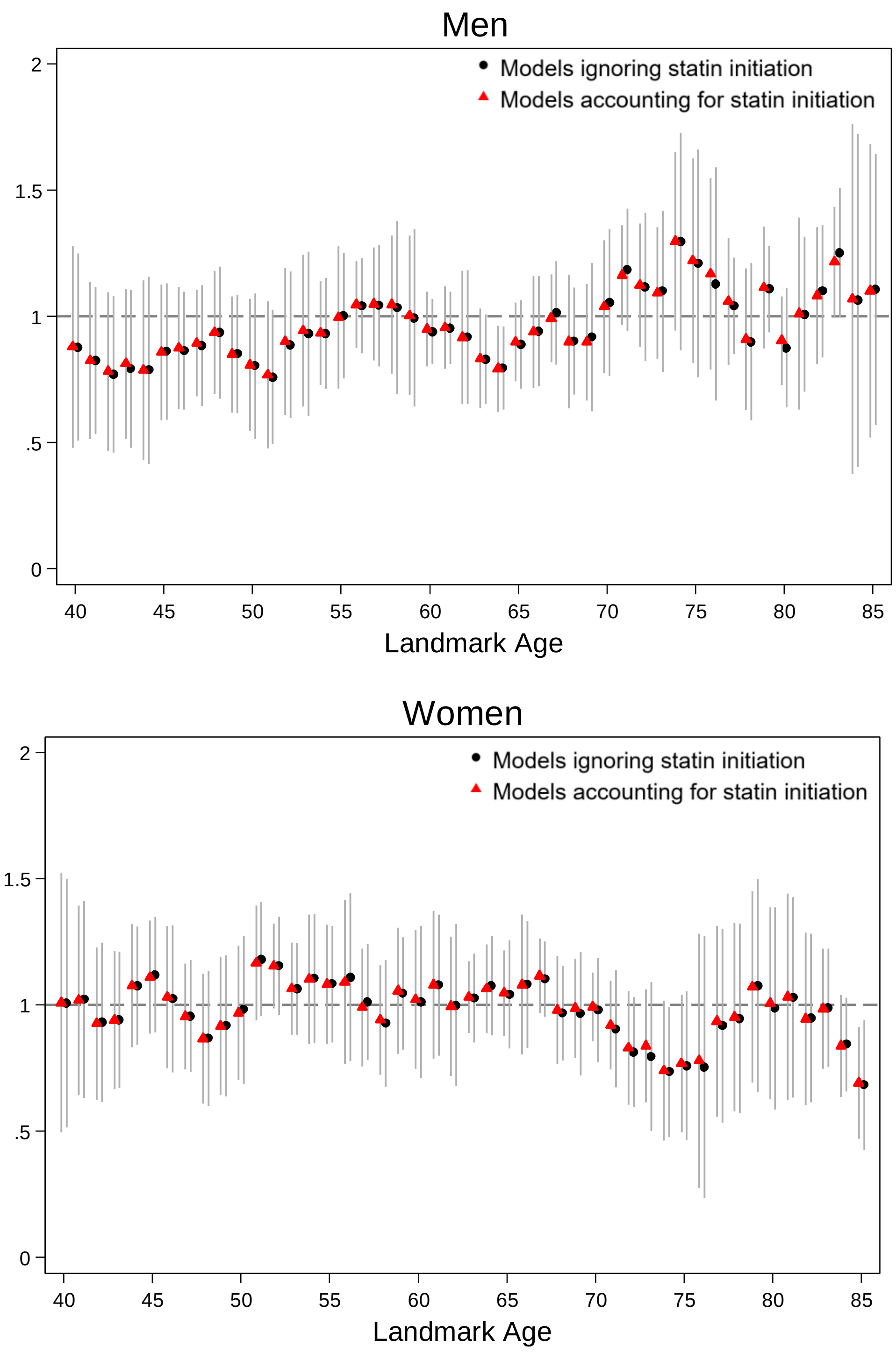

Web Figure 11. Comparison of calibration slopes for men and women by landmark age in the validation set using models ignoring statin initiation and models accounting for statin initiation, Clinical Practice Research Datalink, Hospital Episode Statistics, and the Office for National Statistics, England, United Kingdom, 2004-2017. The calibration slope is the regression coefficient from the linear model: $\ln(-\ln(1-Y_{obs})) = \beta 0 + \beta 1 * \ln(-\ln(1-Y_{pred}))$, where Y_{obs} and Y_{pred} are the mean observed and predicted risks within each decile of predicted risk. Observed follow-up time was used to calculate the mean observed risks to assess calibration for the standard 10-year CVD risk predictions in models ignoring statin initiation; counterfactual follow-up time was used to calculate the mean observed risks to assess calibration for the statin-naïve 10-year CVD risk predictions in models accounting for statin initiation.

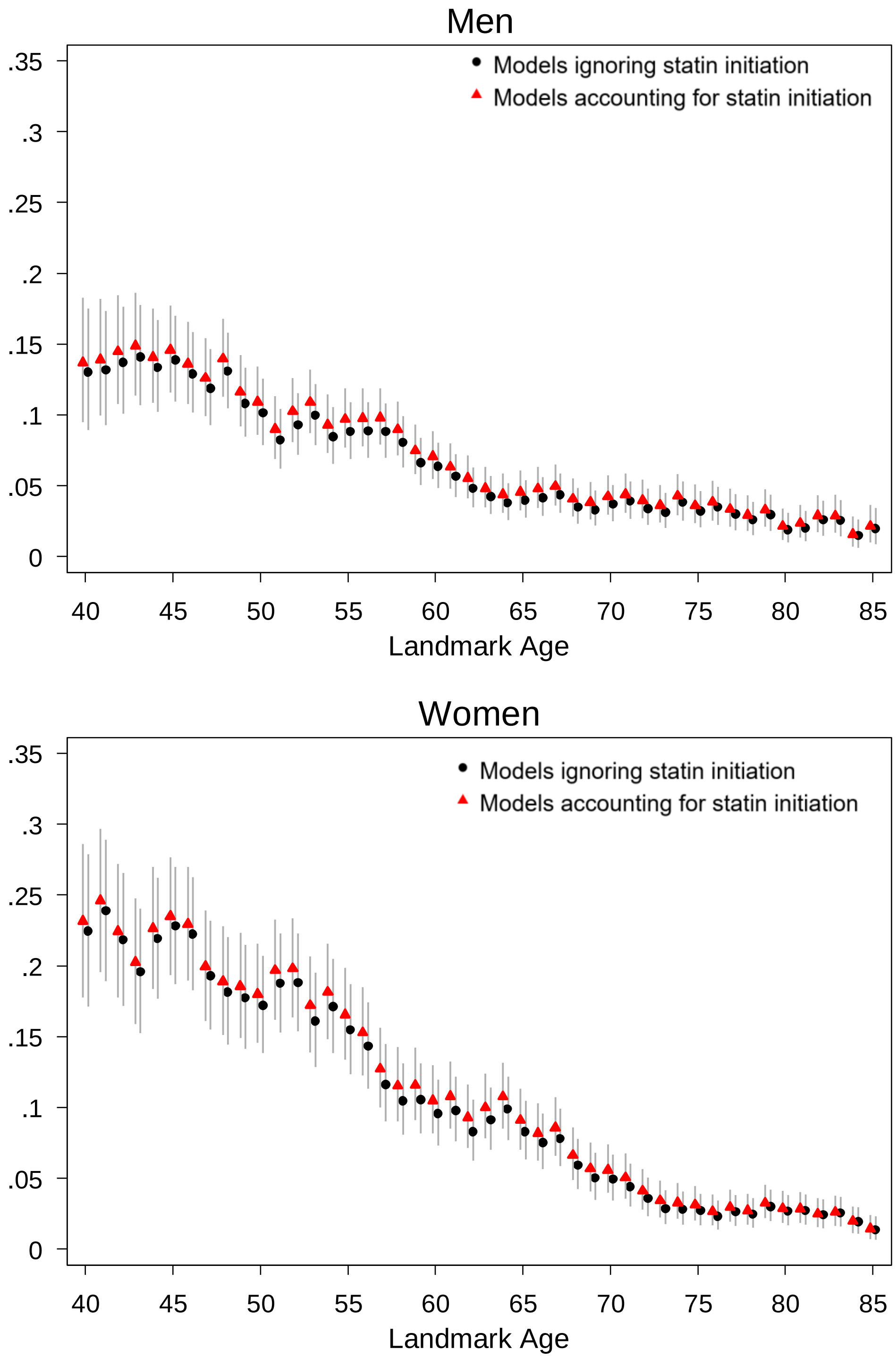

Web Figure 12. Comparison of R squared values for models ignoring statin initiation versus models accounting for statin initiation for the prediction of 10-year cardiovascular disease at each landmark age in the validation dataset, Clinical Practice Research Datalink, Hospital Episode Statistics, and the Office for National Statistics, England, United Kingdom, 2004-2017

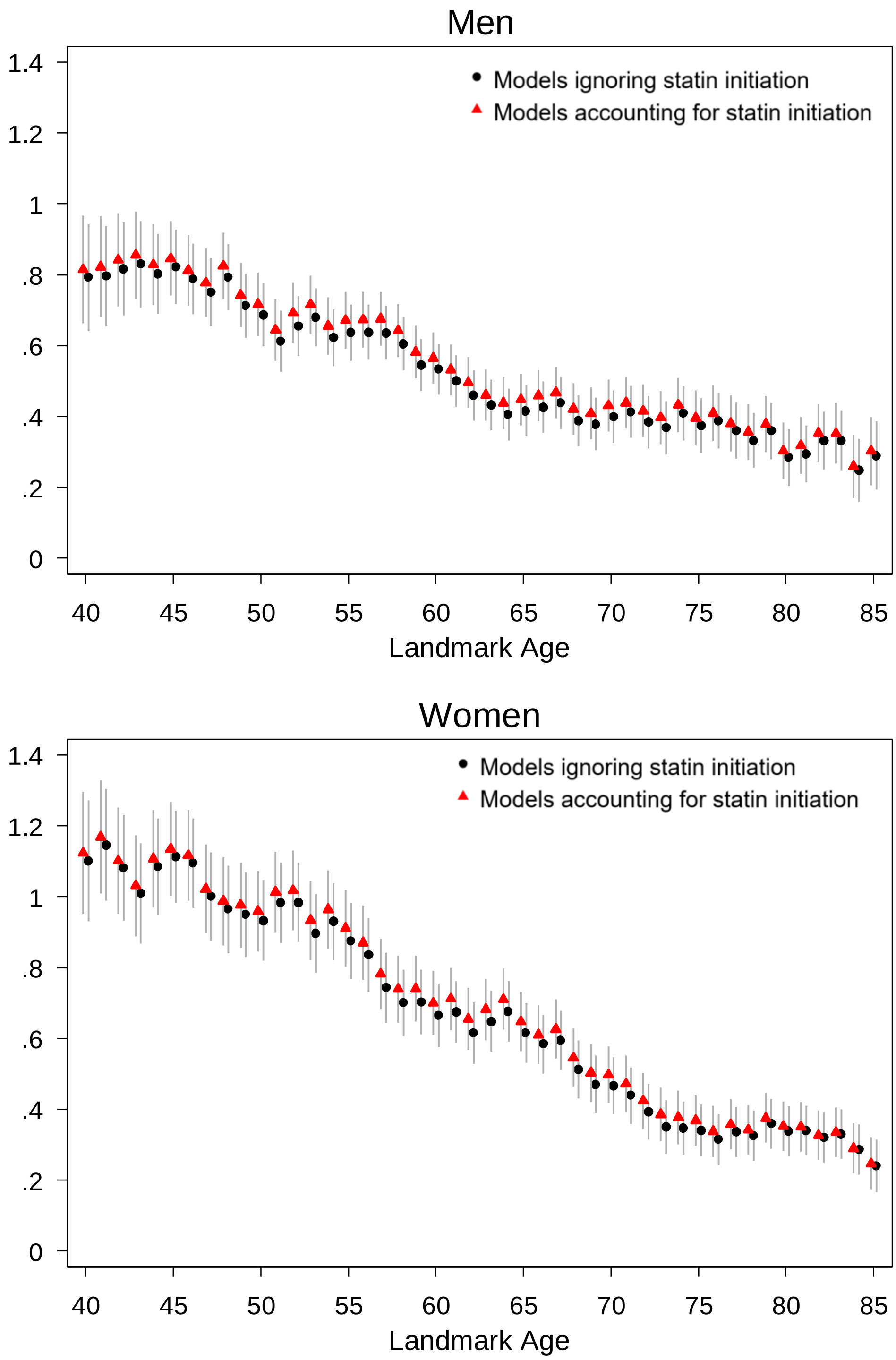

Web Figure 13. Comparison of D measures for models ignoring statin initiation versus models accounting for statin initiation for the prediction of 10-year cardiovascular disease at each landmark age in the validation dataset, Clinical Practice Research Datalink, Hospital Episode Statistics, and the Office for National Statistics, England, United Kingdom, 2004-2017

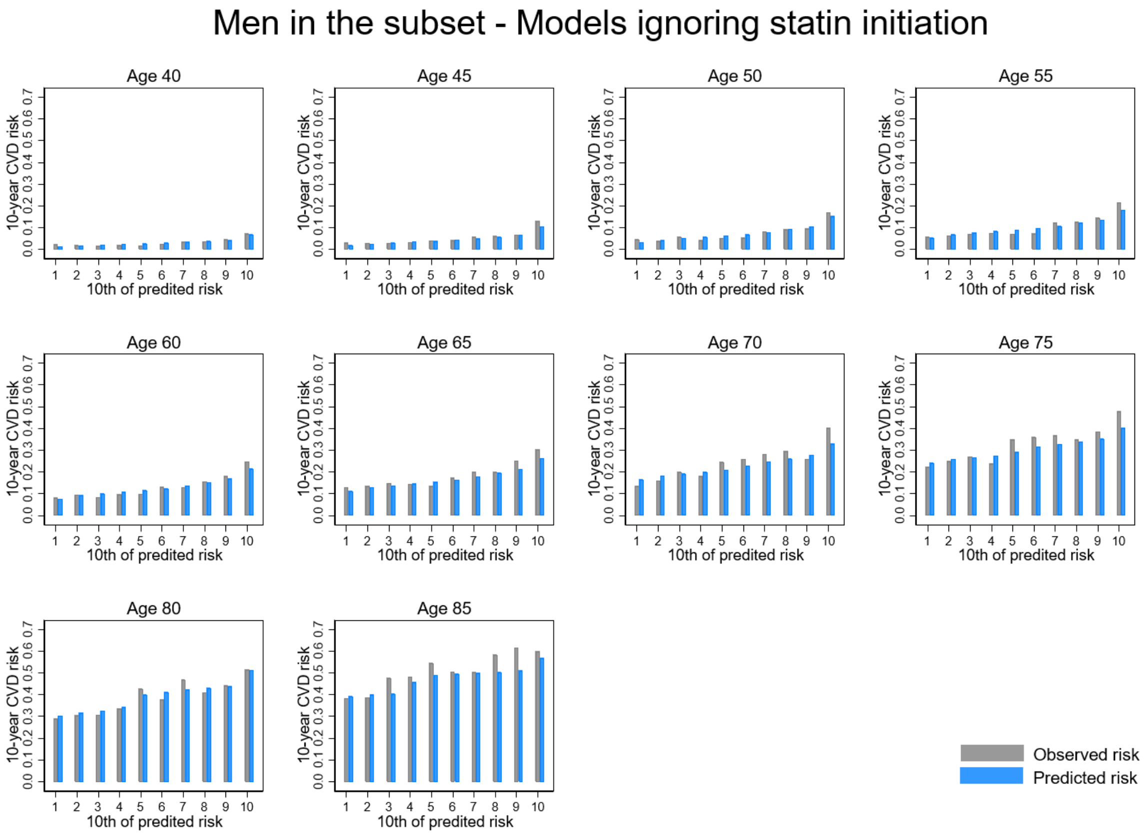

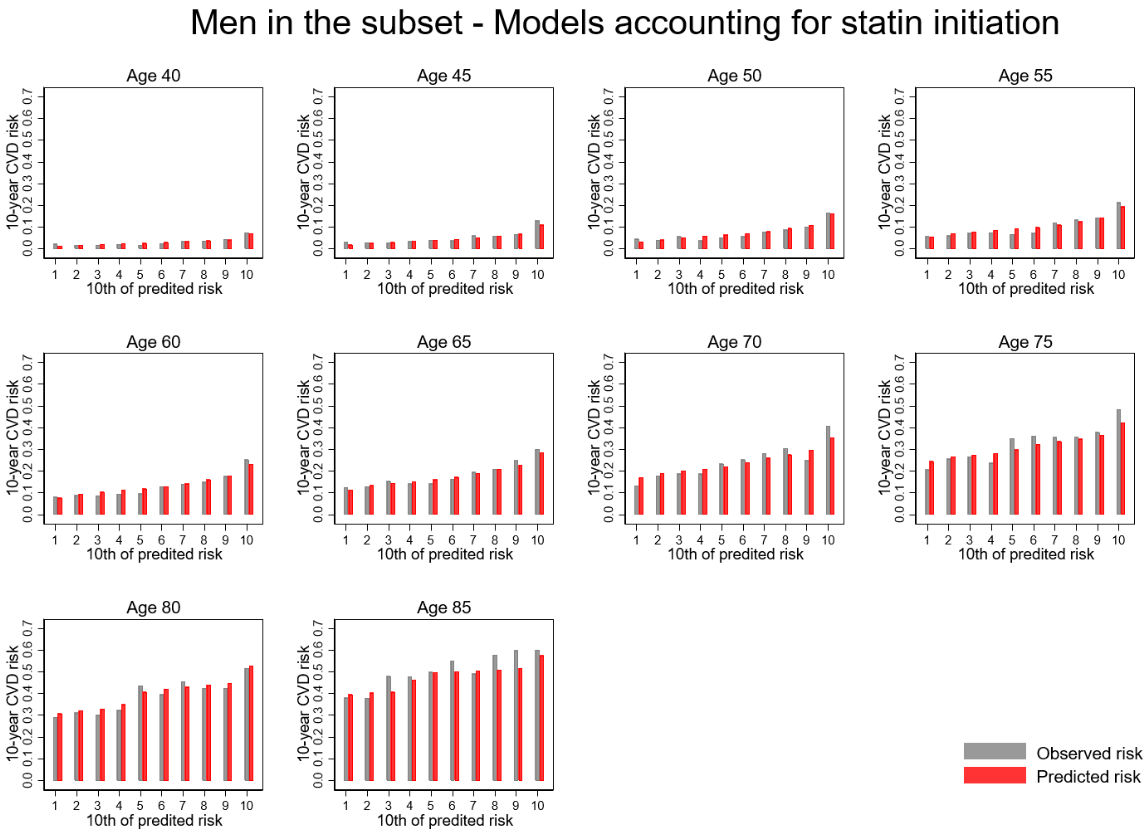

Web Figure 14. Calibration plots for men by landmark age in the validation subset of individuals who remained statin-naïve during follow-up, using models ignoring statin initiation and models accounting for statin initiation, Clinical Practice Research Datalink, Hospital Episode Statistics, and the Office for National Statistics, England, United Kingdom, 2004-2017. The plots show the mean predicted 10-year risk versus observed 10-year risk of cardiovascular disease, by deciles of predicted risk (in 5-year increments in landmark age for presentation).

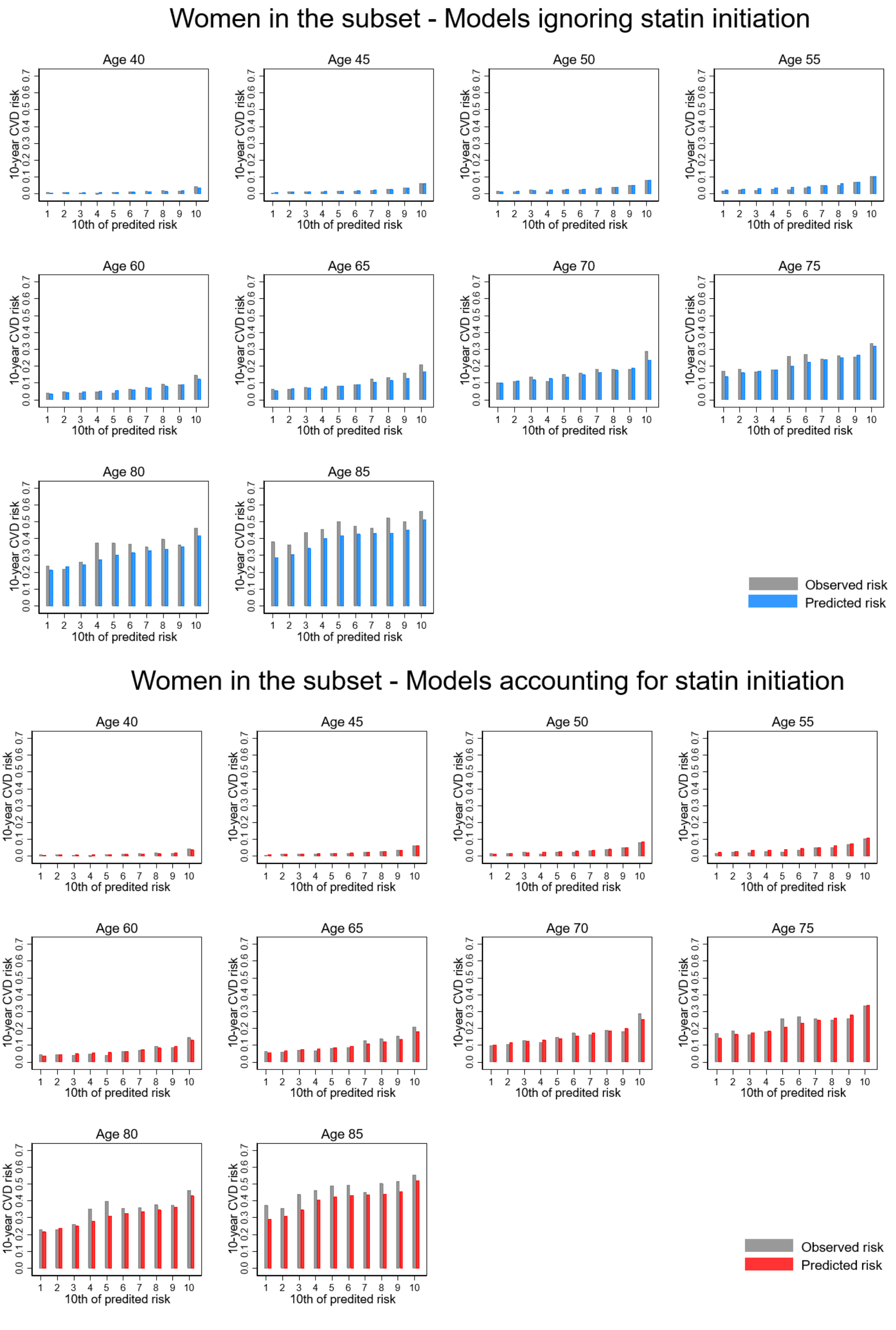

Web Figure 15. Calibration plots for women by landmark age in the validation subset of individuals who remained statin-naïve during follow-up, using models ignoring statin initiation and models accounting for statin initiation, Clinical Practice Research Datalink, Hospital Episode Statistics, and the Office for National Statistics, England, United Kingdom, 2004-2017. The plots show the mean predicted 10-year risk versus observed 10-year risk of cardiovascular disease, by deciles of predicted risk (in 5-year increments in landmark age for presentation).

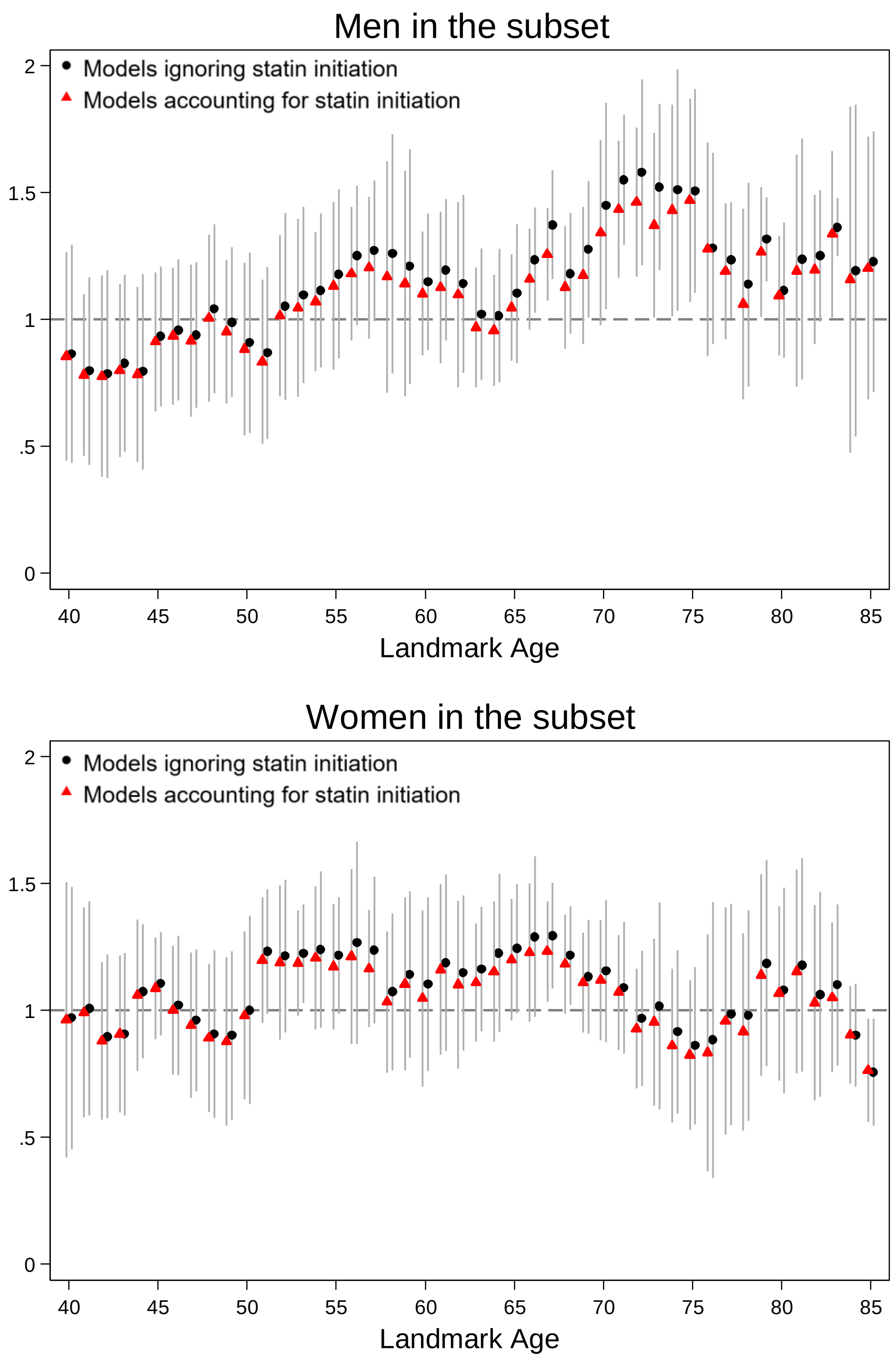

Web Figure 16. Comparison of calibration slopes for men and women in the validation subset of individuals who remained statin-naïve during follow-up, using models ignoring statin initiation versus models accounting for statin initiation for the prediction of 10-year cardiovascular disease risk at each landmark age in the validation dataset, Clinical Practice Research Datalink, Hospital Episode Statistics, and the Office for National Statistics, England, United Kingdom, 2004-2017. The calibration slope is the regression coefficient from the linear model: $\ln(-\ln(1-Y_{obs})) = \beta 0 + \beta 1 * \ln(-\ln(1-Y_{pred}))$, where Y_{obs} and Y_{pred} are the mean observed and predicted risks within each decile of predicted risk.

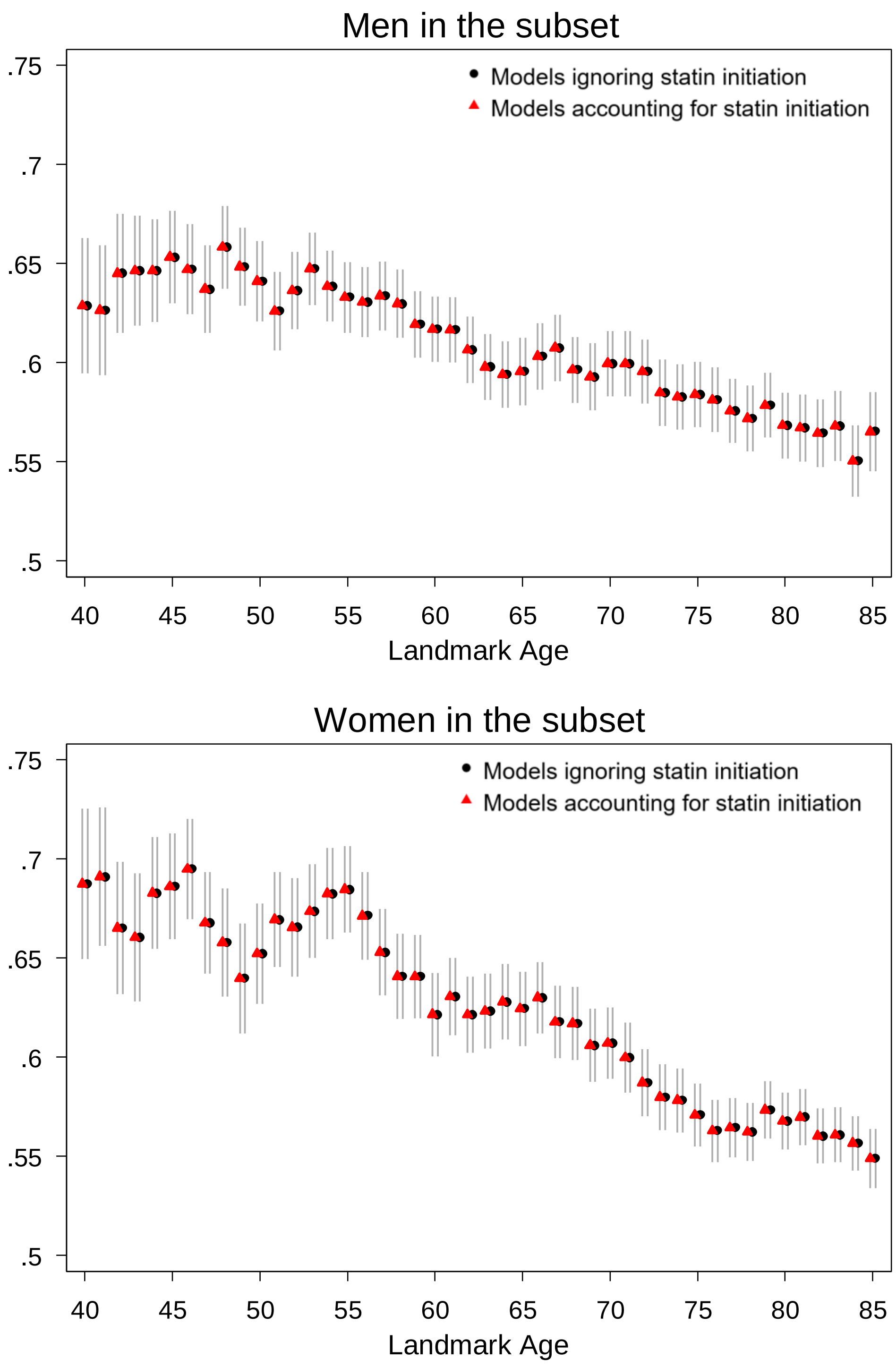

Web Figure 17. Comparision of C-indices for men and women in the validation subset of individuals who remained statin-naïve during follow-up, using models ignoring statin initiation versus models accounting for statin initiation for the prediction of 10-year cardiovascular disease risk at each landmark age in the validation dataset, Clinical Practice Research Datalink, Hospital Episode Statistics, and the Office for National Statistics, England, United Kingdom, 2004-2017

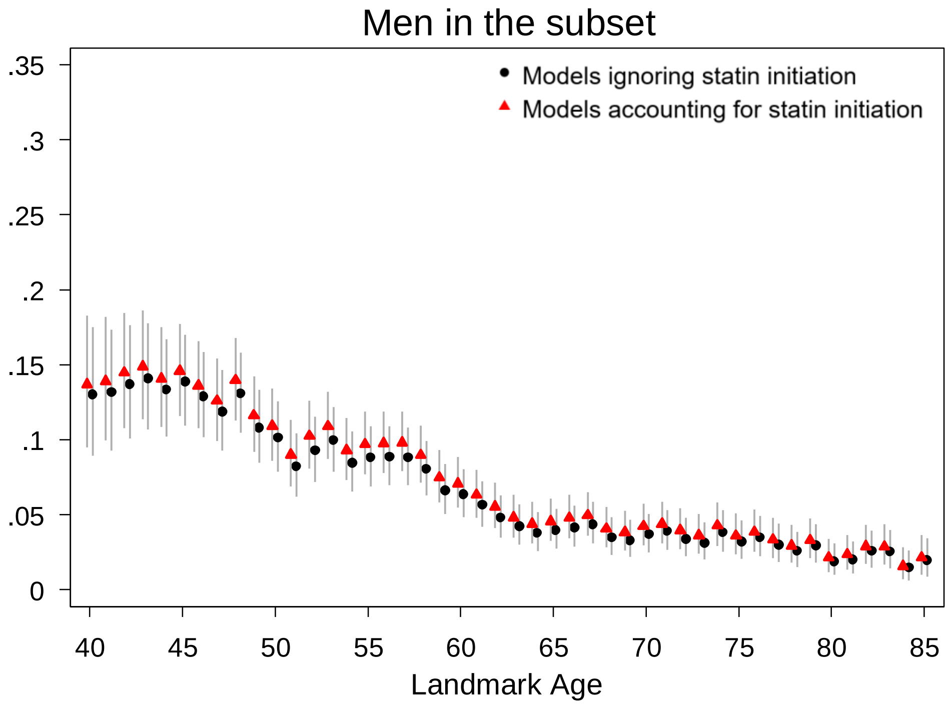

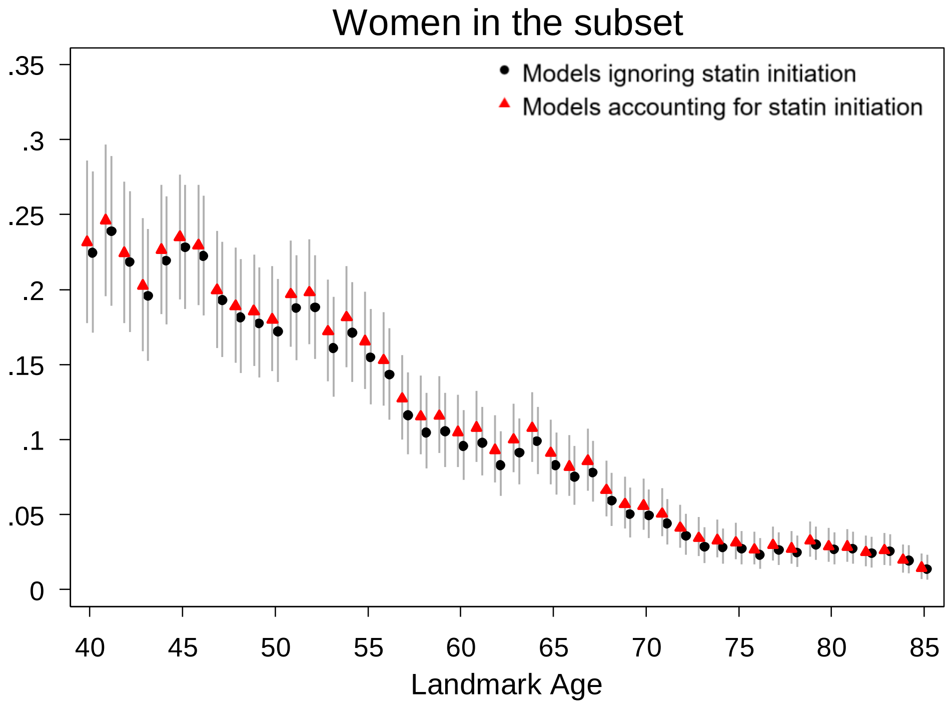

Web Figure 18. Comparison of R squared values for men and women in the validation subset of individuals who remained statin-naïve during follow-up, using models ignoring statin initiation versus models accounting for statin initiation for the prediction of 10-year cardiovascular disease risk at each landmark age in the validation dataset, Clinical Practice Research Datalink, Hospital Episode Statistics, and the Office for National Statistics, England, United Kingdom, 2004-2017

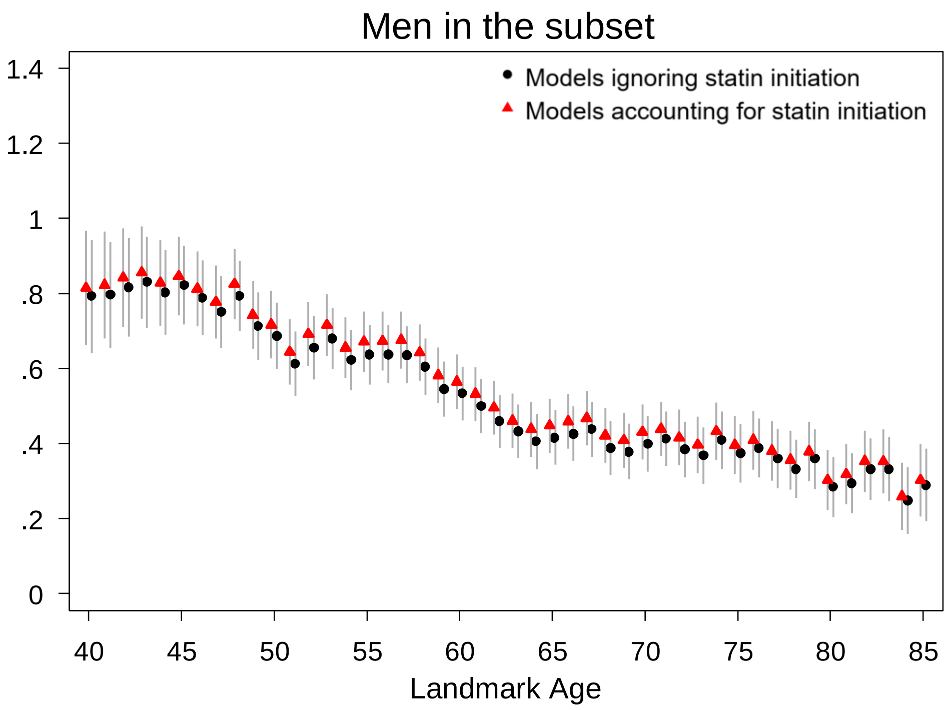

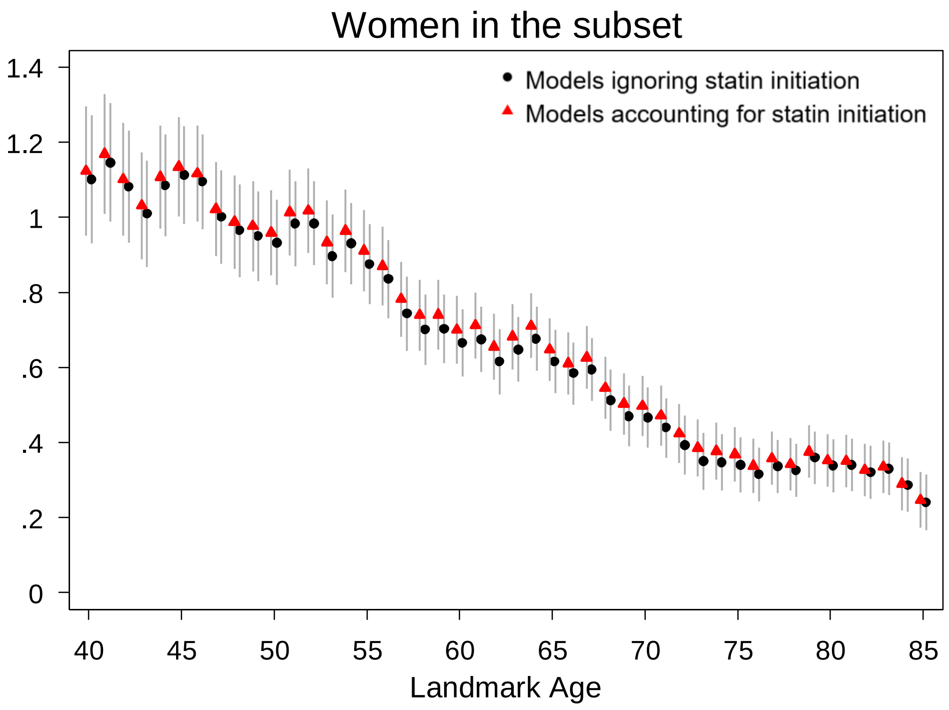

Web Figure 19. Comparison of D measures for men and women in the validation subset of individuals who remained statin-naïve during follow-up, using models ignoring statin initiation versus models accounting for statin initiation for the prediction of 10-year cardiovascular disease risk at each landmark age in the validation dataset, Clinical Practice Research Datalink, Hospital Episode Statistics, and the Office for National Statistics, England, United Kingdom, 2004-2017

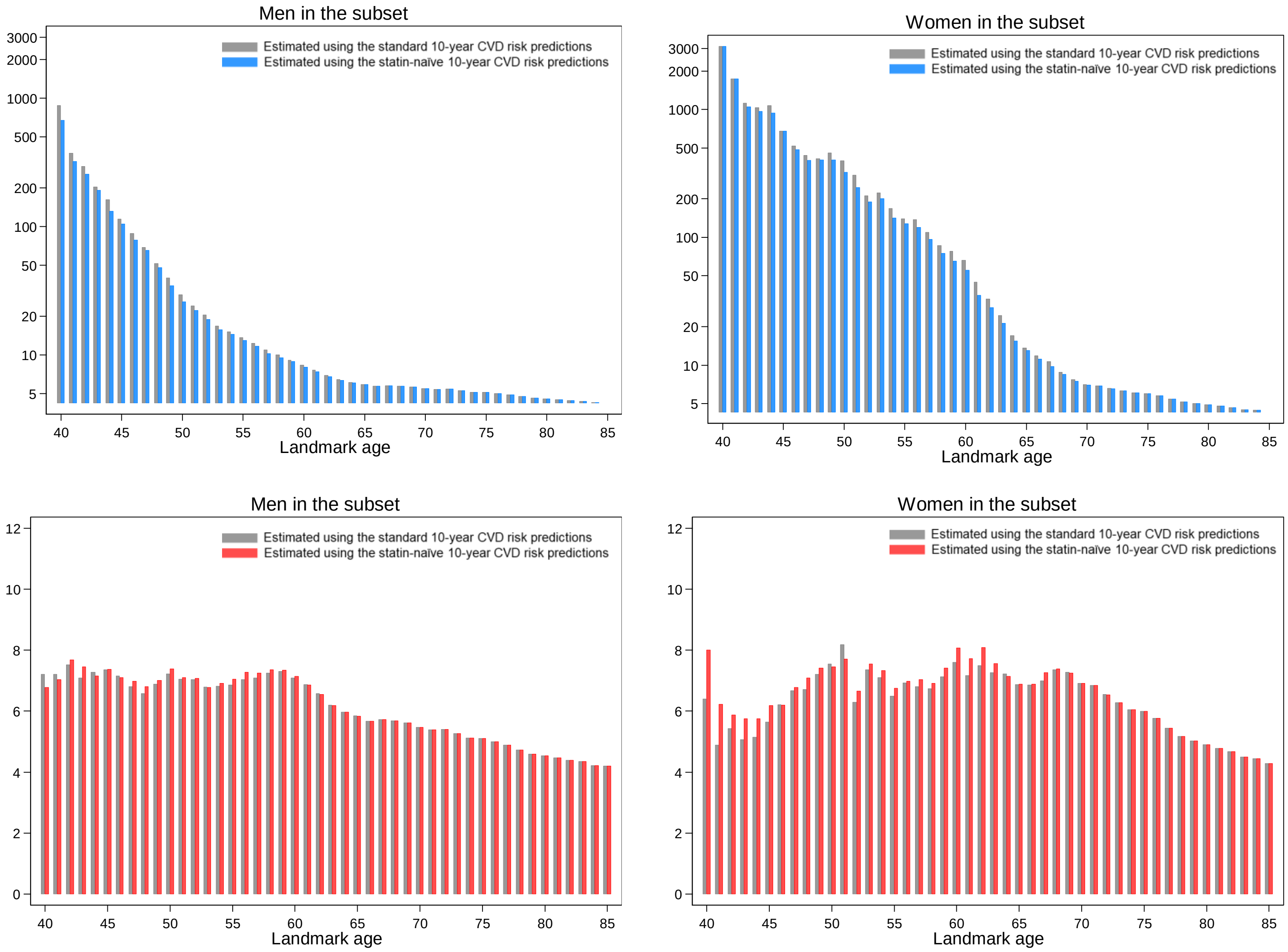

Web Figure 20. Number needed to screen (NNS) to prevent one cardiovascular disease event and number needed to treat (NNT) to prevent one cardiovascualr disease event in the validation subset of individuals who remained statin-naïve during follow-up, using standard 10-year cardiovascular risk predictions versus statin-naïve 10-year cardiovascular risk predictions, for men (left) and women (right) in the validation dataset, Clinical Practice Research Datalink, Hospital Episode Statistics, and the Office for National Statistics, England, United Kingdom, 2004-2017

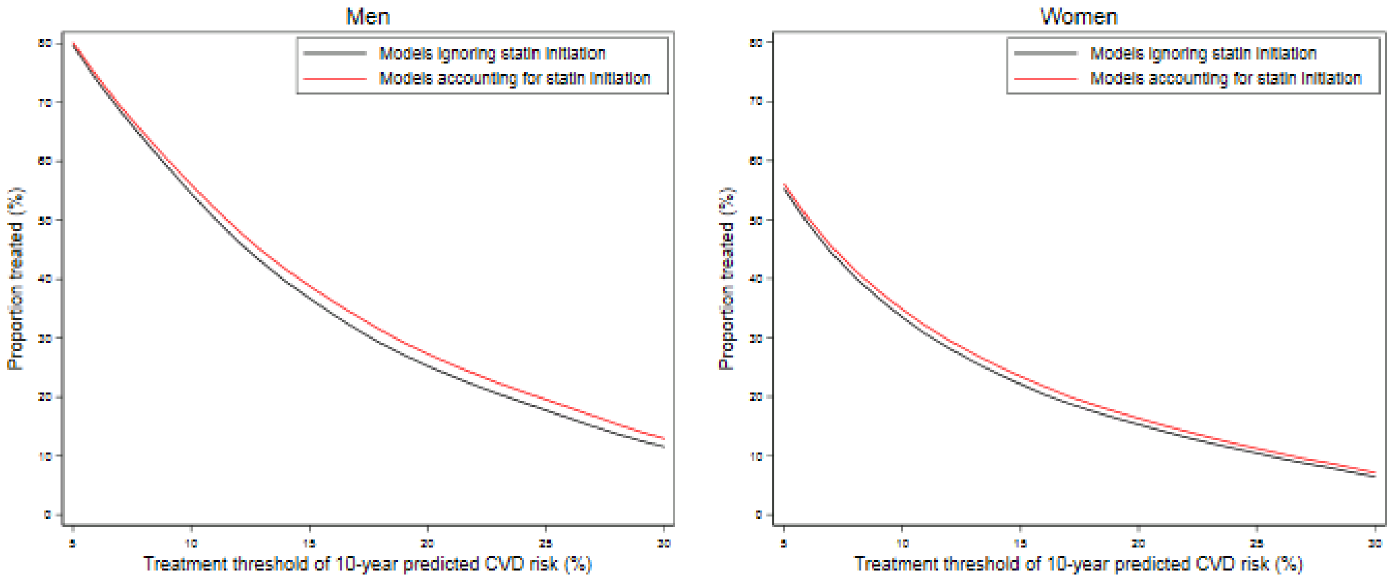

Web Figure 21. Proportion of individuals with 10-year predicted risk exceeding a range of treatment thresholds from 5% to 30% using the statin-naïve versus the standard CVD risk in the validation subset of individuals who remained statin-naïve during follow-up, Clinical Practice Research Datalink, Hospital Episode Statistics, and the Office for National Statistics, England, United Kingdom, 2004-2017. Weighted proportion across all ages were calculated using the most recent available data for an age-sex standard England population between 40-85 years.

References

1. Hippisley-Cox J, Coupland C, Brindle P. Development and validation of QRISK3 risk prediction algorithms to estimate future risk of cardiovascular disease: prospective cohort study. *BMJ*. 2017;357:j2099.

2. Herrett E, Shah AD, Boggon R, et al. Completeness and diagnostic validity of recording acute myocardial infarction events in primary care, hospital care, disease registry, and national mortality records: cohort study. *BMJ*. 2013;346:f2350.

3. Herrett E, Gallagher AM, Bhaskaran K, et al. Data resource profile: Clinical Practice Research Datalink (CPRD). *Int J Epidemiol*. 2015;44(3):827–836.

4. Paige E, Barrett J, Stevens D, et al. Landmark models for optimizing the use of repeated measurements of risk factors in electronic health records to predict future disease risk. *Am J Epidemiol*. 2018;187(7):1530–1538.

5. David C. Goff J, Lloyd-Jones DM, Bennett G, et al. 2013 ACC/AHA Guideline on the Assessment of Cardiovascular Risk. *Circulation* [electronic article]. 2014;(https://www.ahajournals.org/doi/abs/10.1161/01.cir.0000437741.48606.98). (Accessed April 29, 2019)

6. Sharma M, Petersen I, Nazareth I, et al. An algorithm for identification and classification of individuals with type 1 and type 2 diabetes mellitus in a large primary care database. *Clin Epidemiol*. 2016;8:373–380.

7. Verbeke G, Fieuws S, Molenberghs G, et al. The analysis of multivariate longitudinal data: A review. *Stat Methods Med Res*. 2014;23(1):42–59.

8. Joseph L. Analysis of incomplete multivariate data. CRC press; 1997 (Accessed March 5, 2020).(https://www.crcpress.com/Analysis-of-Incomplete-Multivariate-Data/Schafer/p/book/9780412040610). (Accessed March 5, 2020)

9. Fibrinogen Studies Collaboration. Correcting for multivariate measurement error by regression calibration in meta-analyses of epidemiological studies. *Stat Med*. 2009;28(7):1067–1092.

10. White I, Frost C, Tokunaga S. Correcting for measurement error in binary and continuous variables using replicates. *Stat Med*. 2001;20(22):3441–3457.

11. Goldberger AS. Best linear unbiased prediction in the generalized linear regression model. *Journal of the American Statistical Association*. 1962;57(298):369–375.

12. Goldstein H. Multilevel statistical models, 4th Edition. John Wiley & Sons, Ltd; 2010 (Accessed March 5, 2020).(https://www.wiley.com/en-us/Multilevel+Statistical+Models%2C+4th+Edition-p-9780470748657). (Accessed March 5, 2020)

13. Diggle P, Heagerty P, Liang K-Y, et al. Analysis of longitudinal data. Second Edition. Oxford, New York: Oxford University Press; 2013 400 p.

14. Cook NR, Ridker PM. Further insight into the cardiovascular risk calculator controversy: The roles of statins, revascularizations, and under-ascertainment in the Women's Health Study. *JAMA Intern Med*. 2014;174(12):1964–1971.

15. Collaborators CTT (CTT). The effects of lowering LDL cholesterol with statin therapy in people at low risk of vascular disease: meta-analysis of individual data from 27 randomised trials. *The Lancet*. 2012;380(9841):581–590.

16. Steyerberg E. Clinical prediction models: a practical approach to development, validation, and updating. New York: Springer-Verlag; 2009.

17. Royston P. Explained variation for survival models. *Stata Journal*. 2006;6(1):83–96.

18. Royston P, Sauerbrei W. A new measure of prognostic separation in survival data. *Statistics in Medicine*. 2004;23(5):723–748.

19. Harrell FE, Lee KL, Mark DB. Multivariable prognostic models: issues in developing models, evaluating assumptions and adequacy, and measuring and reducing errors. *Statistics in Medicine*. 1996;15(4):361–387.

20. Steyerberg EW, Vickers AJ, Cook NR, et al. Assessing the performance of prediction models: a framework for traditional and novel measures. *Epidemiology*. 2010;21(1):128–138.

21. Hilden J, Habbema JD, Bjerregaard B. The measurement of performance in probabilistic diagnosis. II. Trustworthiness of the exact values of the diagnostic probabilities. *Methods Inf Med*. 1978;17(4):227–237.

22. Miller ME, Langefeld CD, Tierney WM, et al. Validation of probabilistic predictions. *Med Decis Making*. 1993;13(1):49–57.

23. Pennells L, Kaptoge S, Wood A, et al. Equalization of four cardiovascular risk algorithms after systematic recalibration: individual-participant meta-analysis of 86 prospective studies. *Eur Heart J*. 2019;40(7):621–631.

24. Parzen M, Lipsitz SR. A global goodness-of-fit statistic for Cox regression models. *Biometrics*. 1999;55(2):580–584.

25. National Institute for Health and Care Excellence. (2014) Cardiovascular disease: risk assessment and reduction, including lipid modification. (https://www.nice.org.uk/guidance/cg181). (Accessed May 5, 2020)

26. Pencina MJ, Agostino RBD, Agostino RBD, et al. Evaluating the added predictive ability of a new marker: From area under the ROC curve to reclassification and beyond. *Statistics in Medicine*. 2008;27(2):157–172.

27. Pencina MJ, D'Agostino RB, Steyerberg EW. Extensions of net reclassification improvement calculations to measure usefulness of new biomarkers. *Stat Med*. 2011;30(1):11–21.

28. Pencina MJ, Demler OV. Novel metrics for evaluating improvement in discrimination: net reclassification and integrated discrimination improvement for normal variables and nested models. *Stat Med*. 2012;31(2):101–113.

29. Rembold CM. Number needed to screen: development of a statistic for disease screening. *BMJ*. 1998;317(7154):307–312.

30. Rembold CM. Number-needed-to-treat analysis of the prevention of myocardial infarction and death by antidyslipidemic therapy. *J Fam Pract*. 1996;42(6):577–586.

31. Population by age, gender and ethnicity - Office for National Statistics. (https://www.ons.gov.uk/aboutus/transparencyandgovernance/freedomofinformationfoi/populationbyagegenderandethnicity). (Accessed September 29, 2020)
